# Supplementary material for: An experimentally validated network of nine haematopoietic transcription factors reveals mechanisms of cell state stability
Source: eLife. 2016 Feb 22;5:e11469. doi: 10.7554/eLife.11469 (PMC4798972; doi:10.7554/eLife.11469)
Supplement: Figure 6—source data 1. — The results for a total of 162 simulations are shown. The data can be accessed using the embedded hyperlinks. The y-axes show the number of cells and the x-axes the relative expression level. Blue curves represent wild-type data and red curves represent perturbation data. DOI: http://dx.doi.org/10.7554/eLife.11469.051 [file elife-11469-fig6-data1.zip › Figure6_sourcedata_1.pdf]

Figure 6 – source data 1: Summary of all computational simulations for perturbations of one or two TFs.

**Erg**	Down-regulation	Up-regulation
**Fli1**	Down-regulation	Up-regulation
**Gata2**	Down-regulation	Up-regulation
**Gfi1b**	Down-regulation	Up-regulation
**Lyl1**	Down-regulation	Up-regulation
**Meis1**	Down-regulation	Up-regulation
**Spi1/PU.1**	Down-regulation	Up-regulation
**Runx1**	Down-regulation	Up-regulation
**Tal1/Scl**	Down-regulation	Up-regulation

	**Erg**	**Fli1**	**Gata2**	**Gfi1b**	**Lyl1**	**Meis1**	**Spi1/ PU.1**	**Runx1**	**Tal1/ Scl**
**Erg**		down-down	down-down	down-down	down-down	down-down	down-down	down-down	down-down
**Fli1**	up-up		down-down	down-down	down-down	down-down	down-down	down-down	down-down
**Gata2**	up-up	up-up		down-down	down-down	down-down	down-down	down-down	down-down
**Gfi1b**	up-up	up-up	up-up		down-down	down-down	down-down	down-down	down-down
**Lyl1**	up-up	up-up	up-up	up-up		down-down	down-down	down-down	down-down
**Meis1**	up-up	up-up	up-up	up-up	up-up		down-down	down-down	down-down
**Spi1/ PU.1**	up-up	up-up	up-up	up-up	up-up	up-up		down-down	down-down
**Runx1**	up-up	up-up	up-up	up-up	up-up	up-up	up-up		down-down
**Tal1/ Scl**	up-up	up-up	up-up	up-up	up-up	up-up	up-up	up-up	

	**Erg**	**Fli1**	**Gata2**	**Gfi1b**	**Lyl1**	**Meis1**	**Spi1/ PU.1**	**Runx1**	**Tal1/ Scl**
**Erg**		down-up	down-up	down-up	down-up	down-up	down-up	down-up	down-up
**Fli1**	down-up		down-up	down-up	down-up	down-up	down-up	down-up	down-up
**Gata2**	down-up	down-up		down-up	down-up	down-up	down-up	down-up	down-up
**Gfi1b**	down-up	down-up	down-up		down-up	down-up	down-up	down-up	down-up
**Lyl1**	down-up	down-up	down-up	down-up		down-up	down-up	down-up	down-up
**Meis1**	down-up	down-up	down-up	down-up	down-up		down-up	down-up	down-up
**Spi1/ PU.1**	down-up	down-up	down-up	down-up	down-up	down-up		down-up	down-up
**Runx1**	down-up	down-up	down-up	down-up	down-up	down-up	down-up		down-up
**Tal1/ Scl**	down-up	down-up	down-up	down-up	down-up	down-up	down-up	down-up	

Control Perturbation

Single TF perturbation

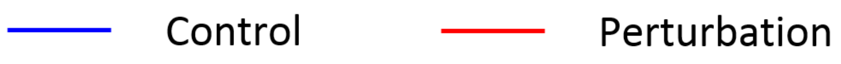
Control
Perturbation

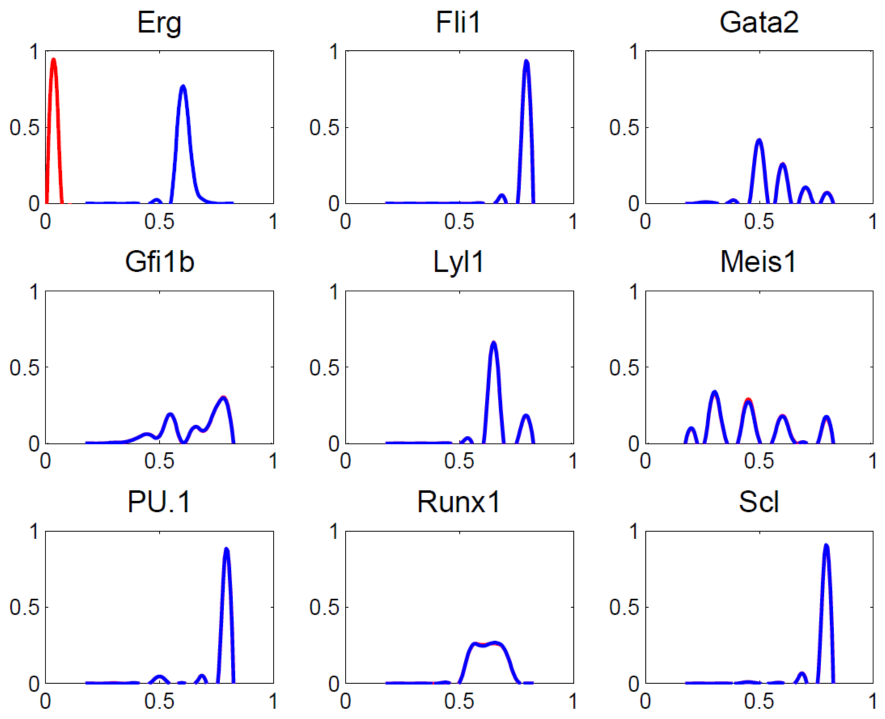
Erg
Fli1
Gata2
Gfi1b
Lyl1
Meis1
PU.1
Runx1
Scl

top

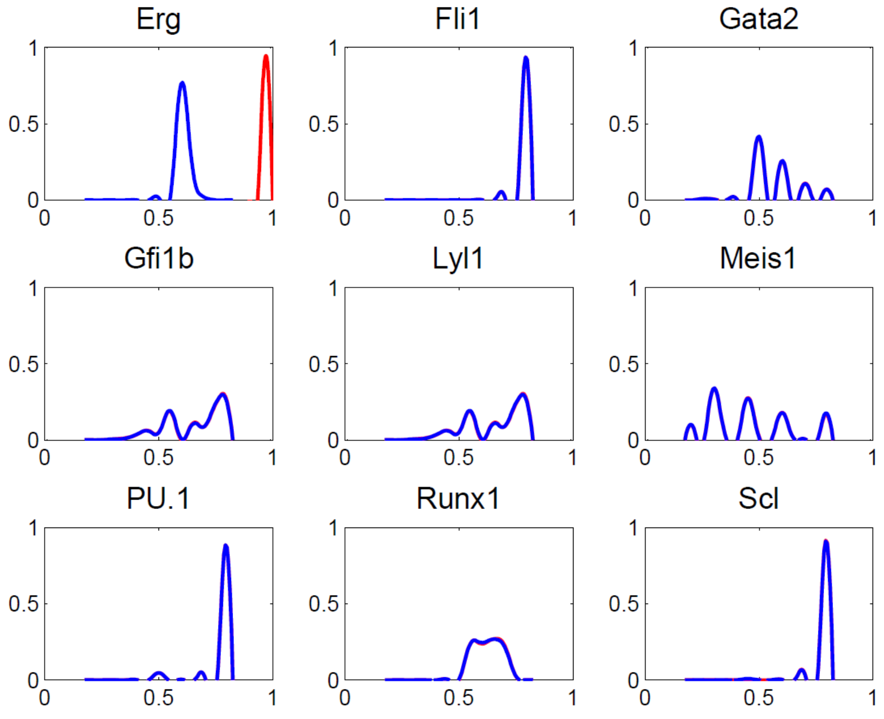
Erg
Fli1
Gata2
Gfi1b
Lyl1
Meis1
PU.1
Runx1
Scl

top

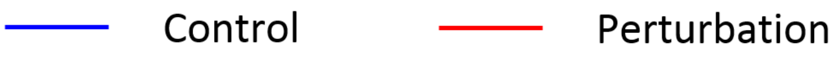
Control
Perturbation

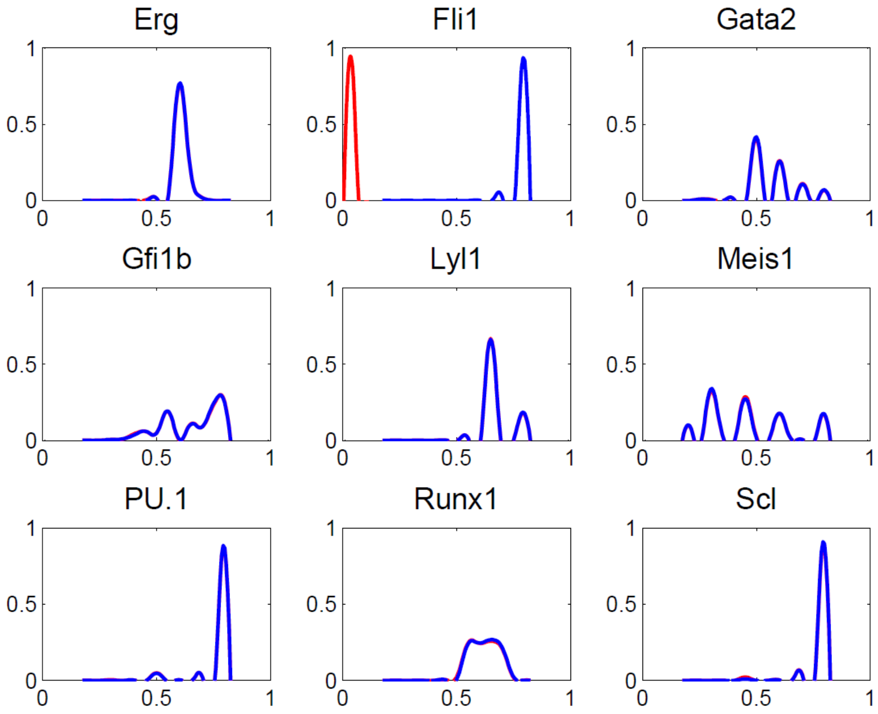
Erg
Fli1
Gata2
Gfi1b
Lyl1
Meis1
PU.1
Runx1
Scl
0
0.5
1

top

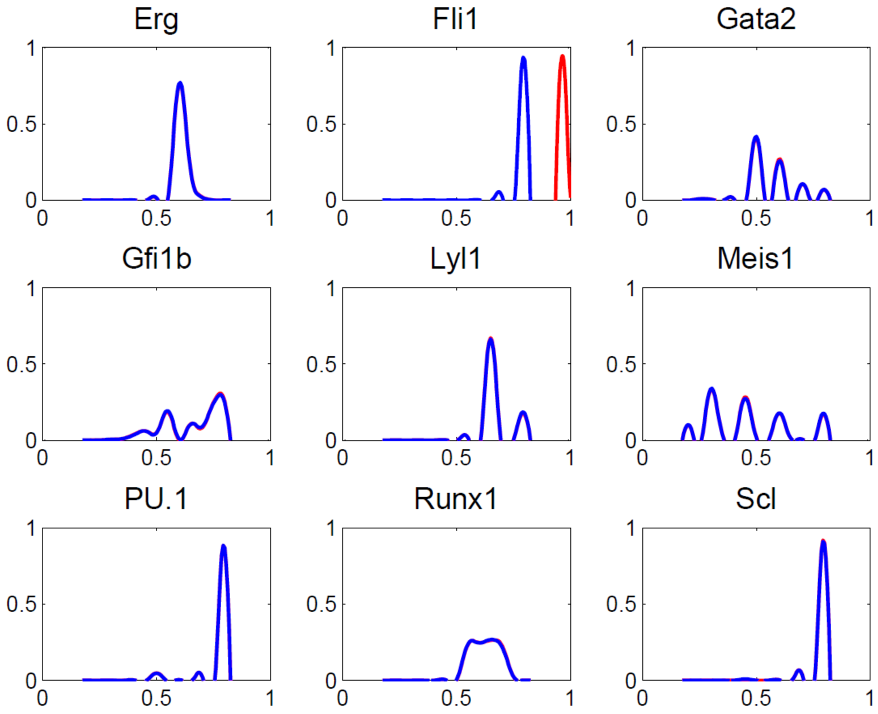
Erg
Fli1
Gata2
Gfi1b
Lyl1
Meis1
PU.1
Runx1
Scl
0
0.5
1

top

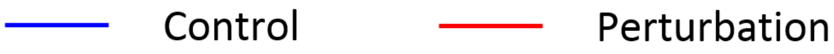
Control
Perturbation

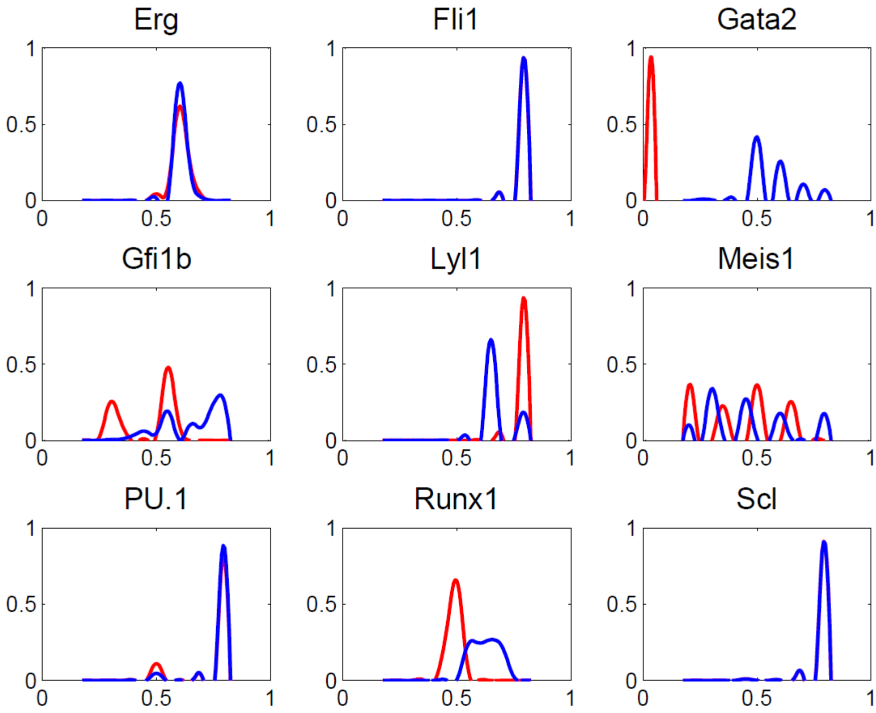
Erg
Fli1
Gata2
Gfi1b
Lyl1
Meis1
PU.1
Runx1
Scl

top

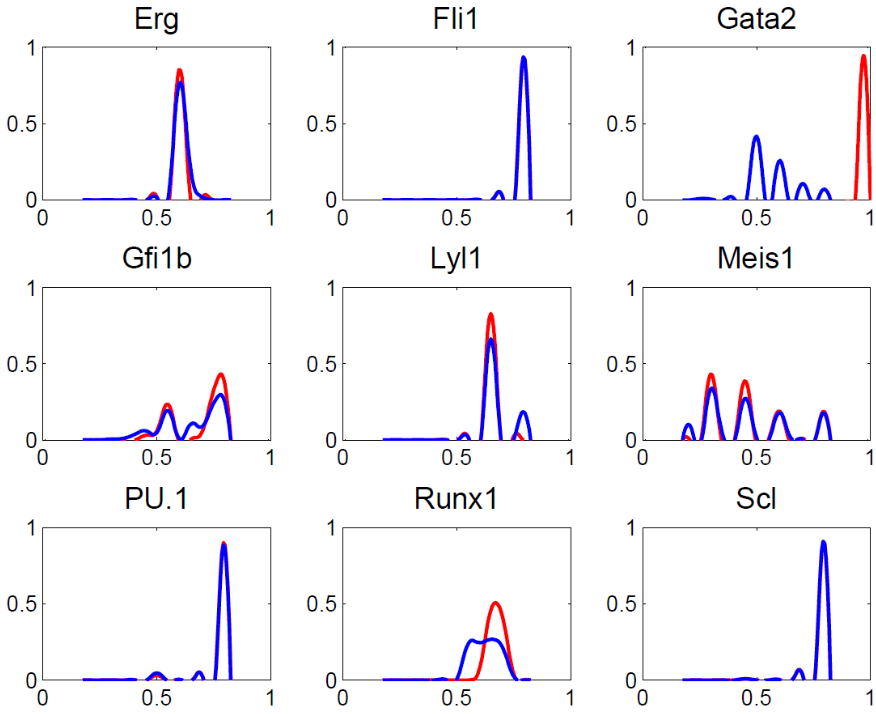
Erg
Fli1
Gata2
Gfi1b
Lyl1
Meis1
PU.1
Runx1
Scl

top

Control Perturbation

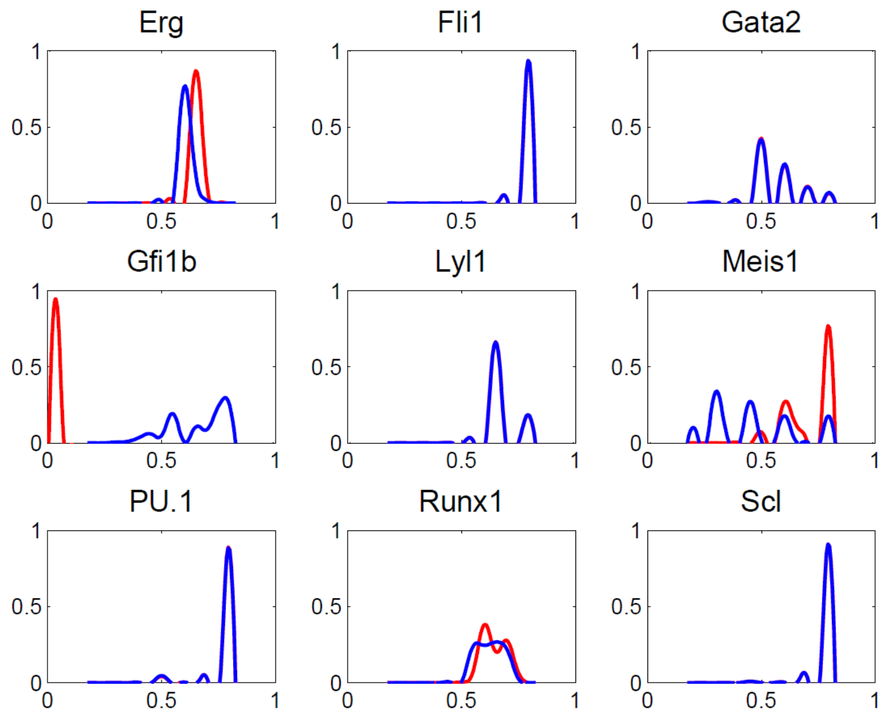

top

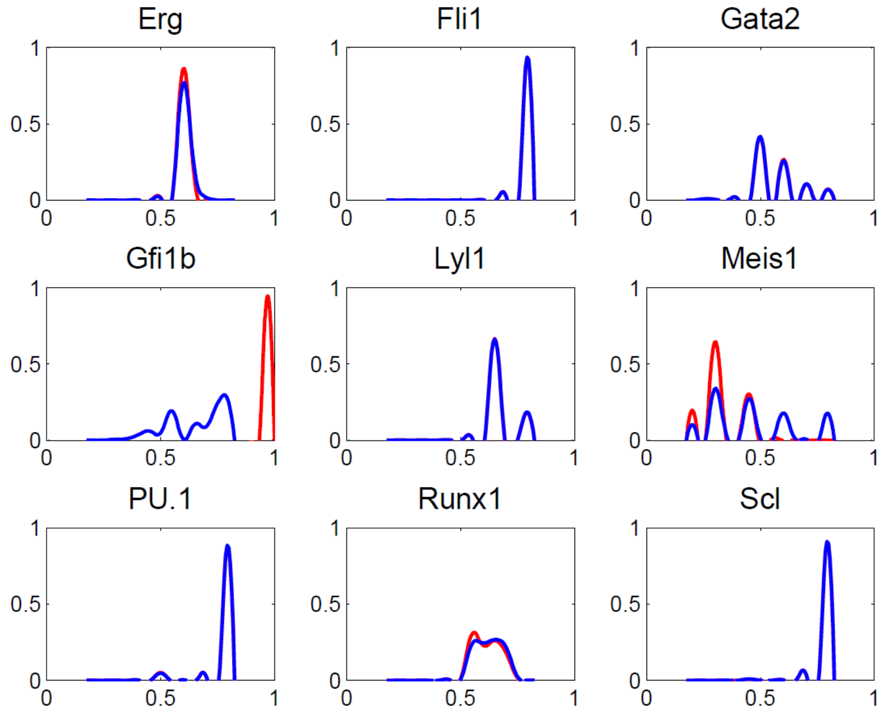

top

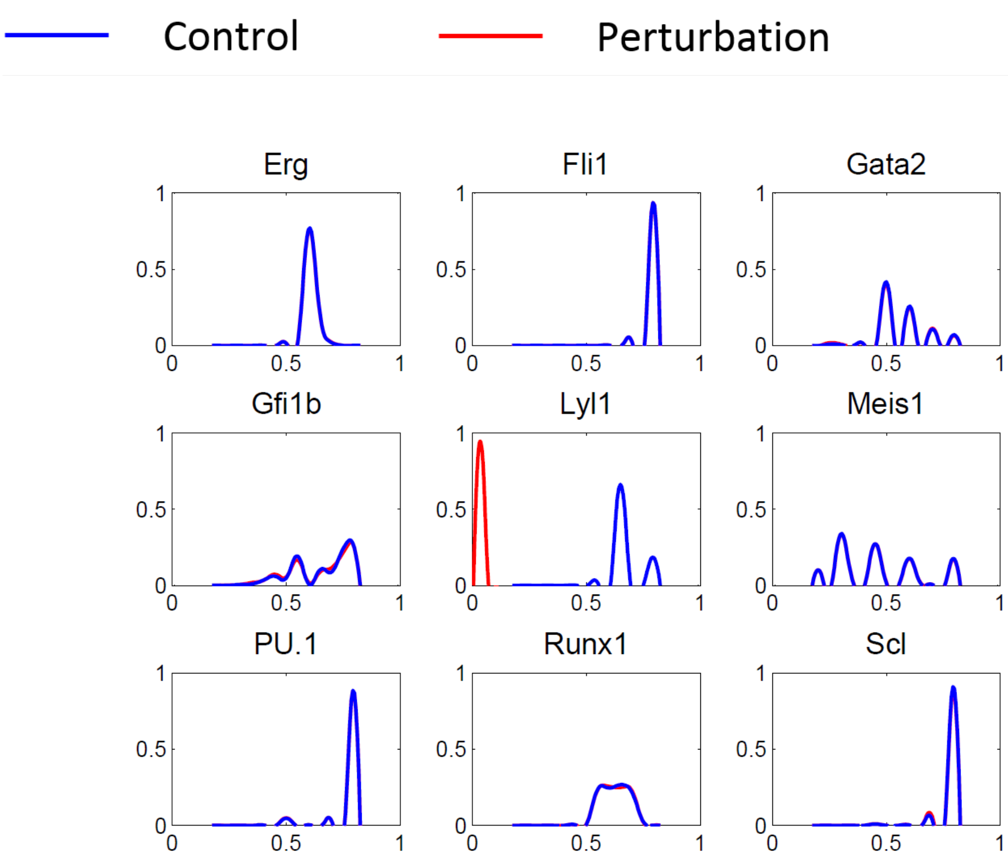
Control
Perturbation
Erg
Fli1
Gata2
Gfi1b
Lyl1
Meis1
PU.1
Runx1
Scl
0
0.5
1

top

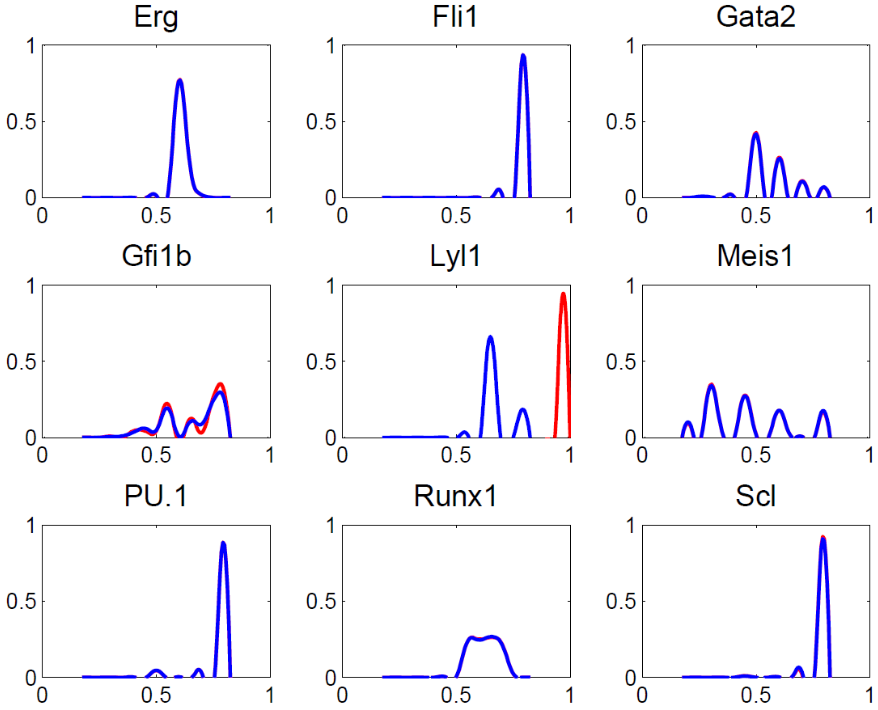
Erg
Fli1
Gata2
Gfi1b
Lyl1
Meis1
PU.1
Runx1
Scl
0
0.5
1

top

Control Perturbation

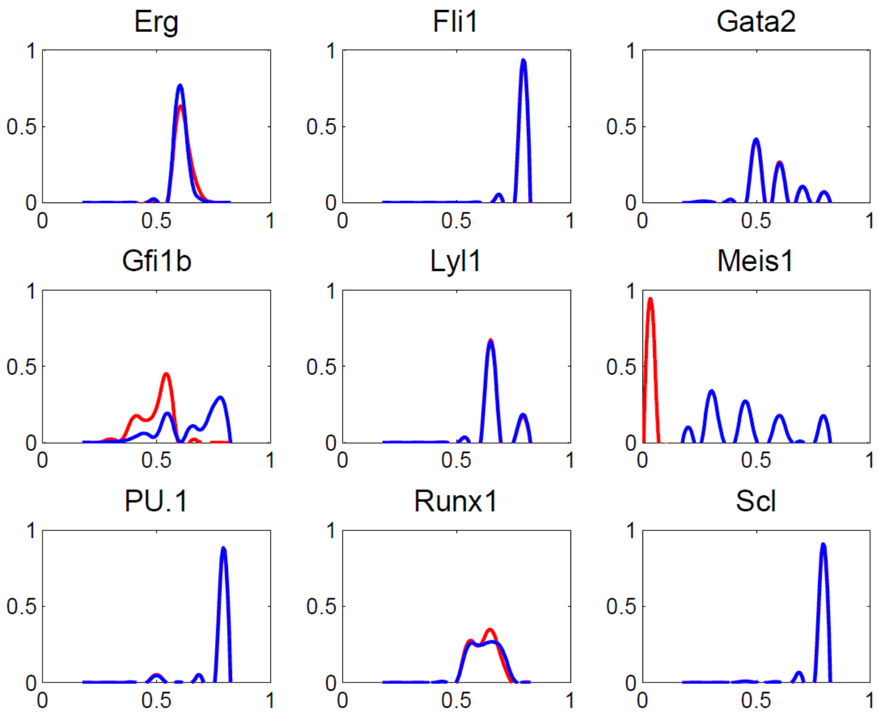

top

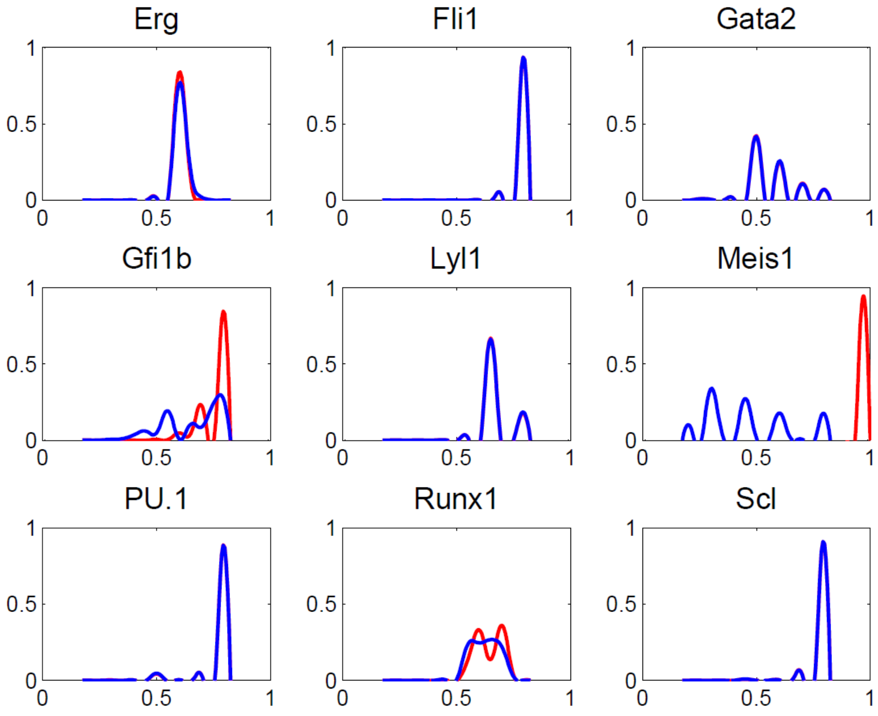

top

Control Perturbation

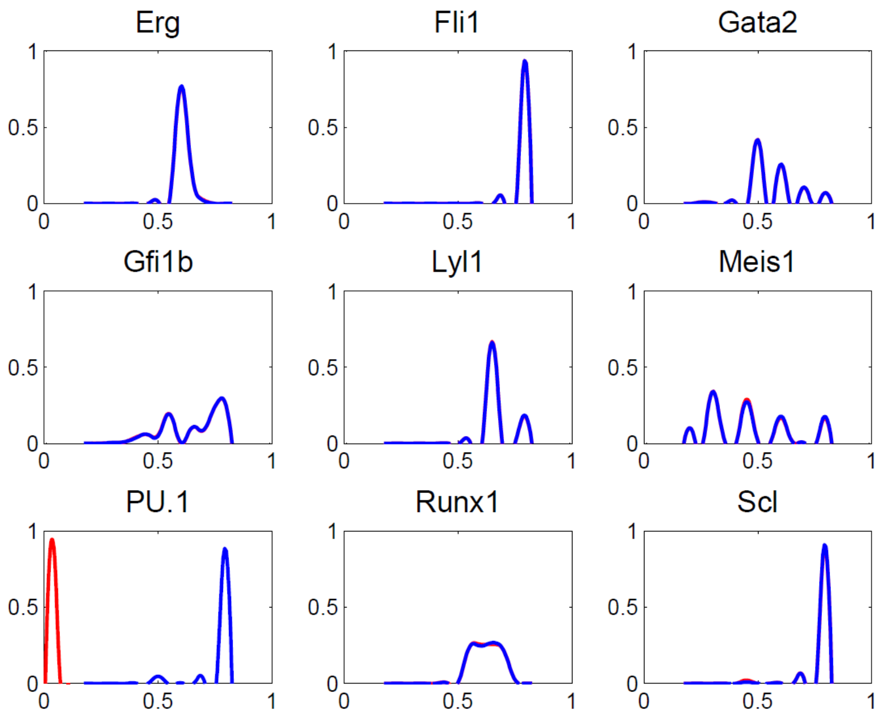

top

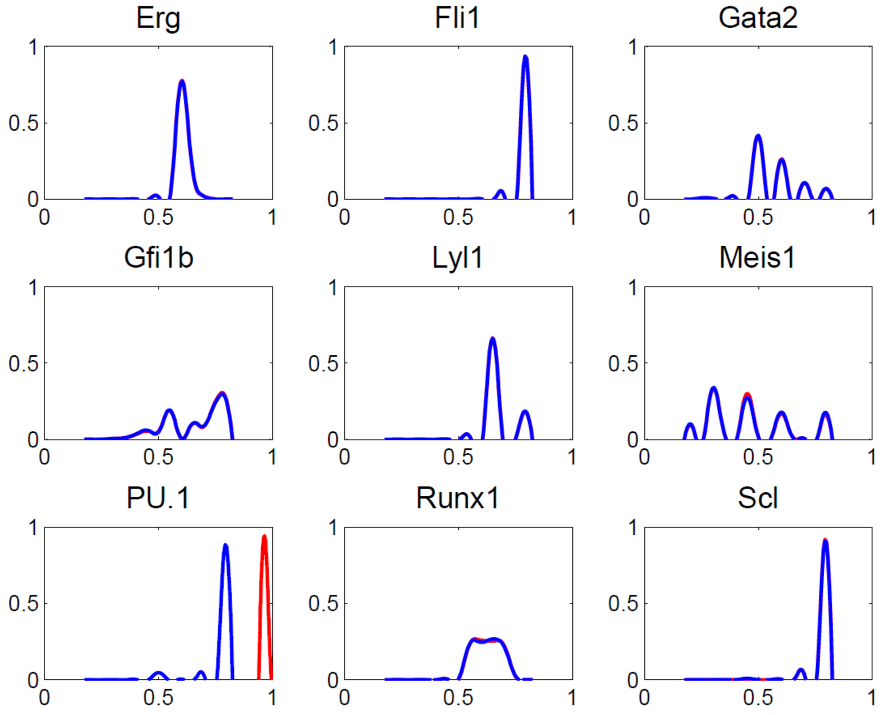

top

Control Perturbation

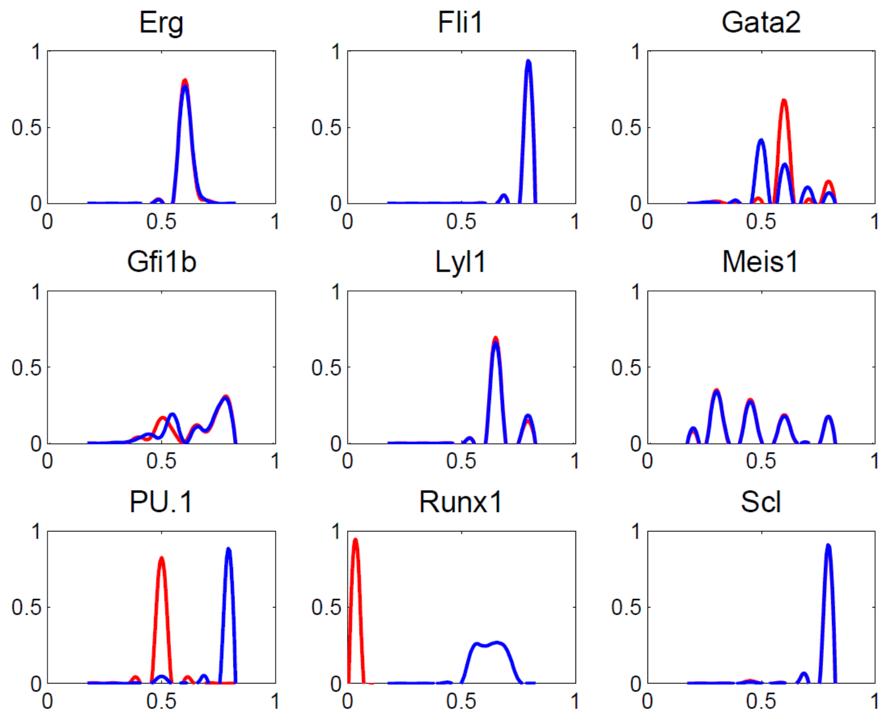

top

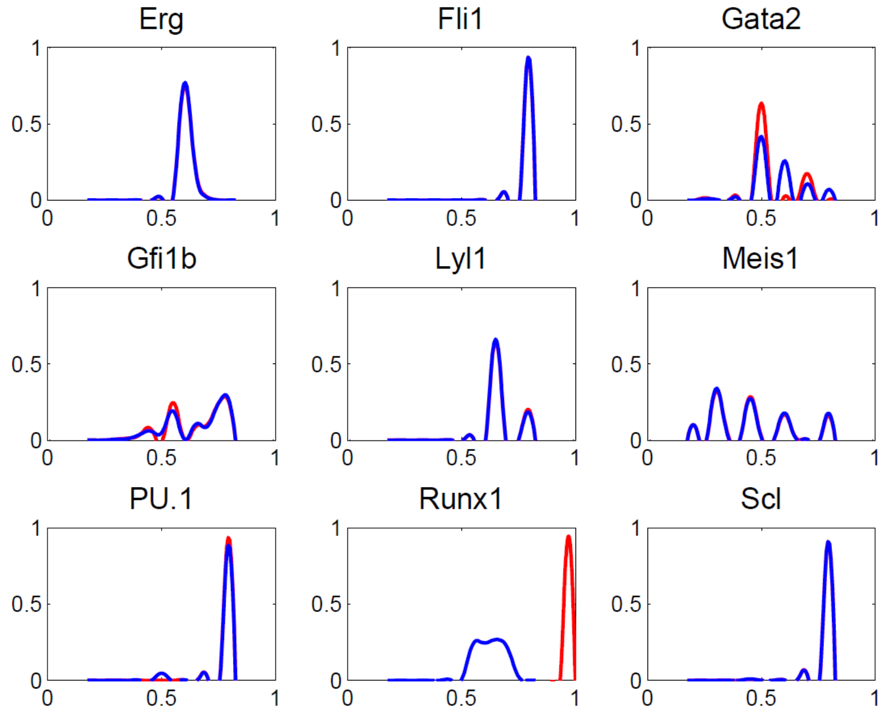

top

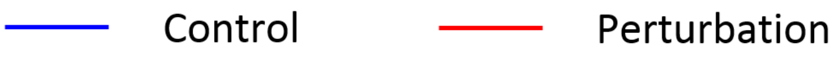
Control
Perturbation

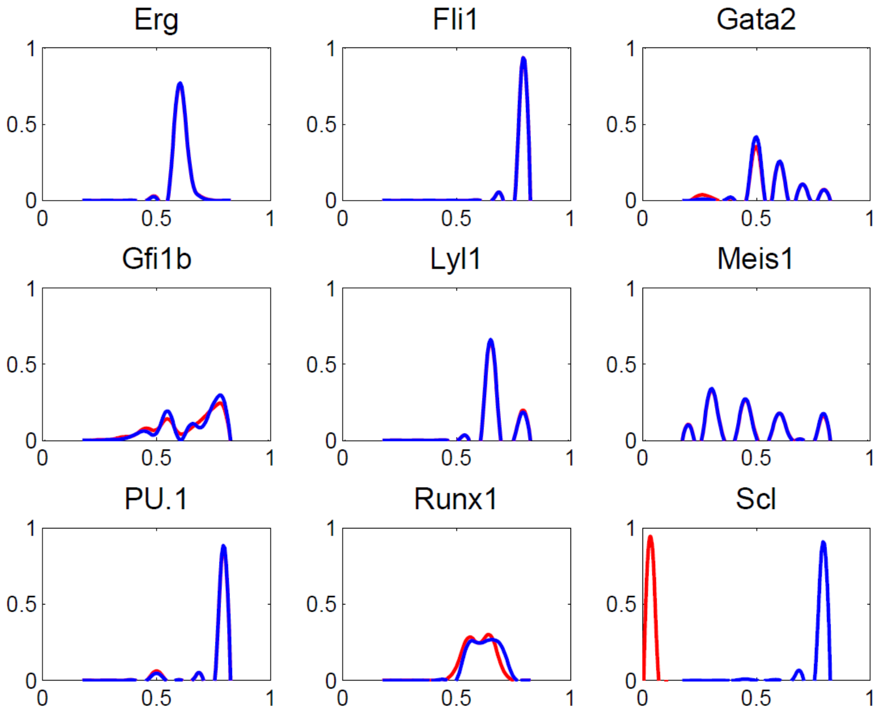
Erg
Fli1
Gata2
Gfi1b
Lyl1
Meis1
PU.1
Runx1
Scl

top

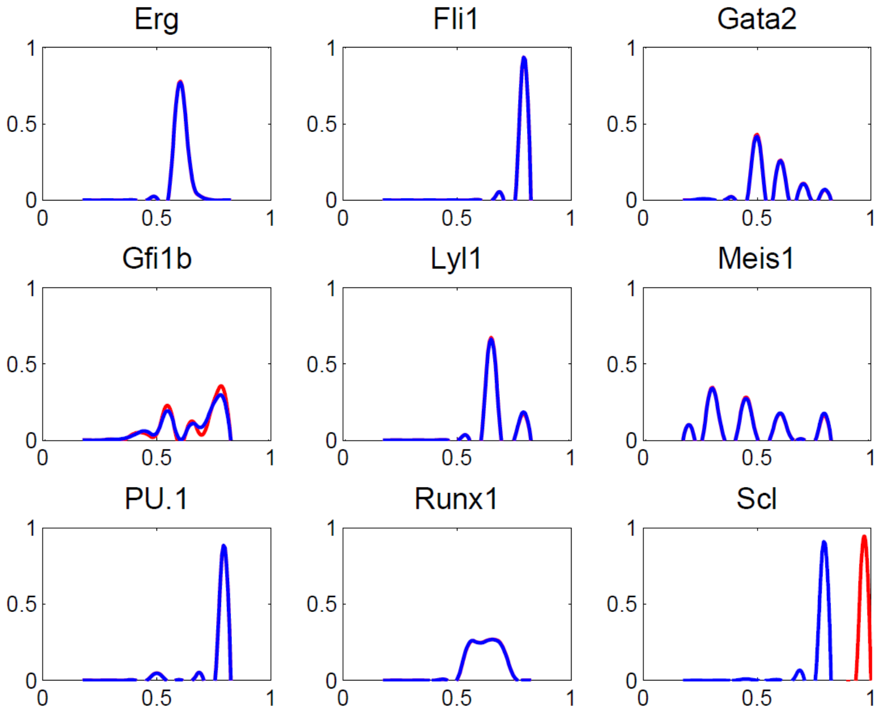
Erg
Fli1
Gata2
Gfi1b
Lyl1
Meis1
PU.1
Runx1
Scl

top

Control Perturbation

In each case:
two TFs up or down

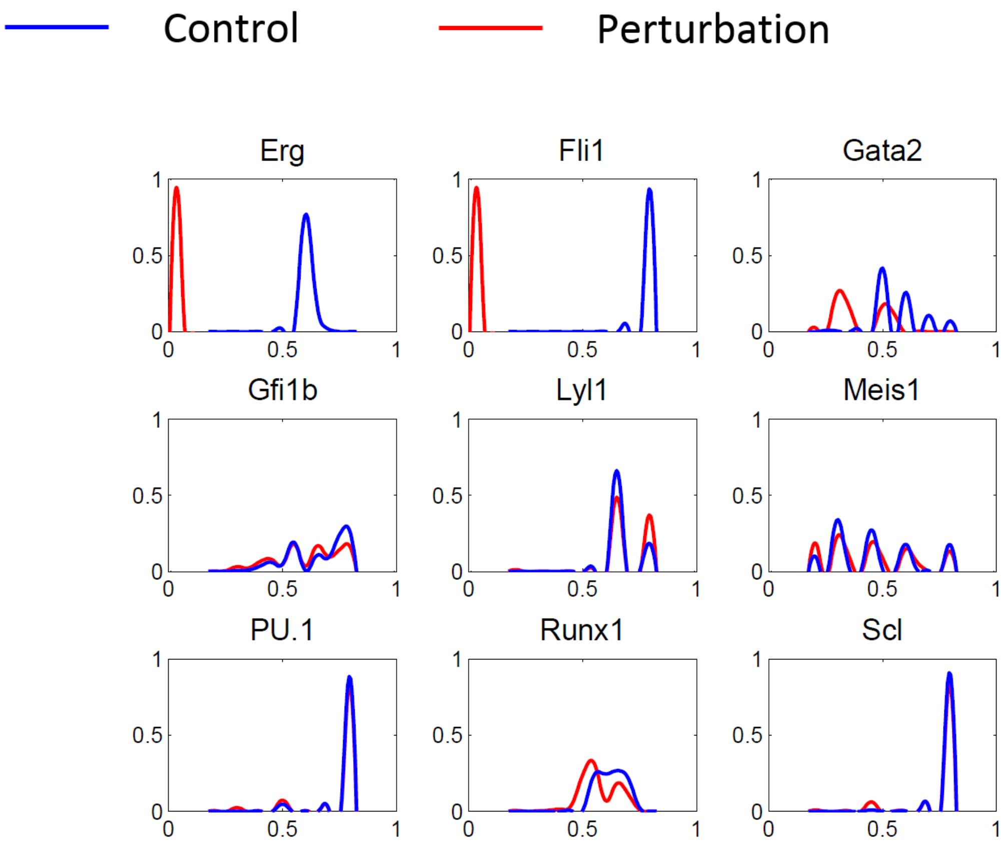
Control
Perturbation
Erg
Fli1
Gata2
Gfi1b
Lyl1
Meis1
PU.1
Runx1
Scl

Top

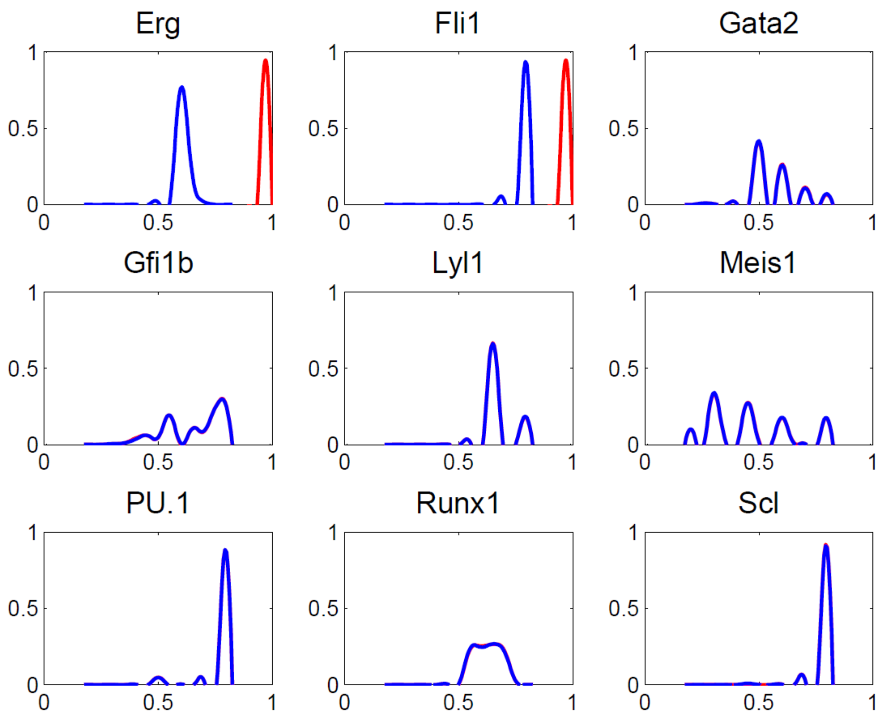
Erg
Fli1
Gata2
Gfi1b
Lyl1
Meis1
PU.1
Runx1
Scl

Top

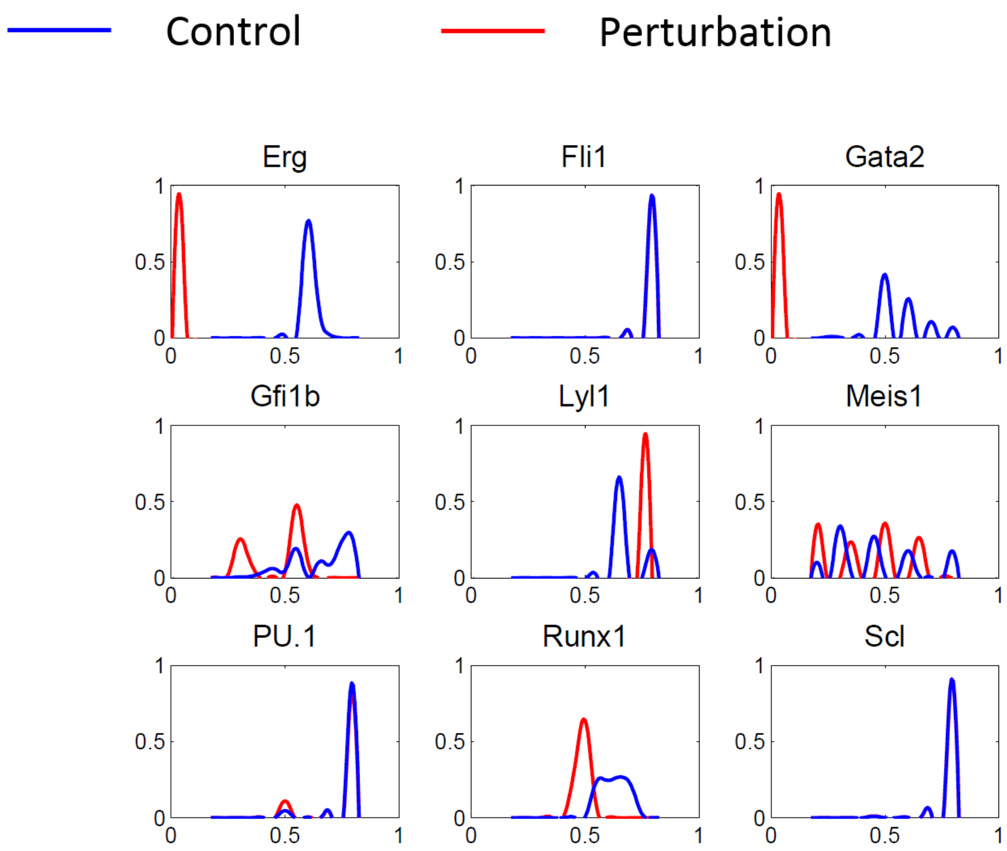
Control
Perturbation
Erg
Fli1
Gata2
Gfi1b
Lyl1
Meis1
PU.1
Runx1
Scl

Top

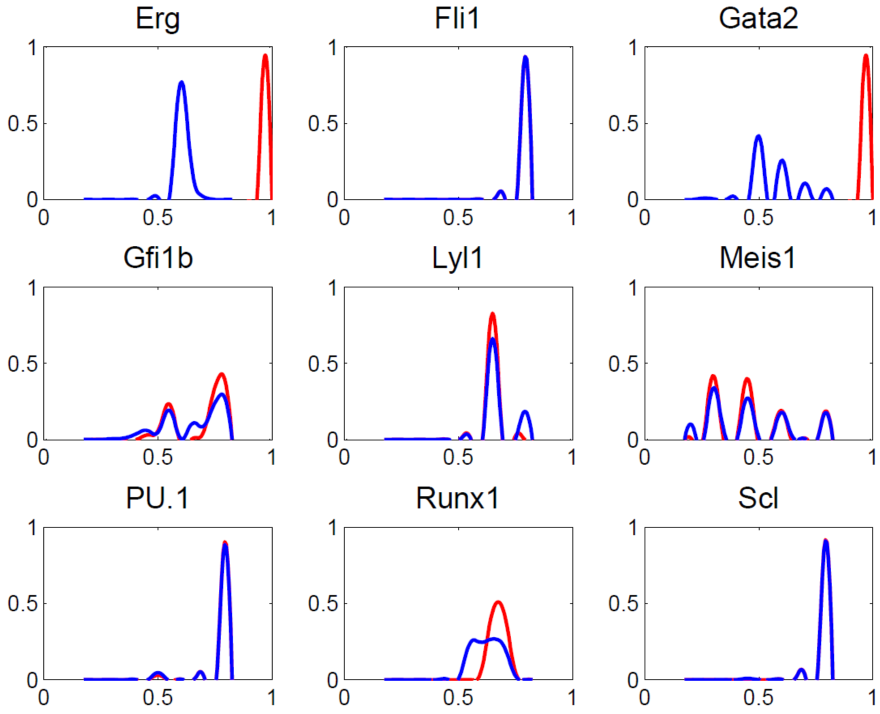
Erg
Fli1
Gata2
Gfi1b
Lyl1
Meis1
PU.1
Runx1
Scl

Top

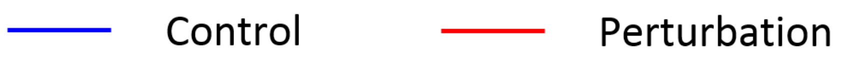
Control
Perturbation

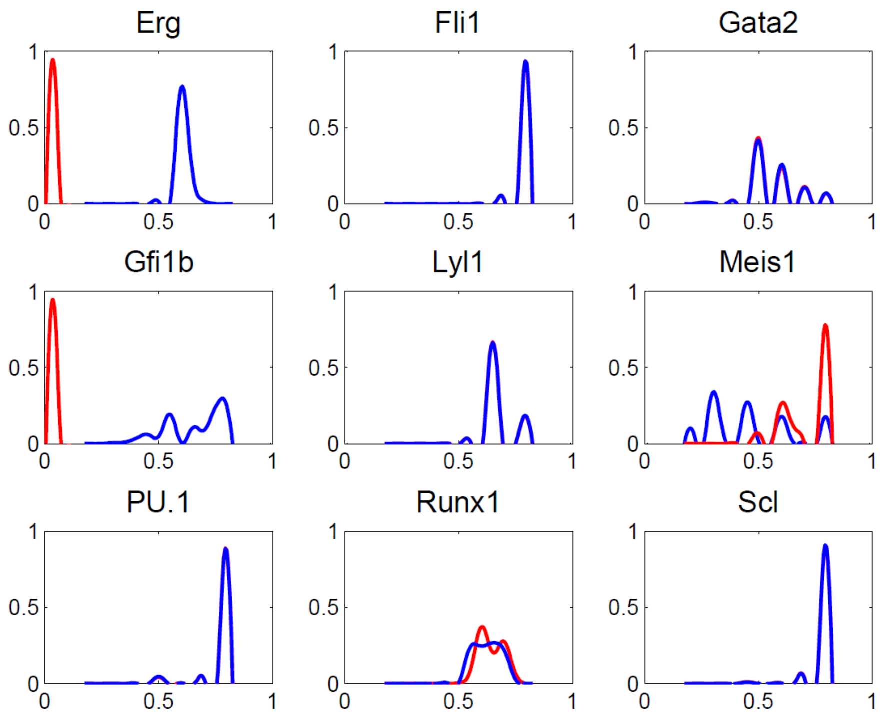
Erg
Fli1
Gata2
Gfi1b
Lyl1
Meis1
PU.1
Runx1
Scl

Top

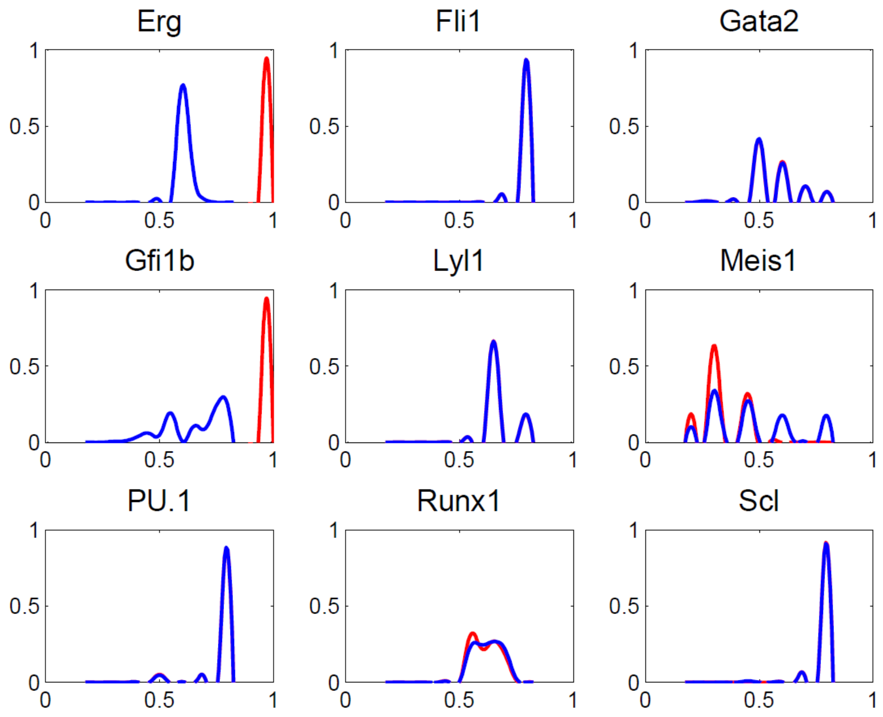
Erg
Fli1
Gata2
Gfi1b
Lyl1
Meis1
PU.1
Runx1
Scl

Top

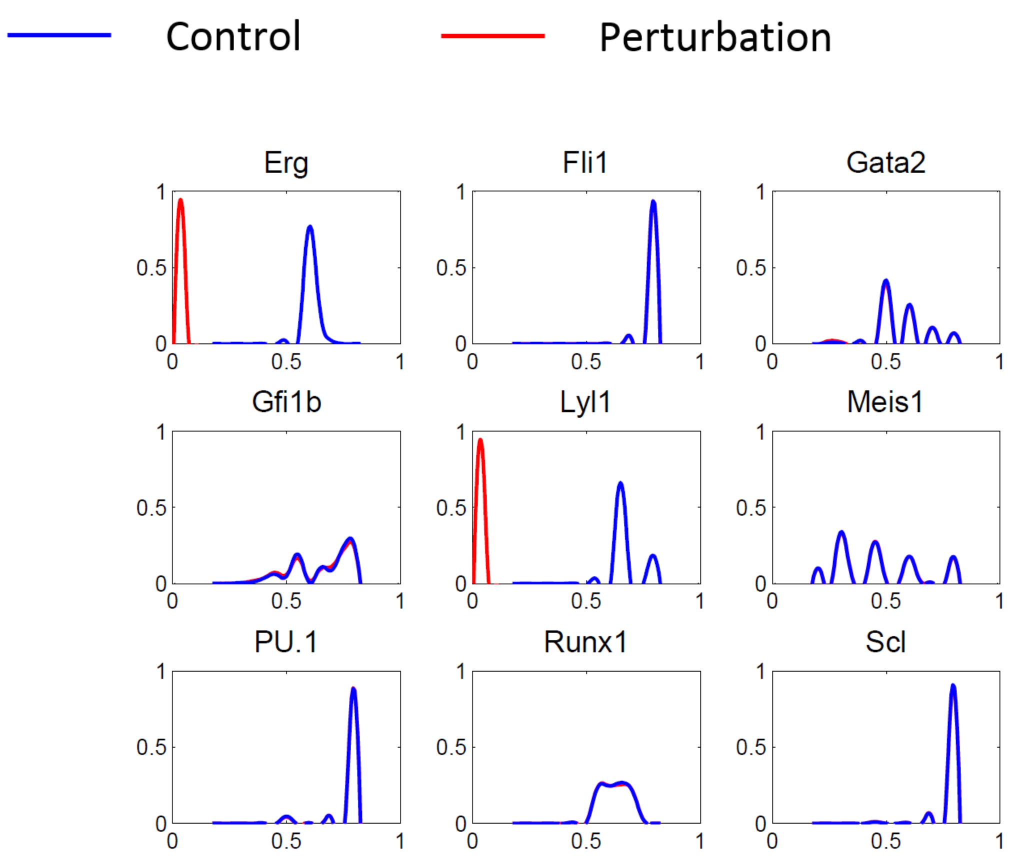
Control
Perturbation
Erg
Fli1
Gata2
Gfi1b
Lyl1
Meis1
PU.1
Runx1
Scl

Top

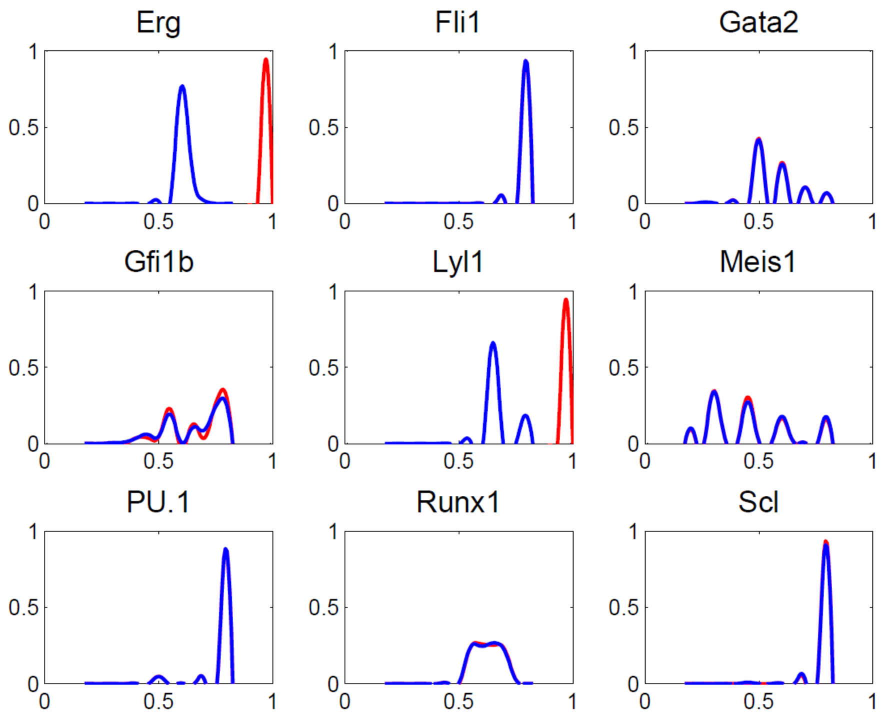
Erg
Fli1
Gata2
Gfi1b
Lyl1
Meis1
PU.1
Runx1
Scl

Top

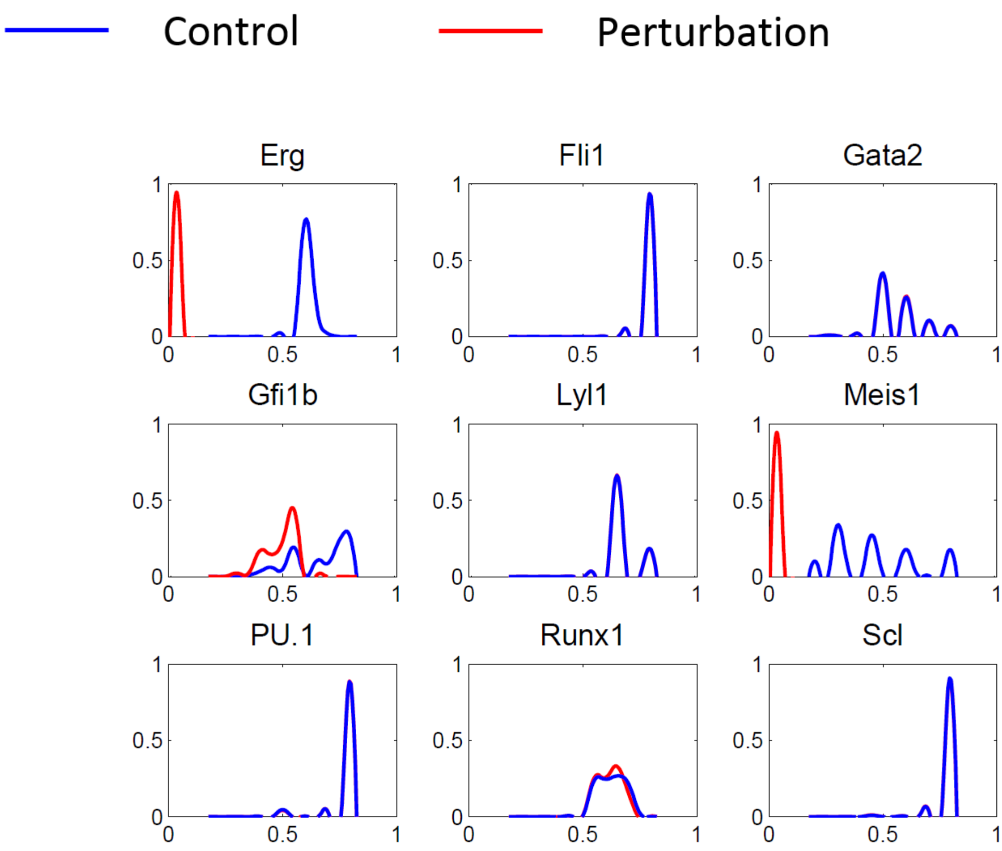
Control
Perturbation
Erg
Fli1
Gata2
Gfi1b
Lyl1
Meis1
PU.1
Runx1
Scl

Top

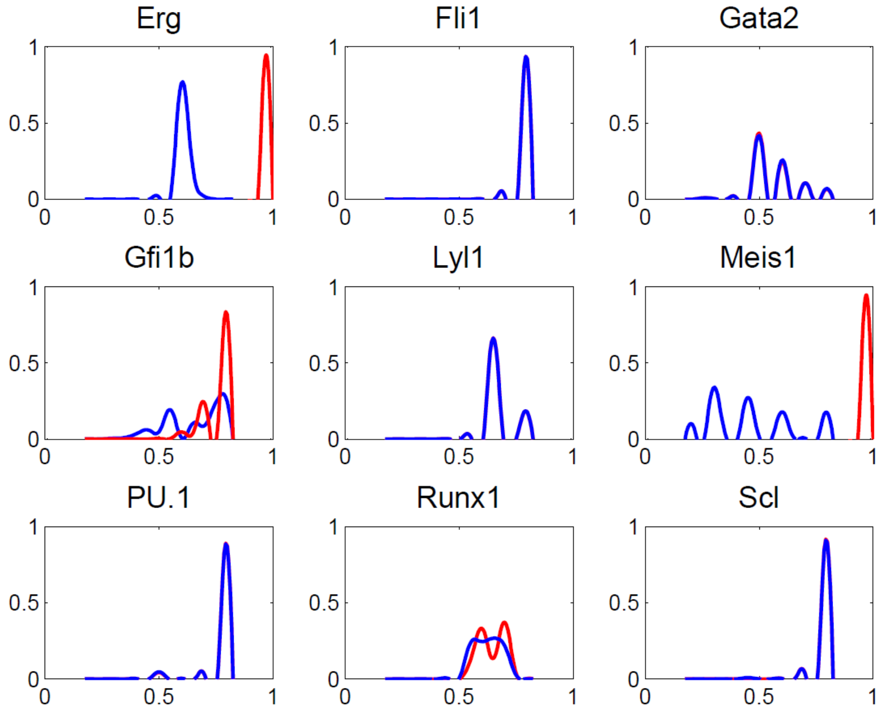
Erg
Fli1
Gata2
Gfi1b
Lyl1
Meis1
PU.1
Runx1
Scl

Top

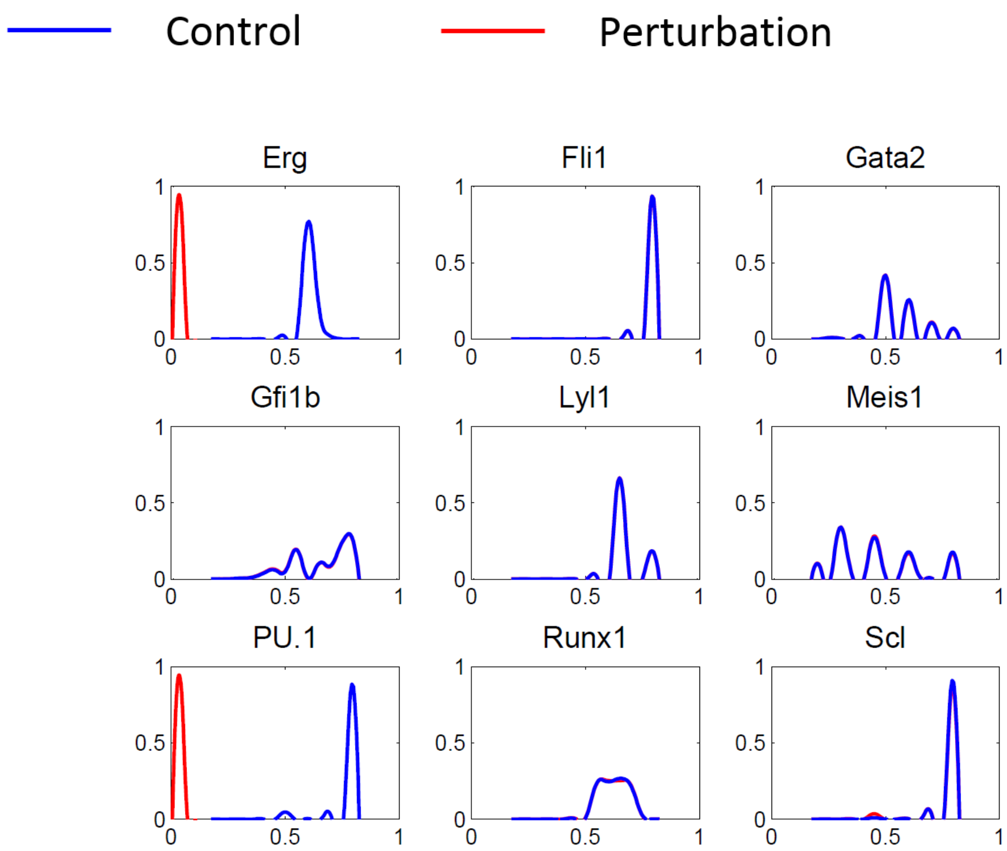
Control
Perturbation
Erg
Fli1
Gata2
Gfi1b
Lyl1
Meis1
PU.1
Runx1
Scl

Top

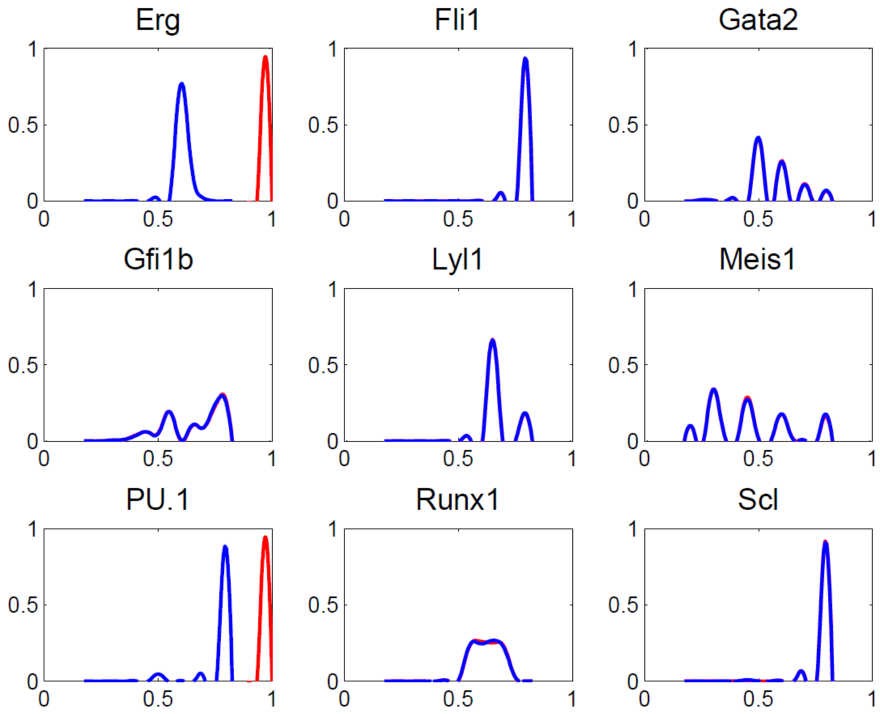
Erg
Fli1
Gata2
Gfi1b
Lyl1
Meis1
PU.1
Runx1
Scl

Top

Control Perturbation

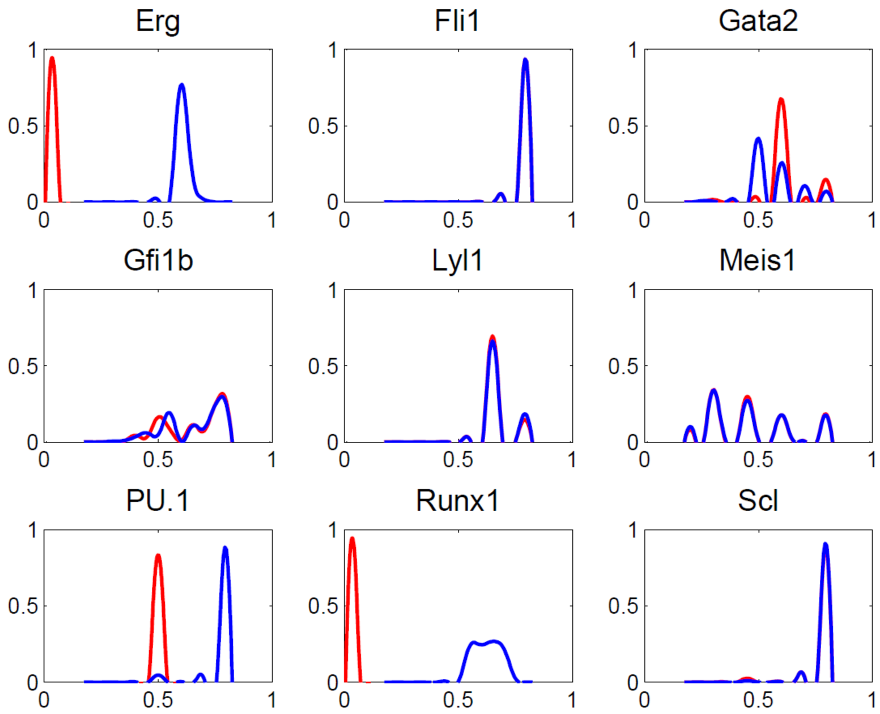

Top

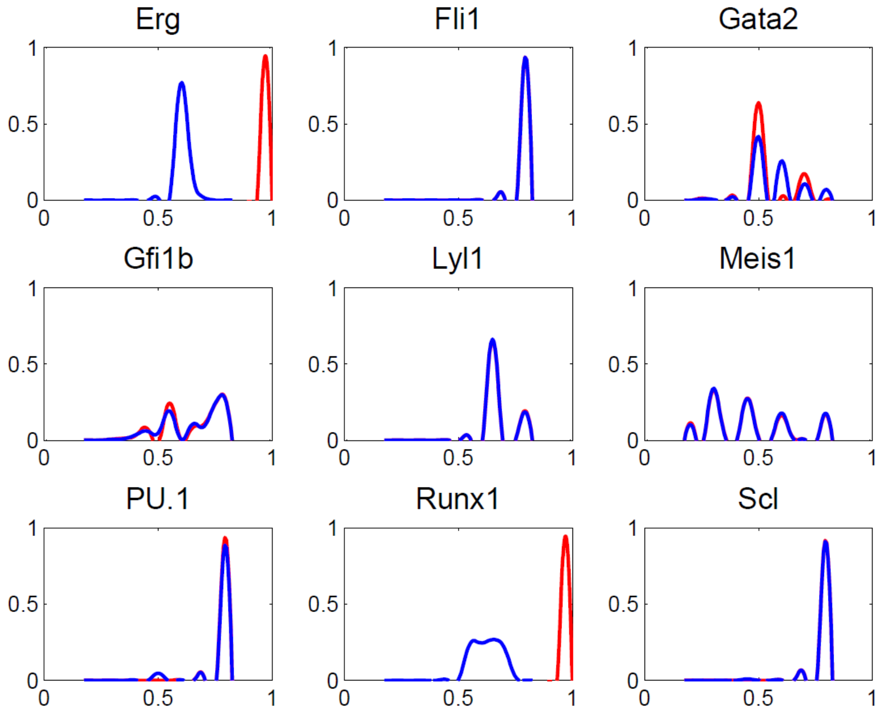

Top

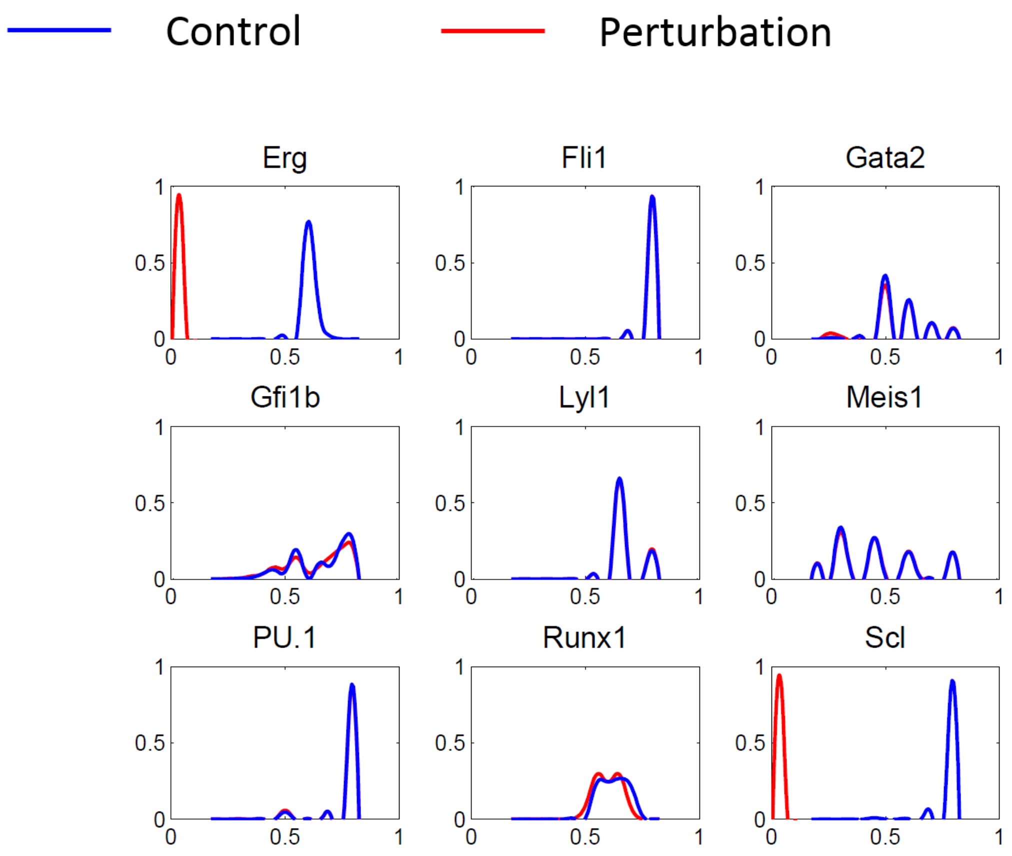
Control
Perturbation
Erg
Fli1
Gata2
Gfi1b
Lyl1
Meis1
PU.1
Runx1
Scl

Top

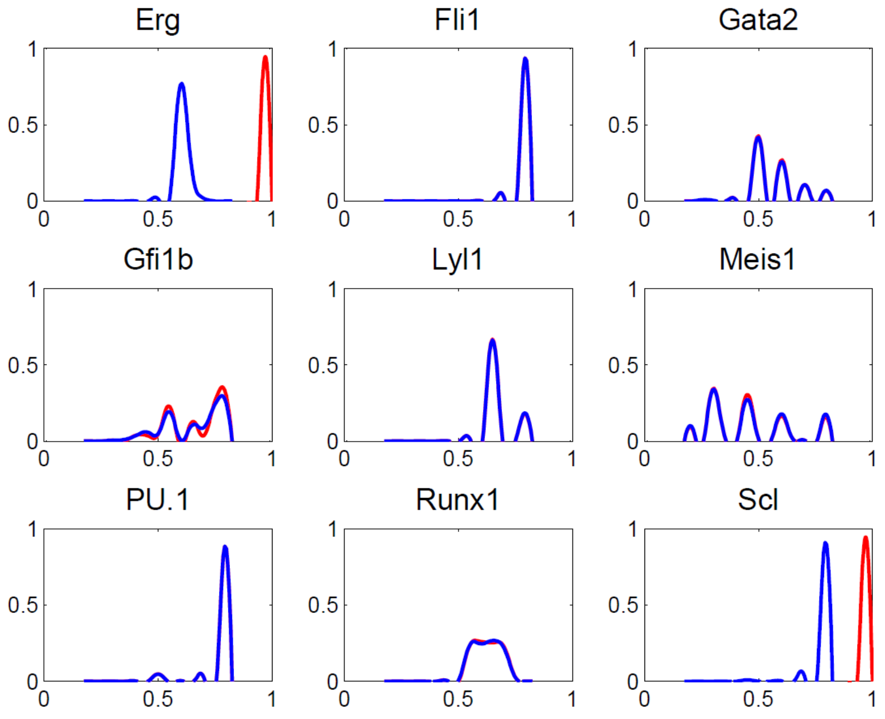
Erg
Fli1
Gata2
Gfi1b
Lyl1
Meis1
PU.1
Runx1
Scl

Top

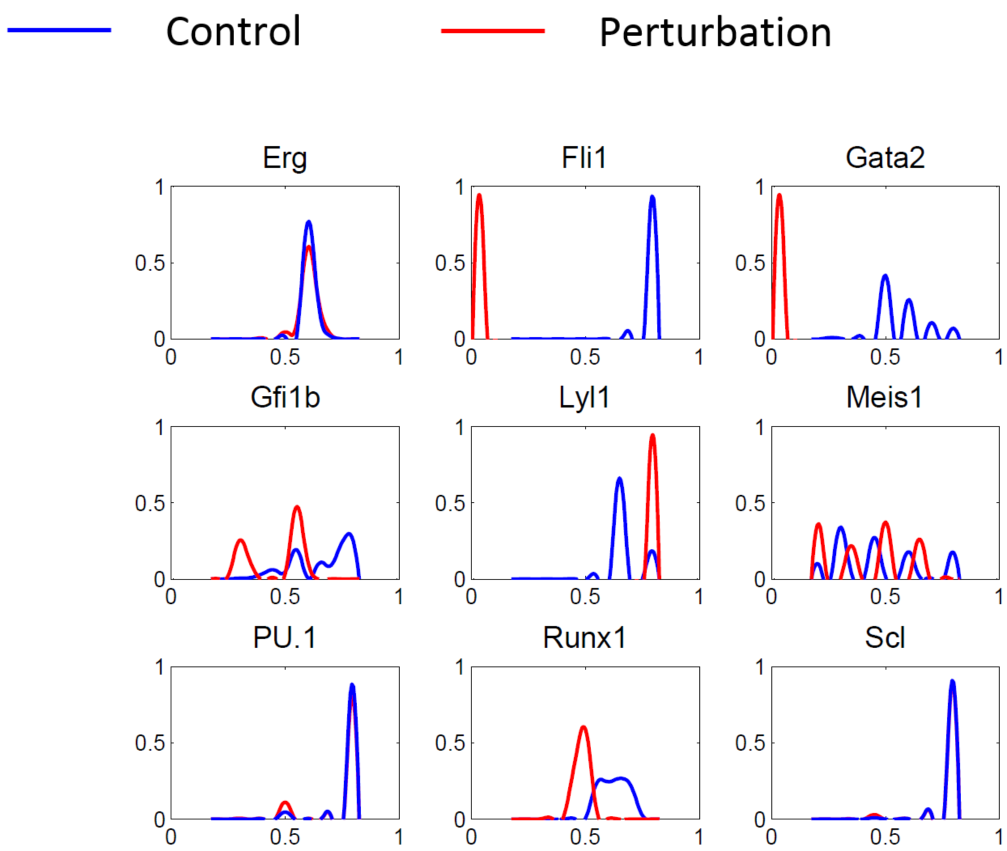
Control
Perturbation
Erg
Fli1
Gata2
Gfi1b
Lyl1
Meis1
PU.1
Runx1
Scl

Top

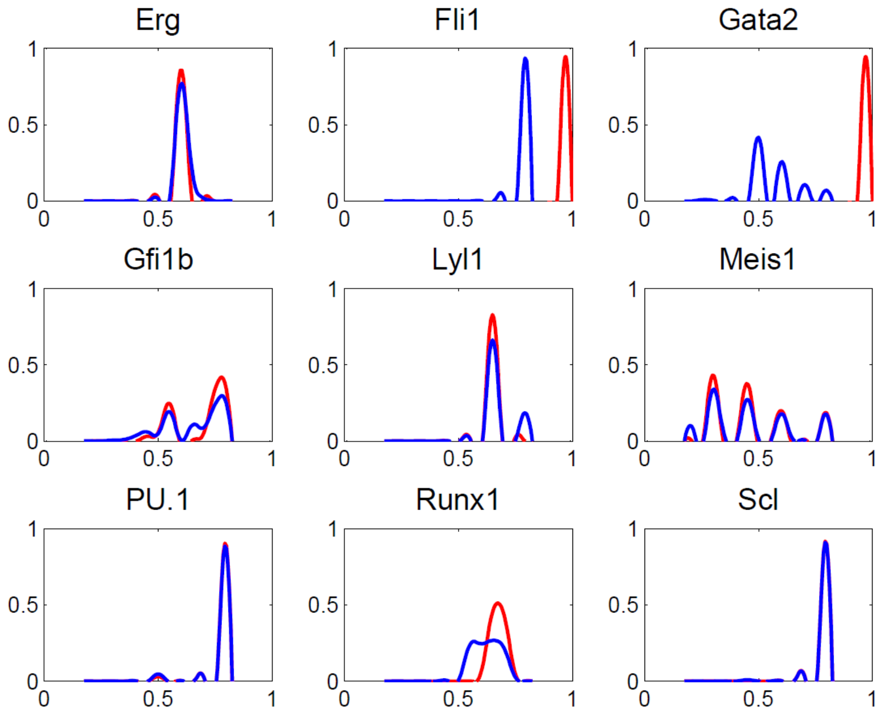
Erg
Fli1
Gata2
Gfi1b
Lyl1
Meis1
PU.1
Runx1
Scl

Top

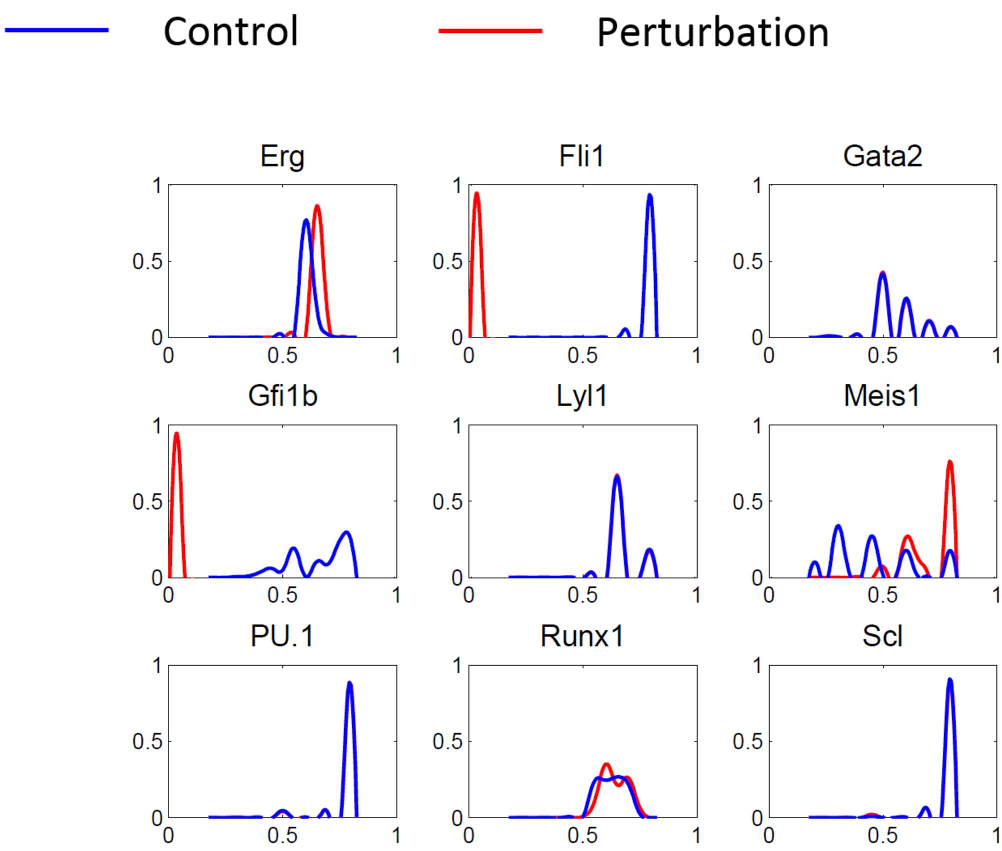
Control
Perturbation
Erg
Fli1
Gata2
Gfi1b
Lyl1
Meis1
PU.1
Runx1
Scl
0
0.5
1

Top

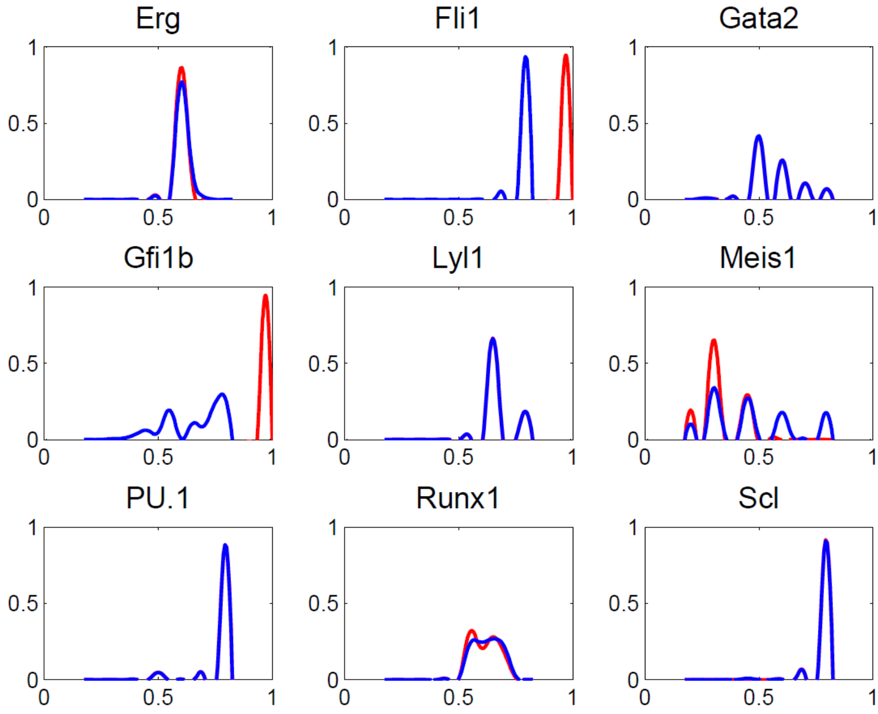
Erg
Fli1
Gata2
Gfi1b
Lyl1
Meis1
PU.1
Runx1
Scl
0
0.5
1

Top

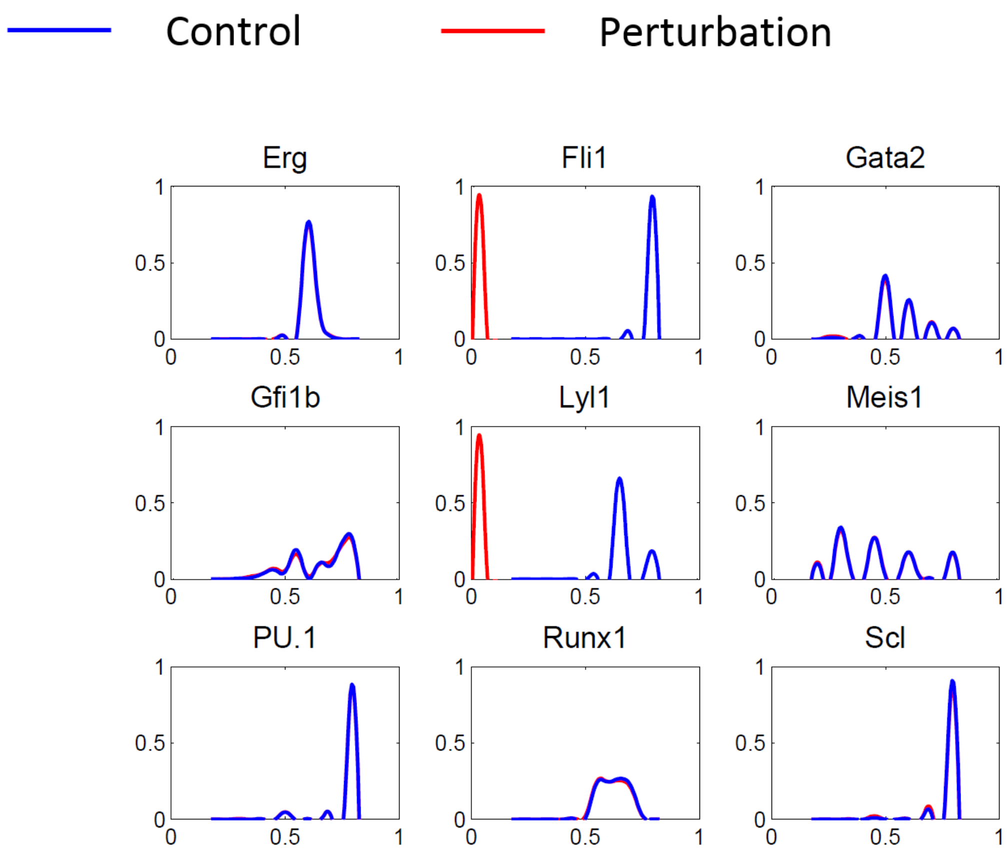
Control
Perturbation
Erg
Fli1
Gata2
Gfi1b
Lyl1
Meis1
PU.1
Runx1
Scl
0
0.5
1

Top

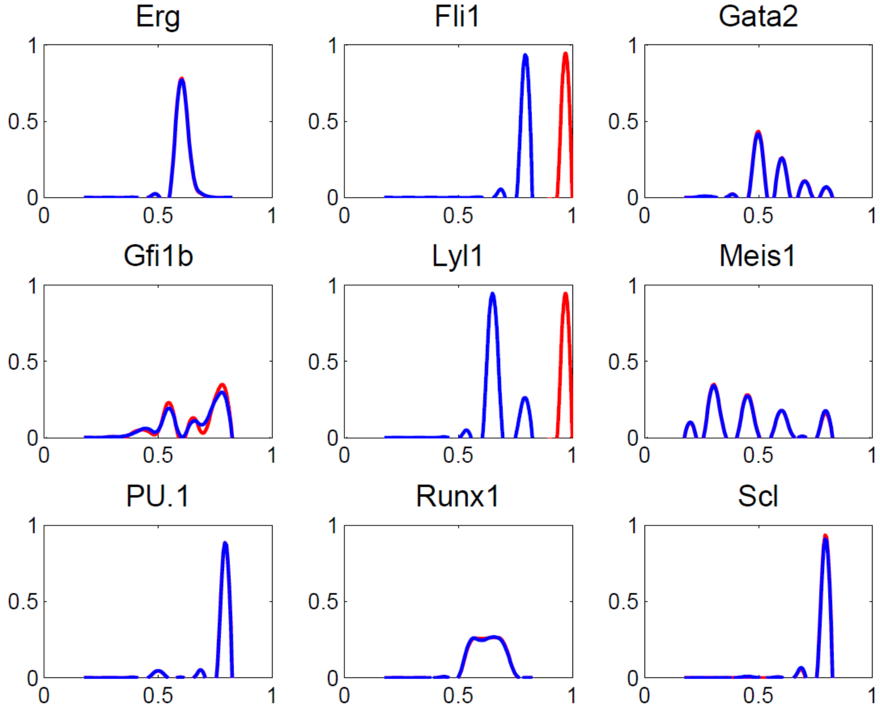
Erg
Fli1
Gata2
Gfi1b
Lyl1
Meis1
PU.1
Runx1
Scl
0
0.5
1

Top

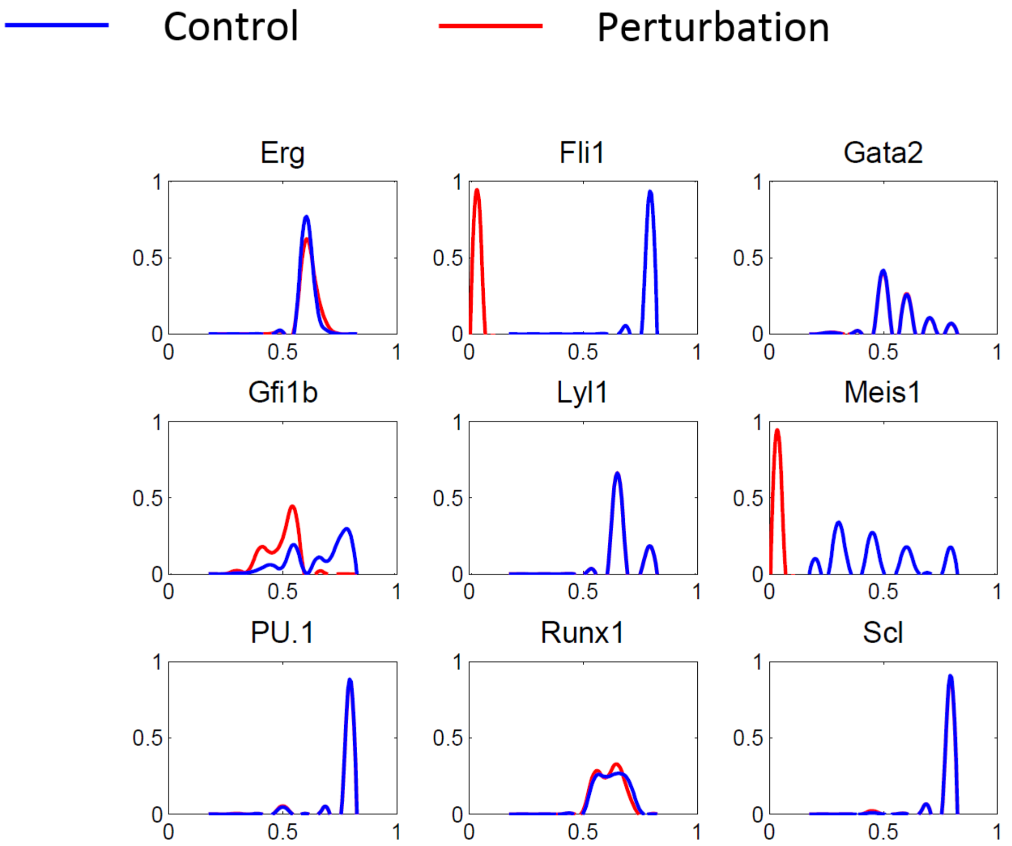
Control
Perturbation
Erg
Fli1
Gata2
Gfi1b
Lyl1
Meis1
PU.1
Runx1
Scl
0
0.5
1

Top

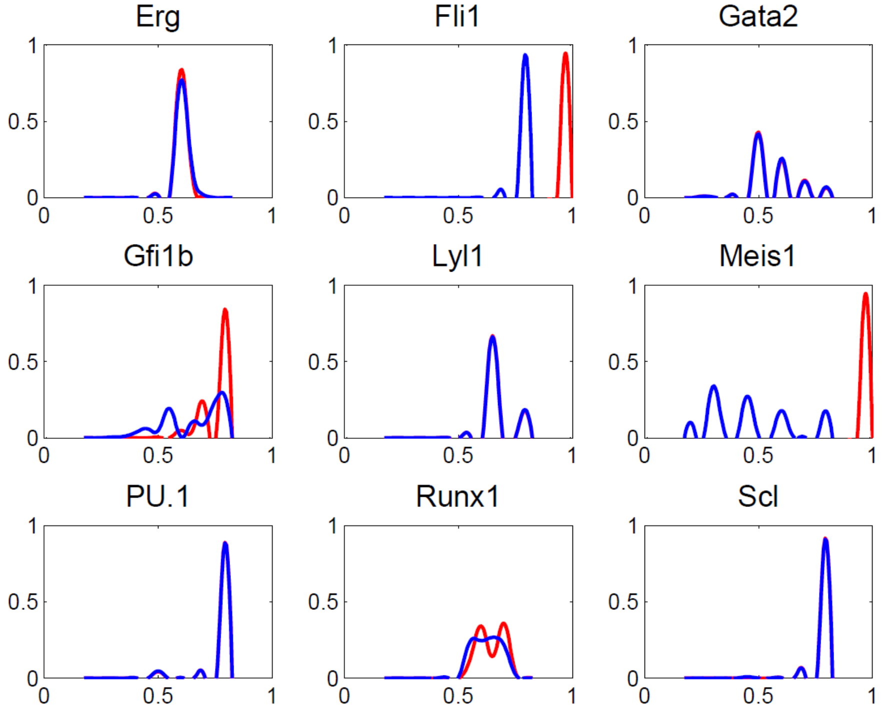
Erg
Fli1
Gata2
Gfi1b
Lyl1
Meis1
PU.1
Runx1
Scl
0
0.5
1

Top

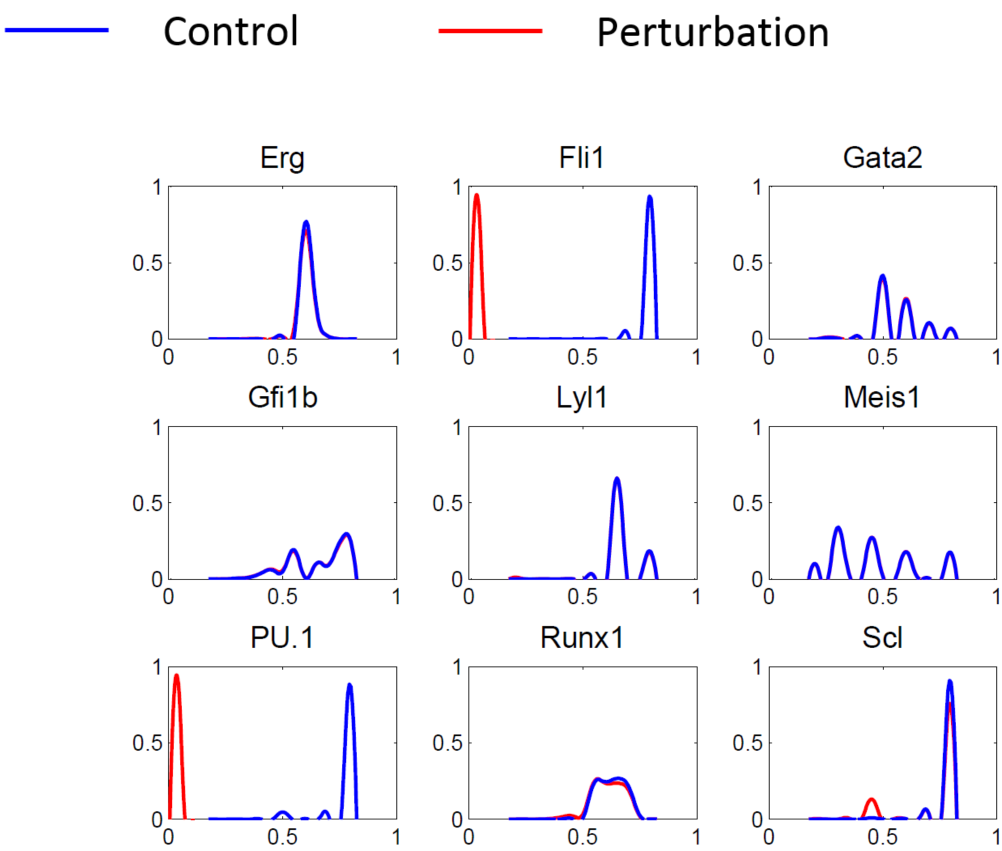
Control
Perturbation
Erg
Fli1
Gata2
Gfi1b
Lyl1
Meis1
PU.1
Runx1
Scl
0
0.5
1

[Top](#)

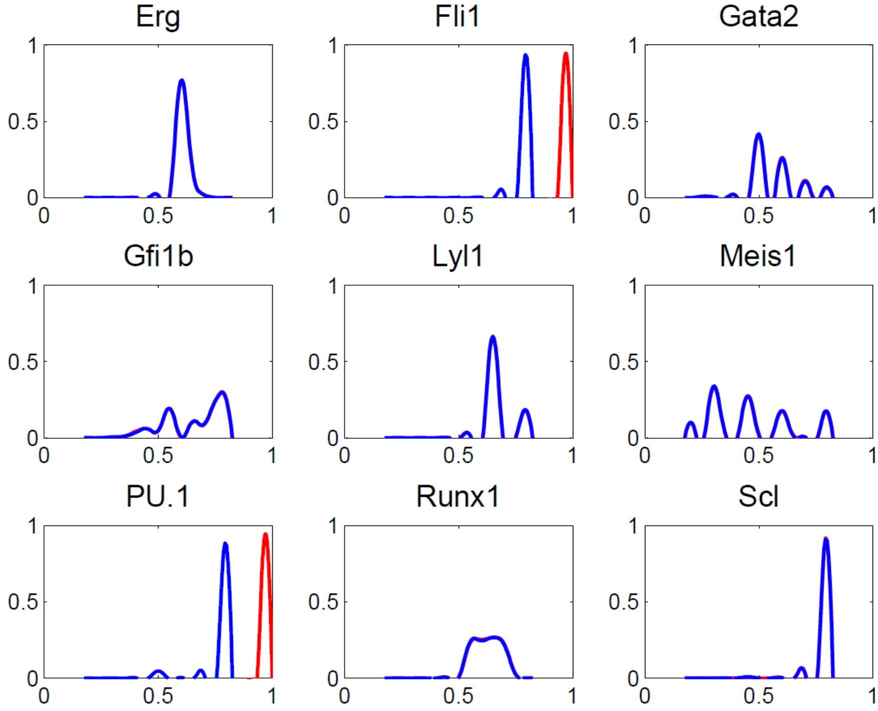
Erg
Fli1
Gata2
Gfi1b
Lyl1
Meis1
PU.1
Runx1
Scl
0
0.5
1

[Top](#)

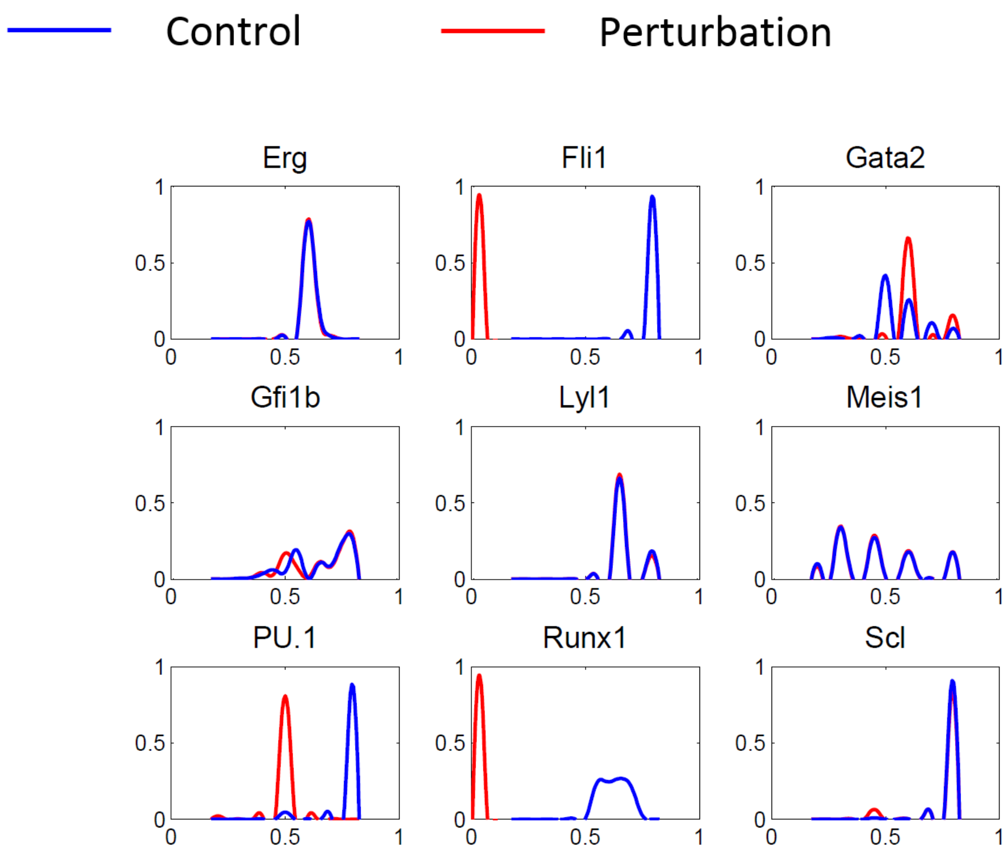
Control
Perturbation
Erg
Fli1
Gata2
Gfi1b
Lyl1
Meis1
PU.1
Runx1
Scl

Top

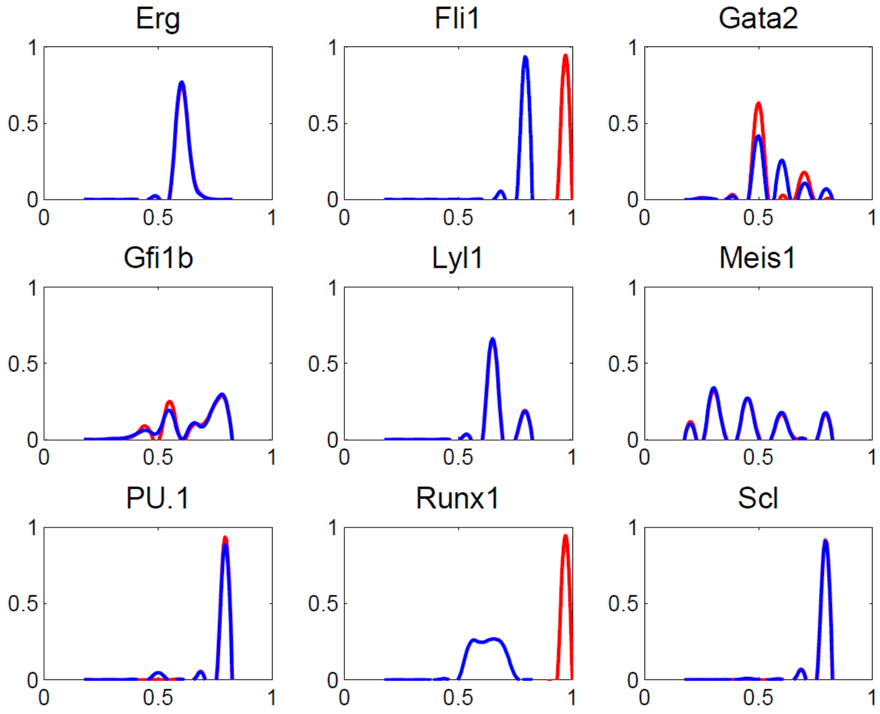
Erg
Fli1
Gata2
Gfi1b
Lyl1
Meis1
PU.1
Runx1
Scl

Top

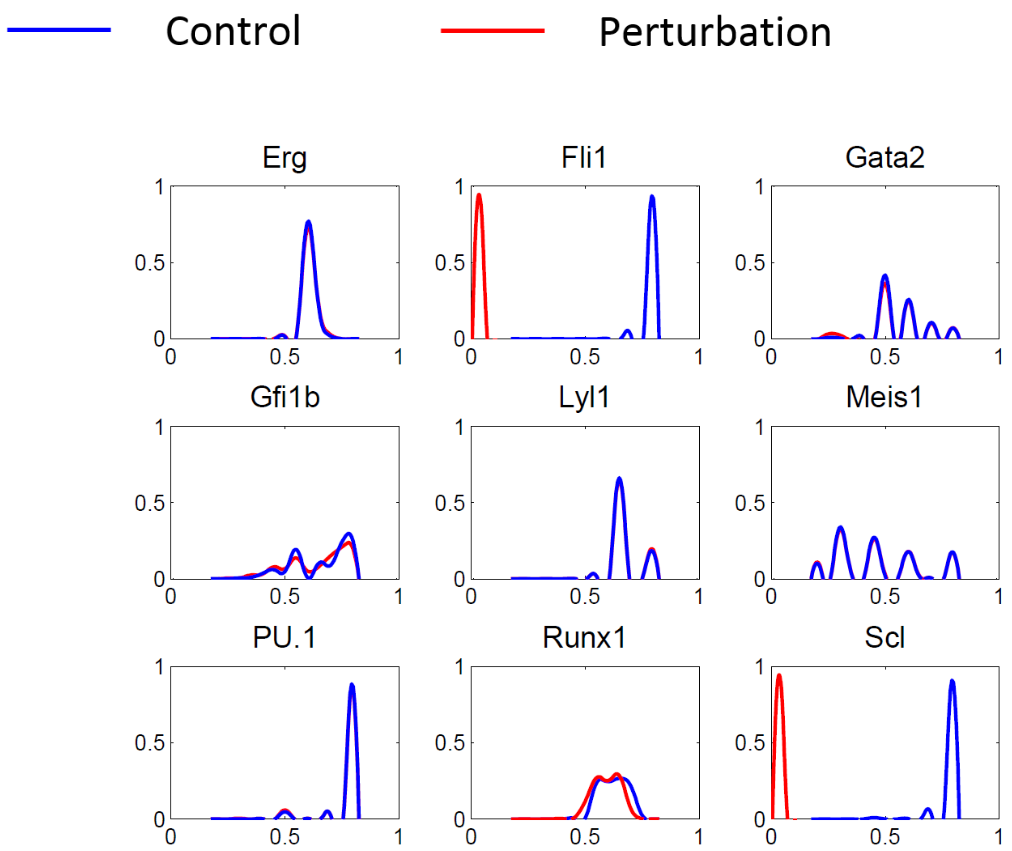
Control
Perturbation
Erg
Fli1
Gata2
Gfi1b
Lyl1
Meis1
PU.1
Runx1
Scl

Top

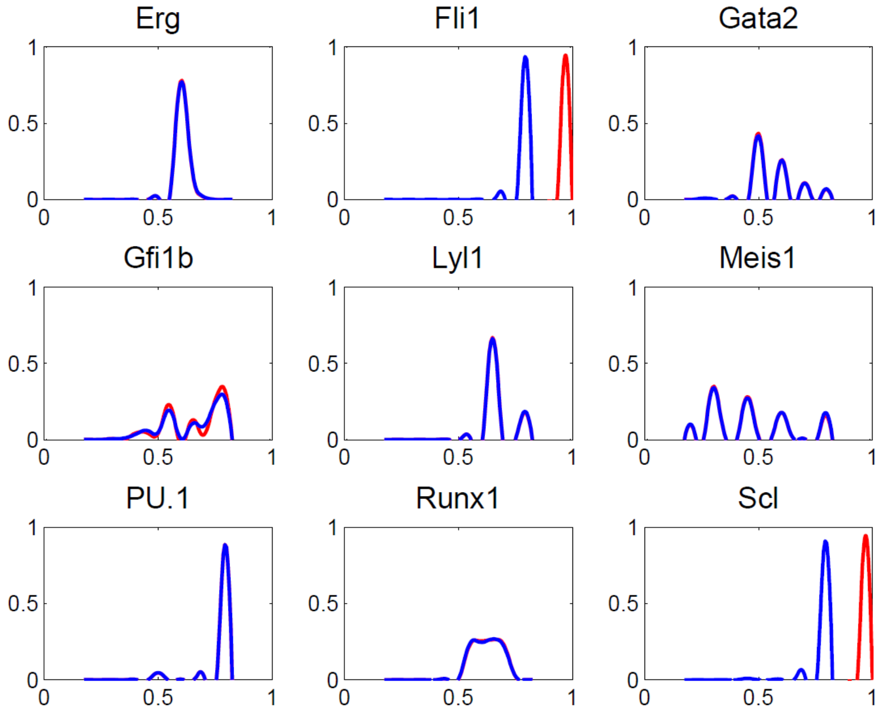
Erg
Fli1
Gata2
Gfi1b
Lyl1
Meis1
PU.1
Runx1
Scl

Top

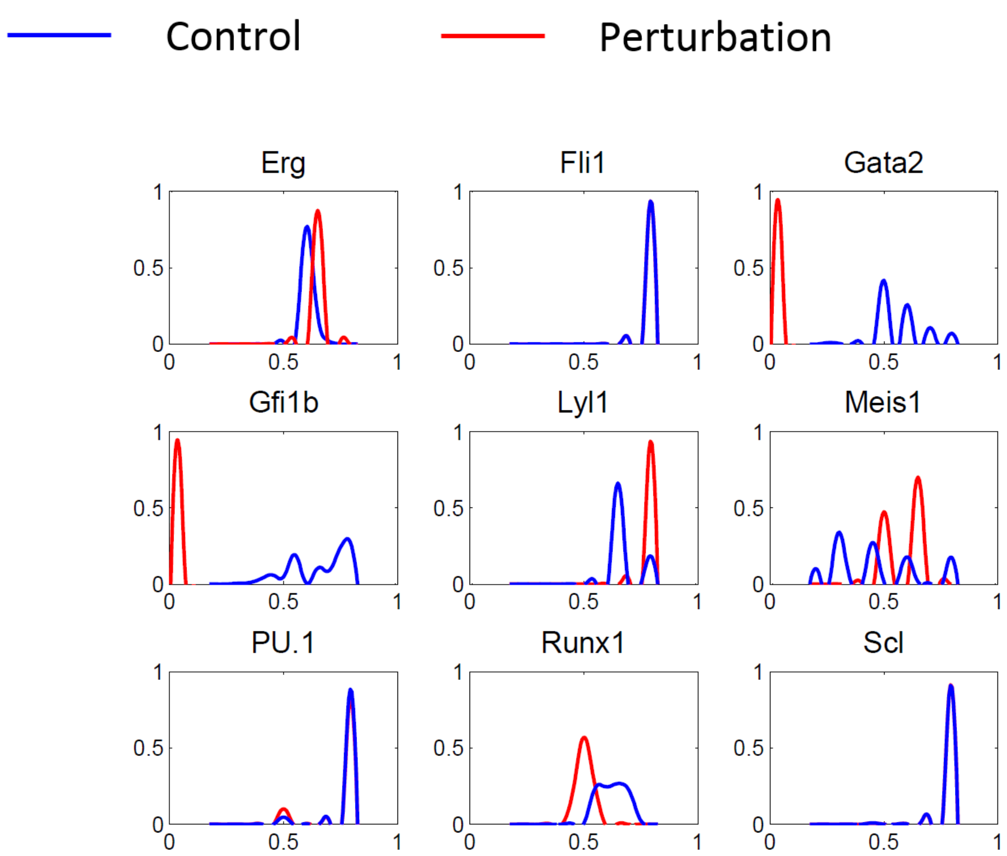
Control
Perturbation
Erg
Fli1
Gata2
Gfi1b
Lyl1
Meis1
PU.1
Runx1
Scl

Top

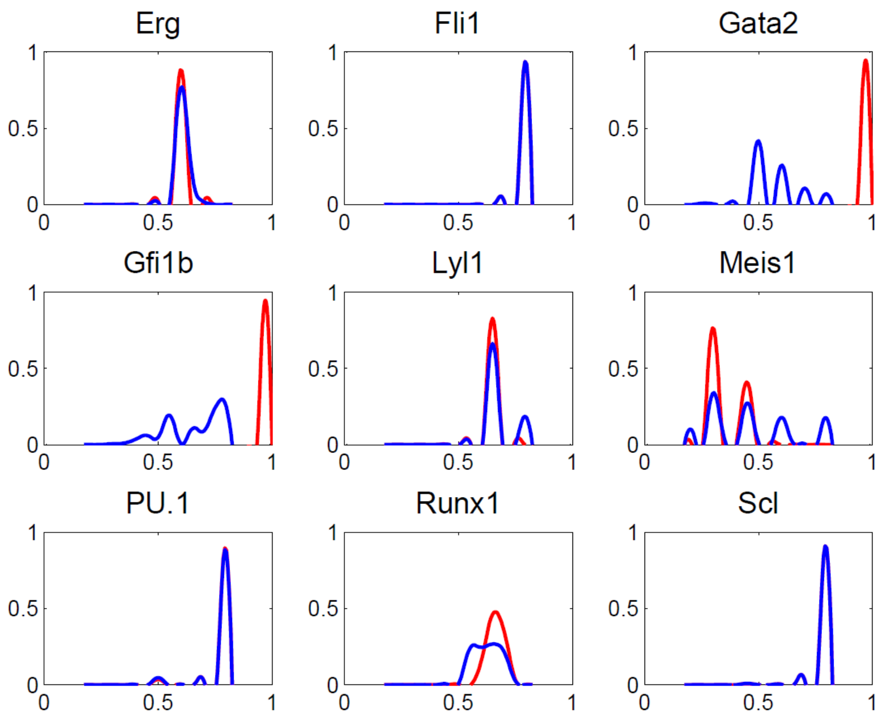
Erg
Fli1
Gata2
Gfi1b
Lyl1
Meis1
PU.1
Runx1
Scl

Top

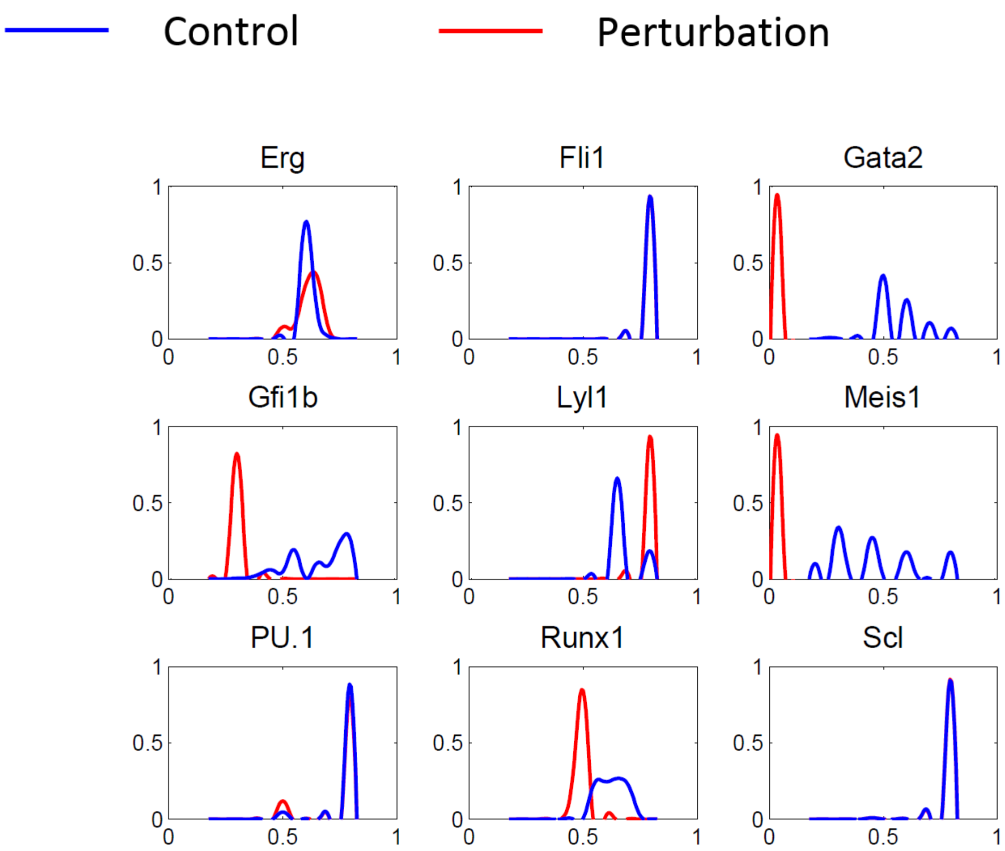
Control
Perturbation
Erg
Fli1
Gata2
Gfi1b
Lyl1
Meis1
PU.1
Runx1
Scl
0
0.5
1

Top

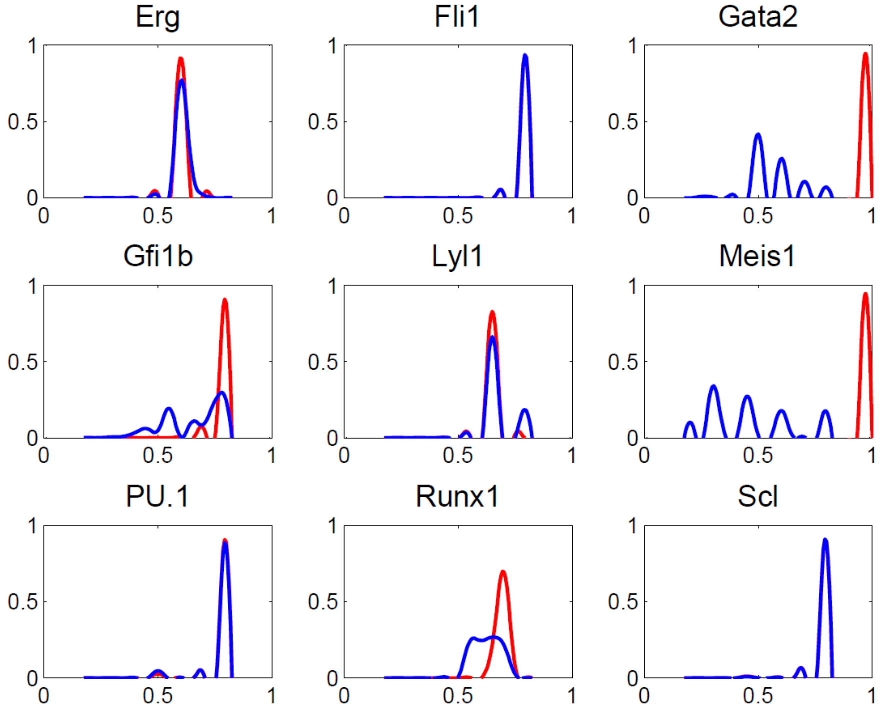
Erg
Fli1
Gata2
Gfi1b
Lyl1
Meis1
PU.1
Runx1
Scl
0
0.5
1

Top

Control Perturbation

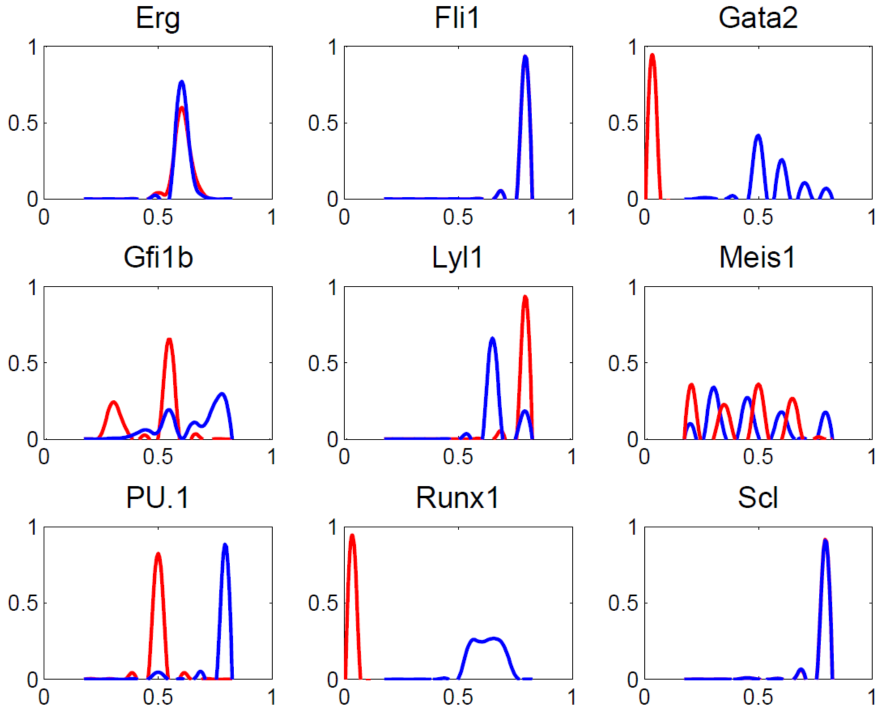

Top

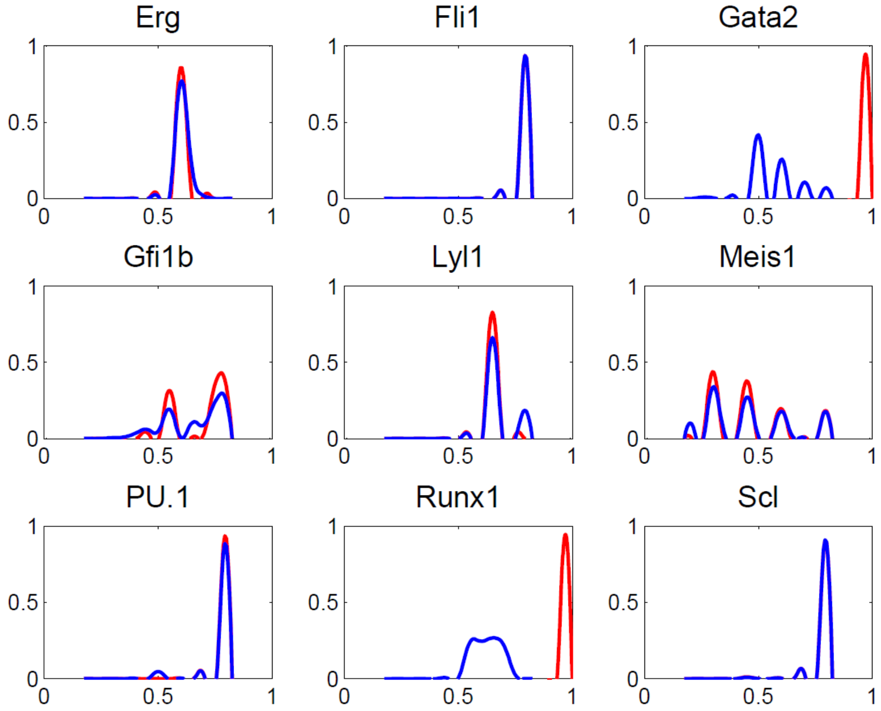

Top

Control Perturbation

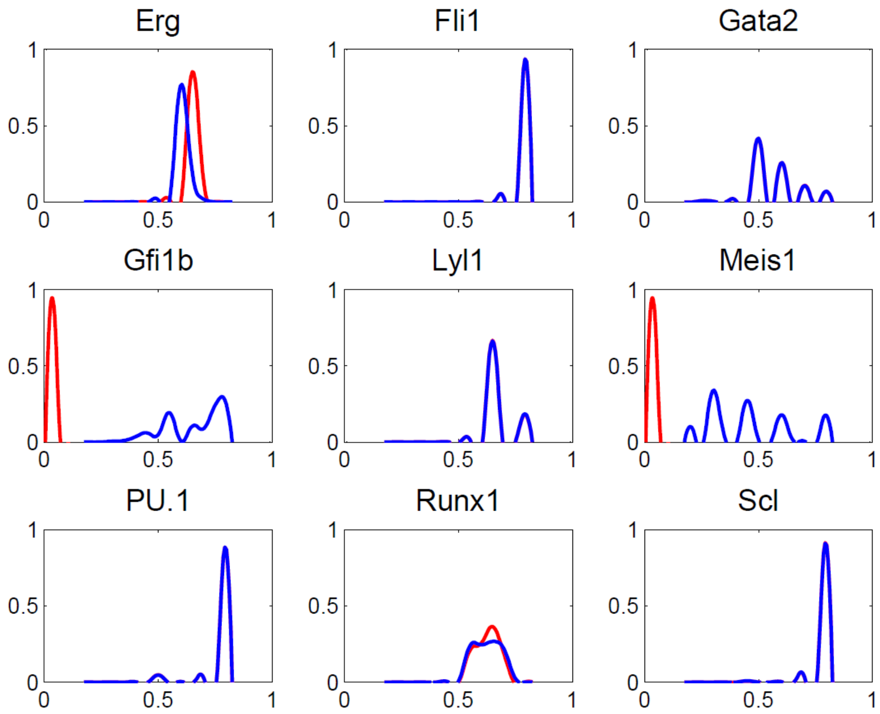

Top

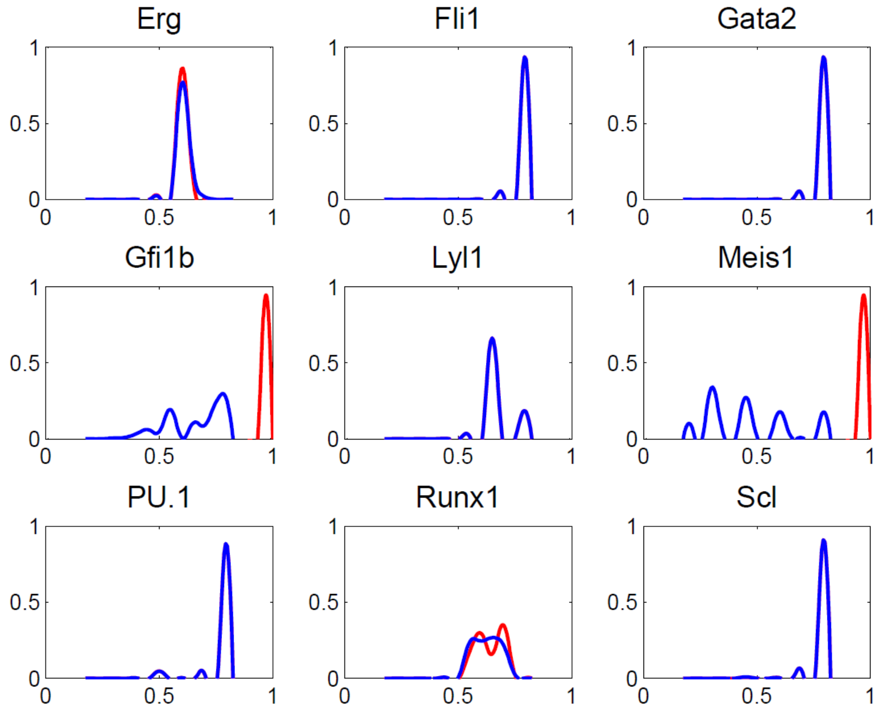

Top

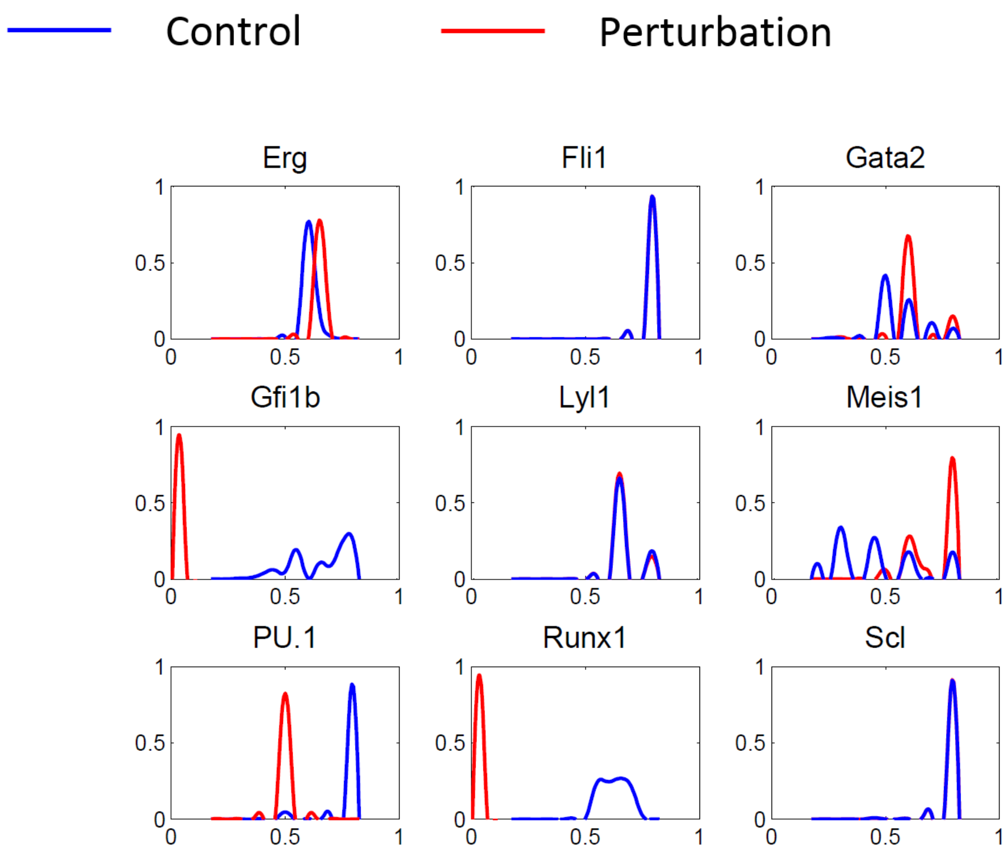

Control
Perturbation
Erg
Fli1
Gata2
Gfi1b
Lyl1
Meis1
PU.1
Runx1
Scl

Top

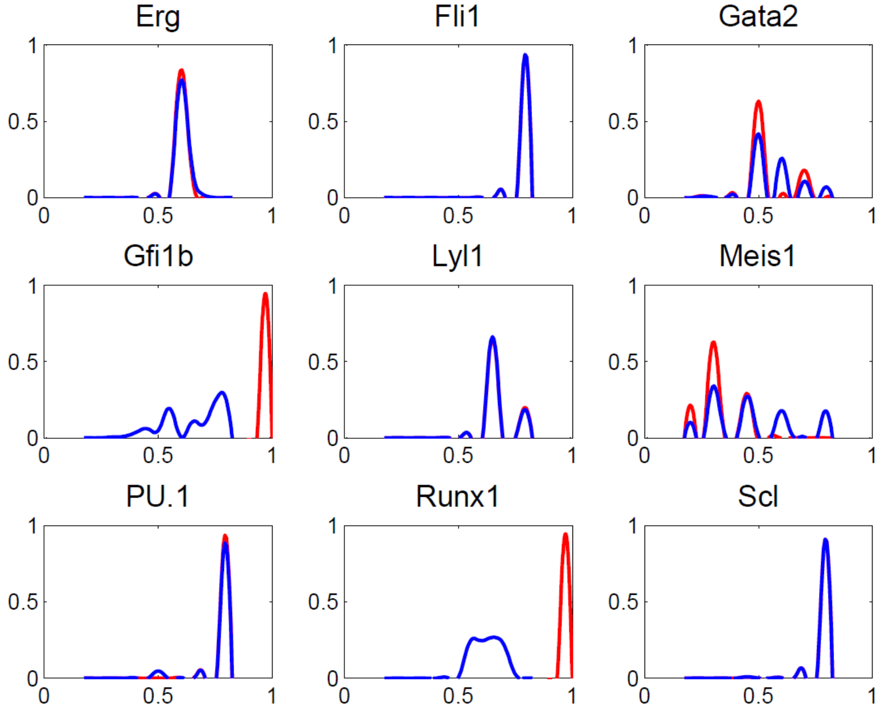

Erg
Fli1
Gata2
Gfi1b
Lyl1
Meis1
PU.1
Runx1
Scl

Top

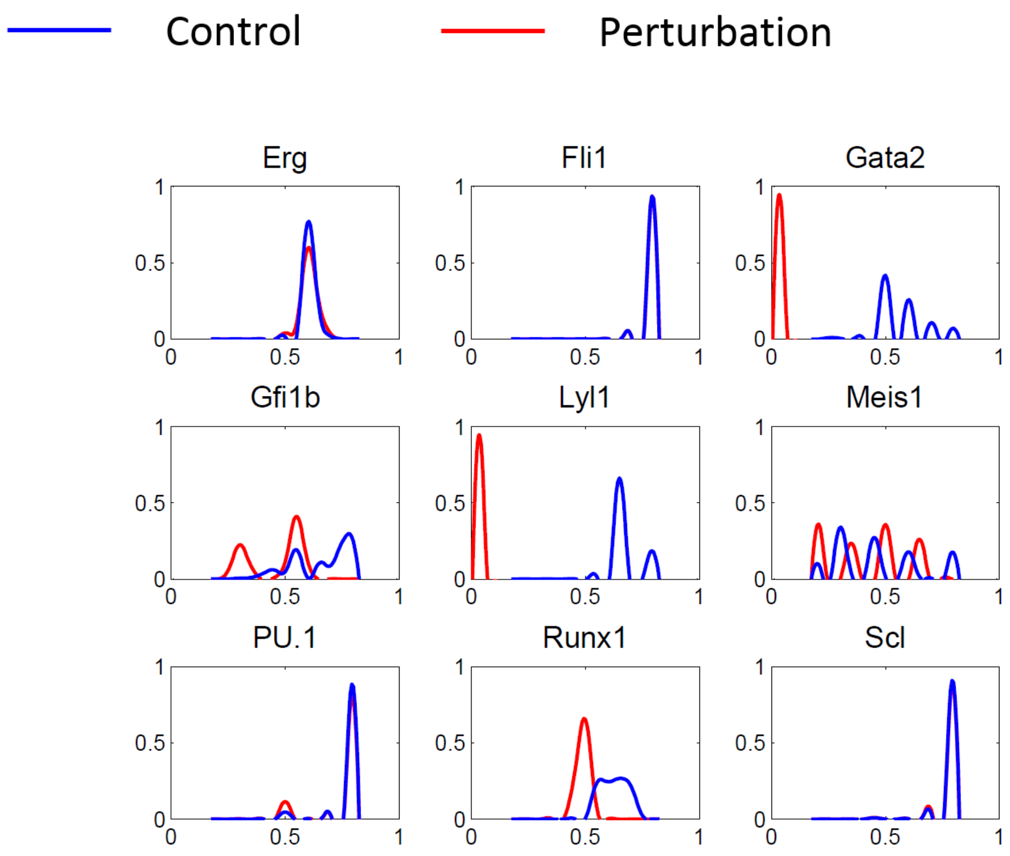
Control
Perturbation
Erg
Fli1
Gata2
Gfi1b
Lyl1
Meis1
PU.1
Runx1
Scl

Top

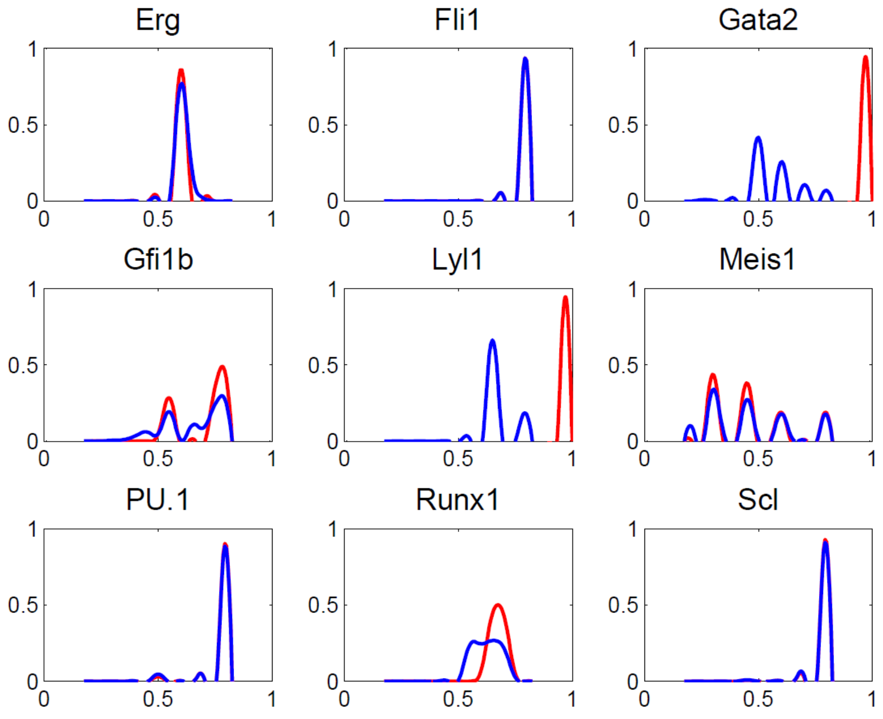
Erg
Fli1
Gata2
Gfi1b
Lyl1
Meis1
PU.1
Runx1
Scl

Top

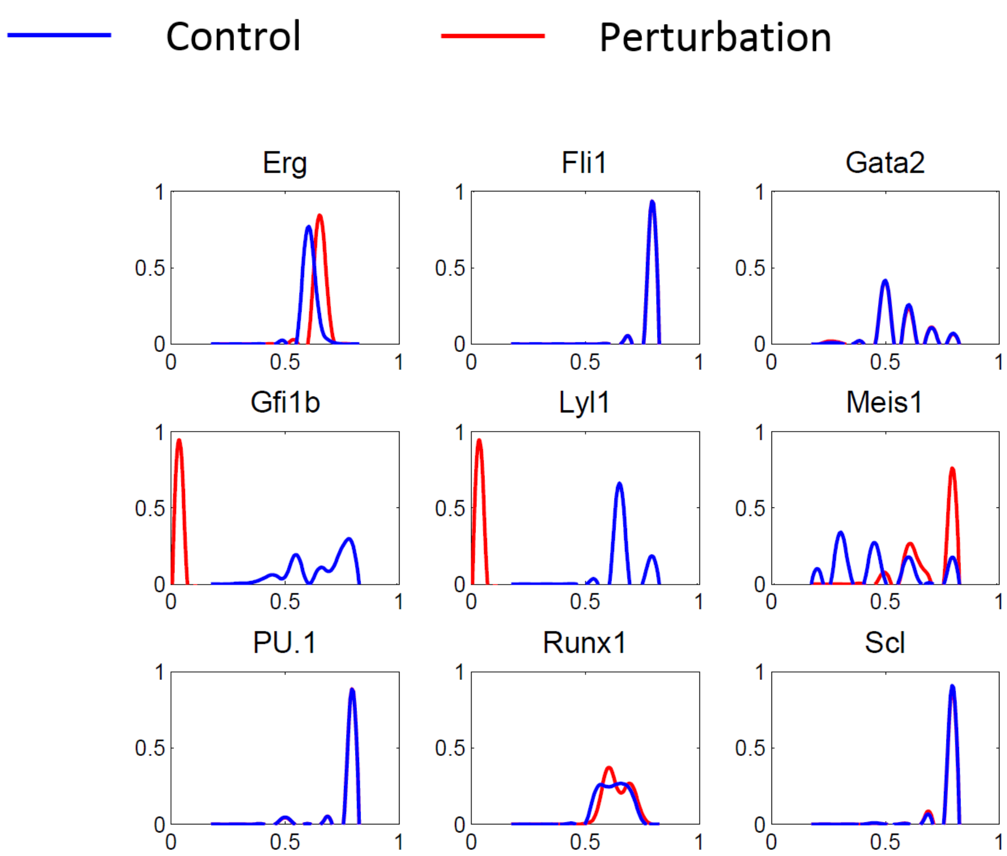
Control
Perturbation
Erg
Fli1
Gata2
Gfi1b
Lyl1
Meis1
PU.1
Runx1
Scl

Top

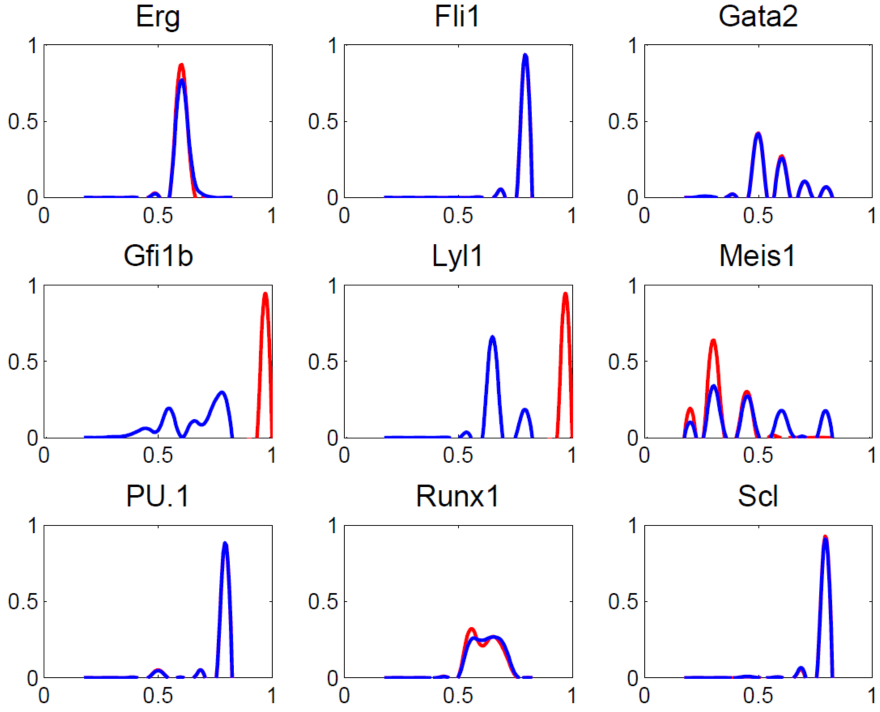
Erg
Fli1
Gata2
Gfi1b
Lyl1
Meis1
PU.1
Runx1
Scl

Top

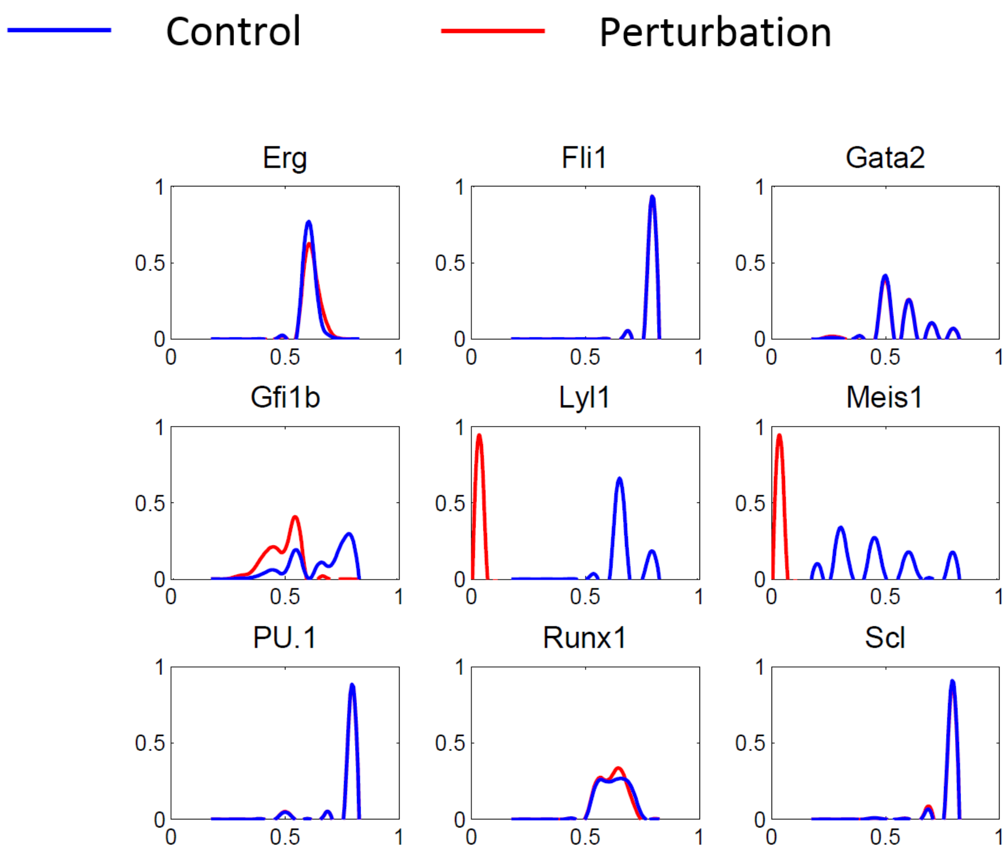
Control
Perturbation
Erg
Fli1
Gata2
Gfi1b
Lyl1
Meis1
PU.1
Runx1
Scl

Top

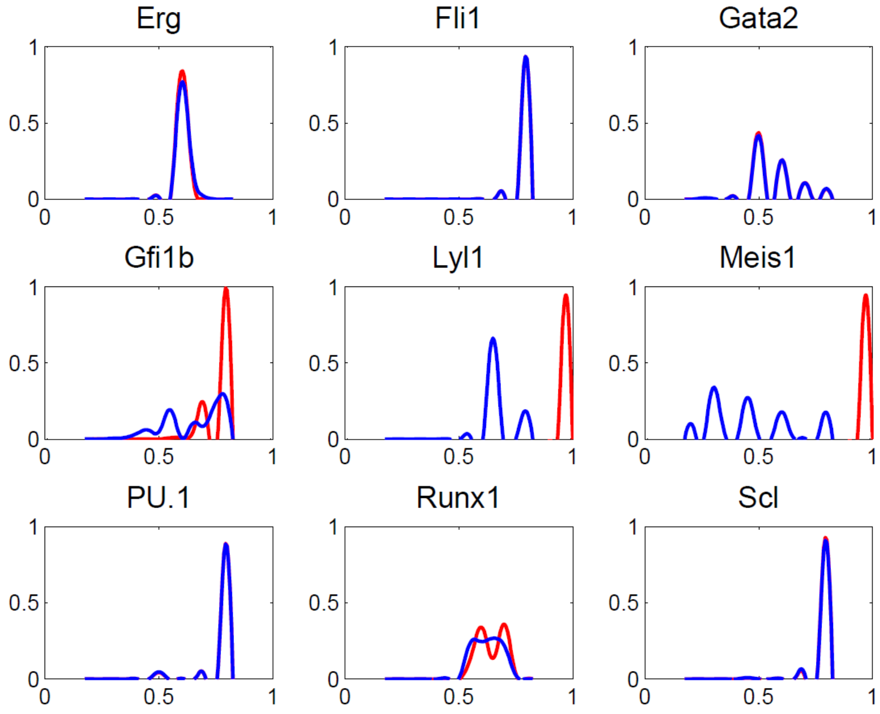
Erg
Fli1
Gata2
Gfi1b
Lyl1
Meis1
PU.1
Runx1
Scl

Top

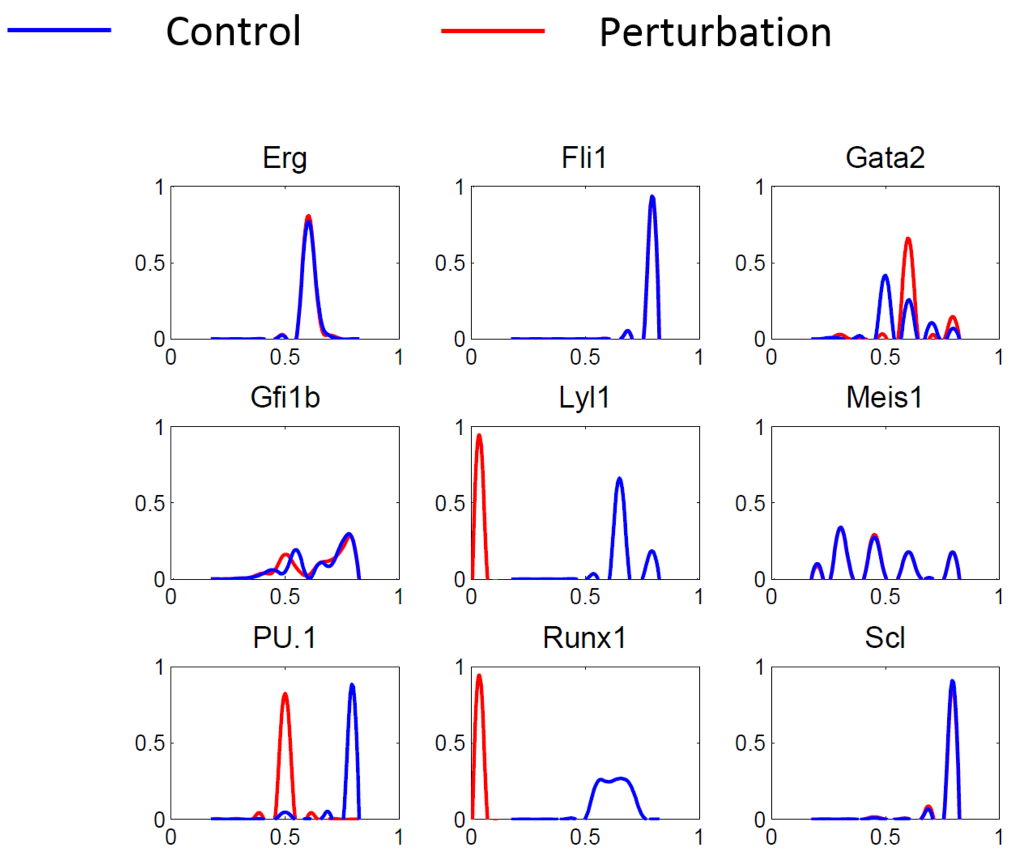

Control
Perturbation
Erg
Fli1
Gata2
Gfi1b
Lyl1
Meis1
PU.1
Runx1
Scl

[Top](#)

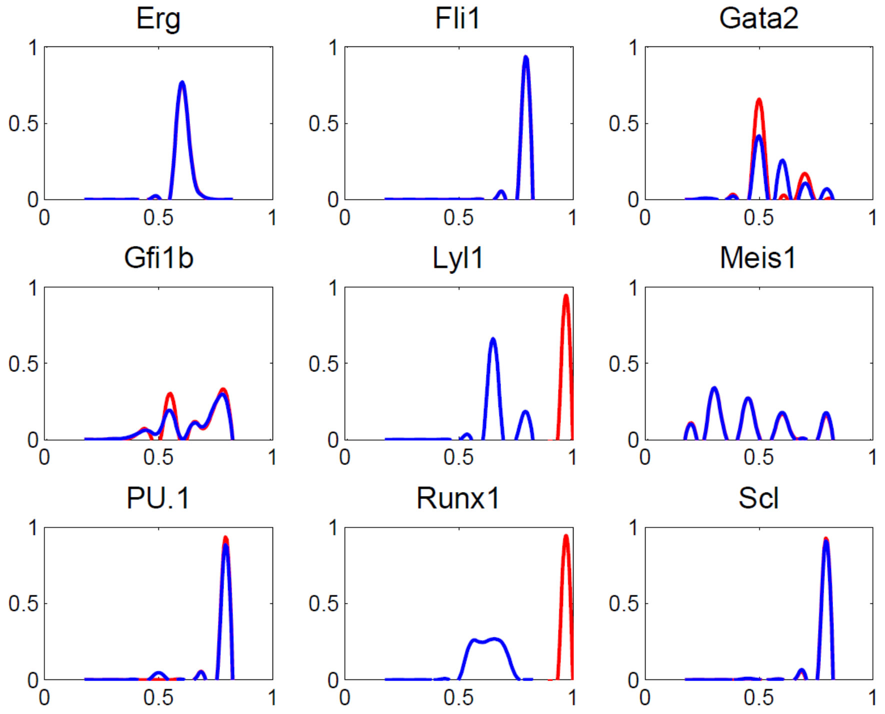

Erg
Fli1
Gata2
Gfi1b
Lyl1
Meis1
PU.1
Runx1
Scl

[Top](#)

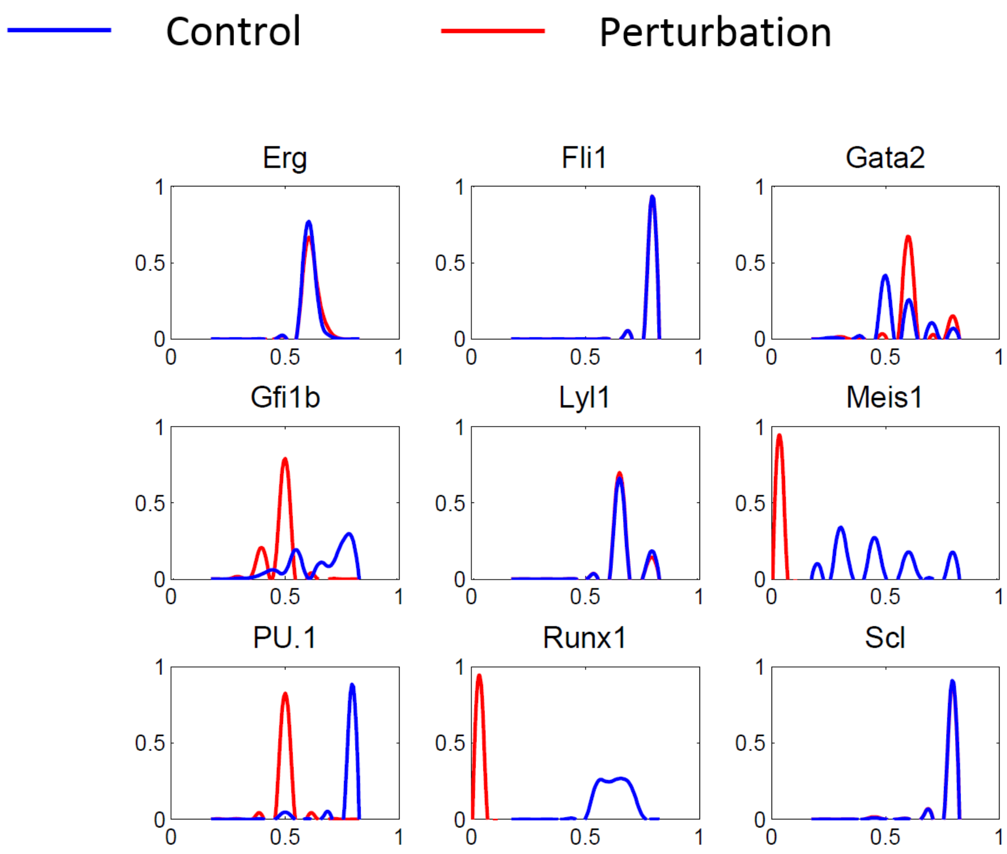
Control
Perturbation
Erg
Fli1
Gata2
Gfi1b
Lyl1
Meis1
PU.1
Runx1
Scl

Top

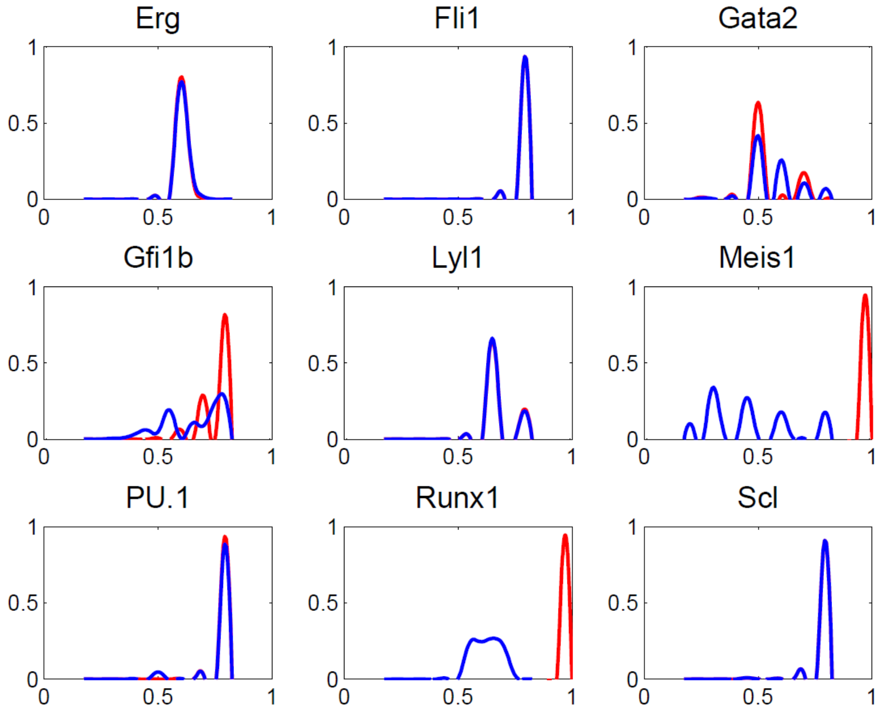
Erg
Fli1
Gata2
Gfi1b
Lyl1
Meis1
PU.1
Runx1
Scl

Top

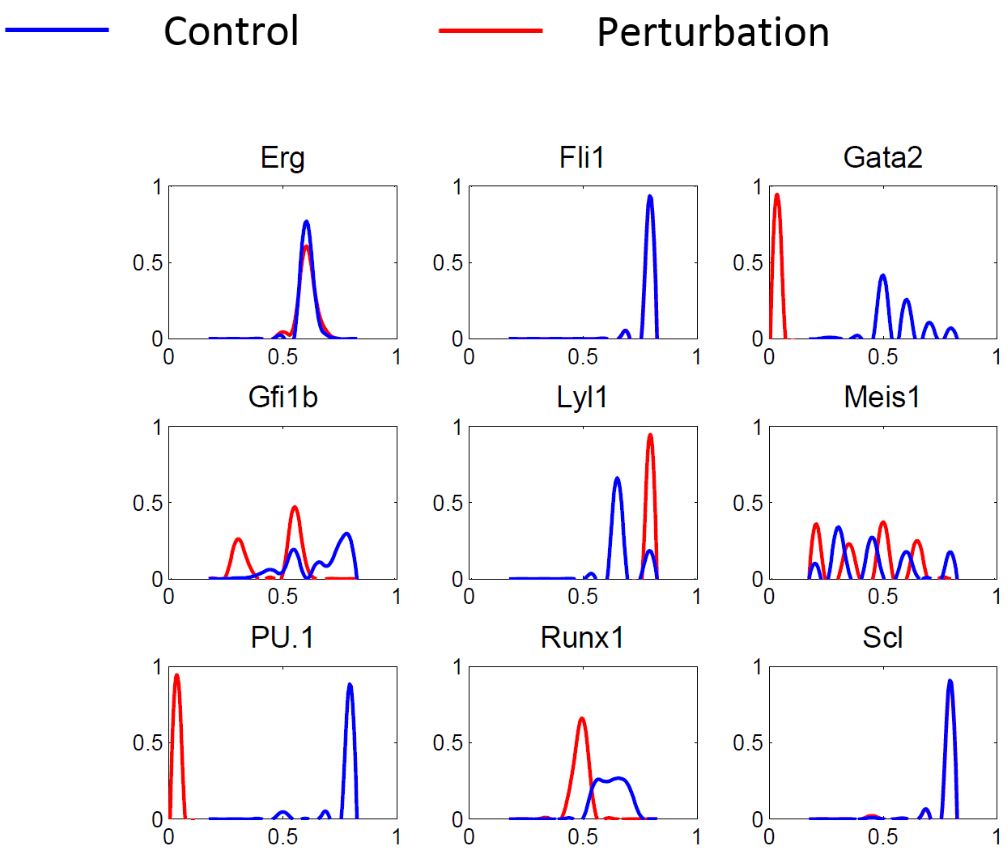
Control
Perturbation
Erg
Fli1
Gata2
Gfi1b
Lyl1
Meis1
PU.1
Runx1
Scl

Top

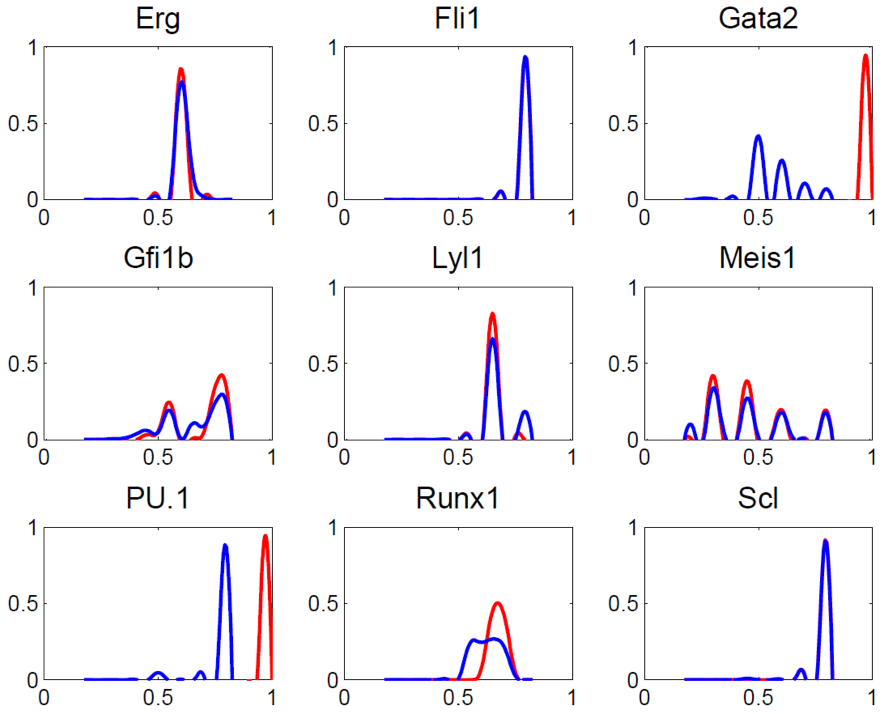
Erg
Fli1
Gata2
Gfi1b
Lyl1
Meis1
PU.1
Runx1
Scl

Top

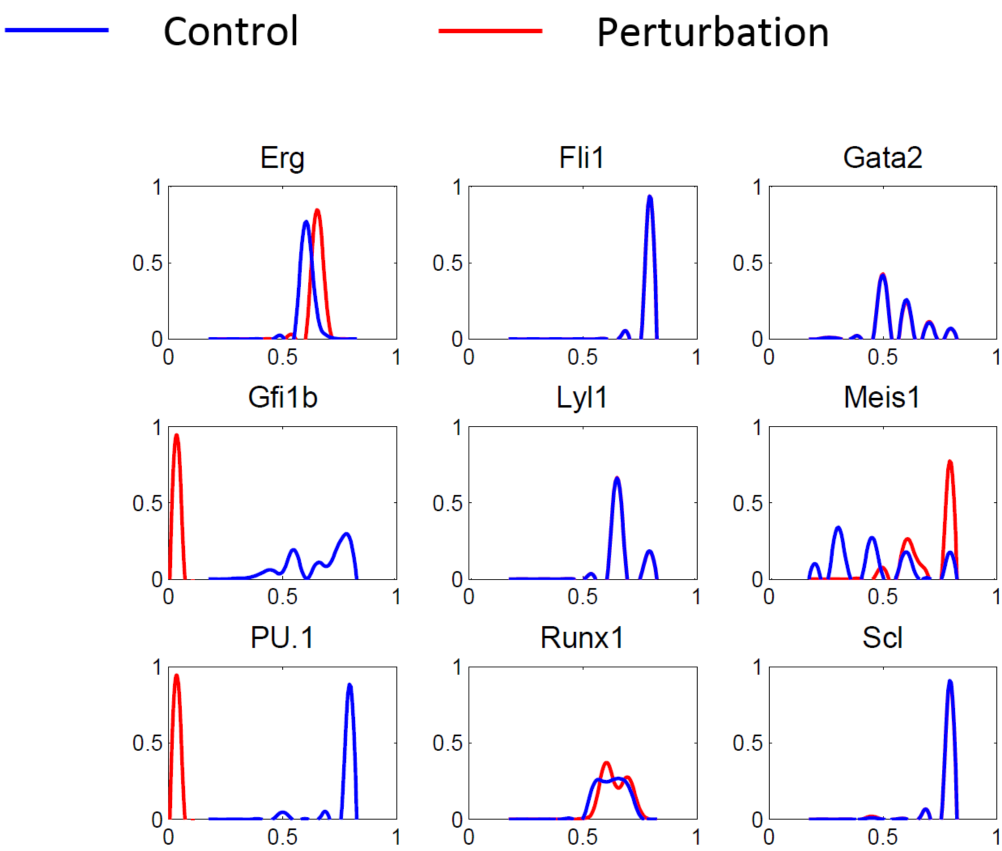
Control
Perturbation
Erg
Fli1
Gata2
Gfi1b
Lyl1
Meis1
PU.1
Runx1
Scl

Top

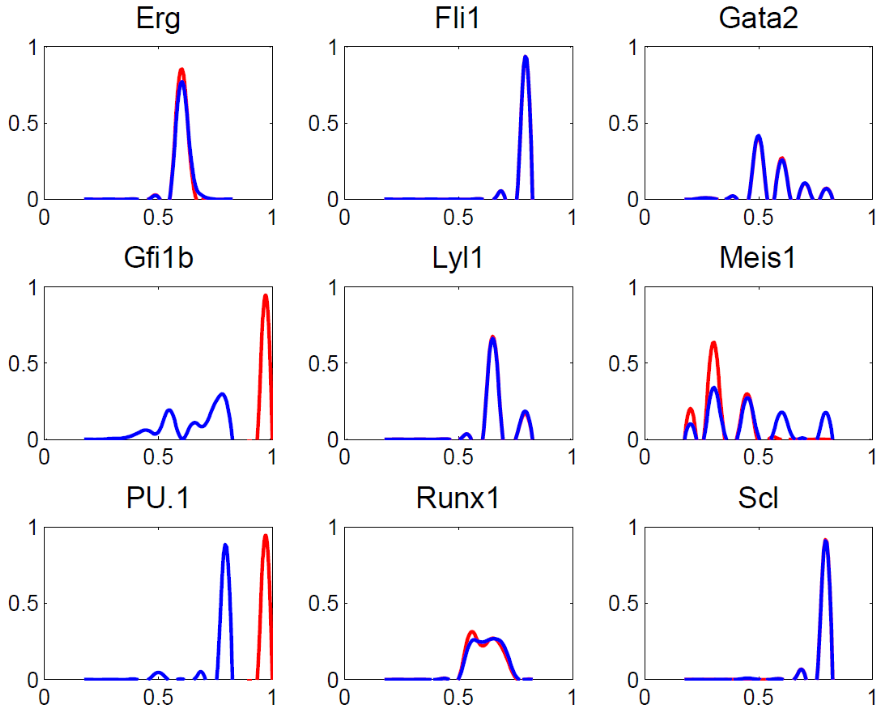
Erg
Fli1
Gata2
Gfi1b
Lyl1
Meis1
PU.1
Runx1
Scl

Top

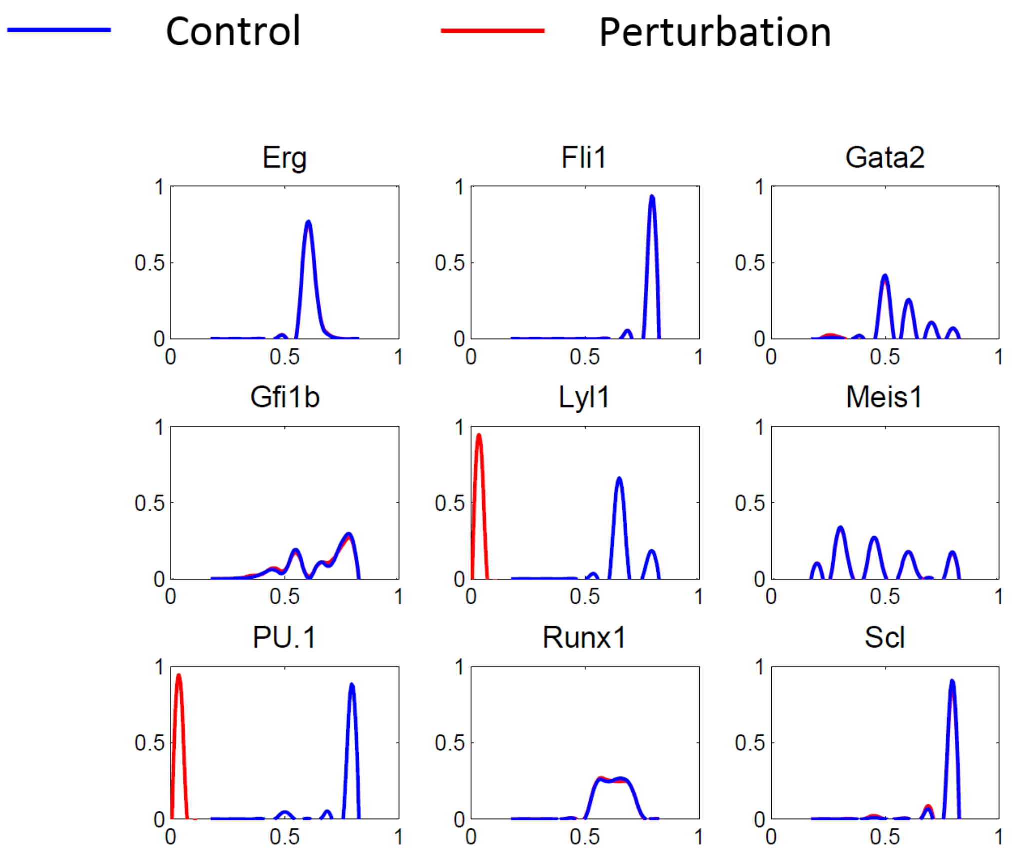
Control
Perturbation
Erg
Fli1
Gata2
Gfi1b
Lyl1
Meis1
PU.1
Runx1
Scl

[Top](#)

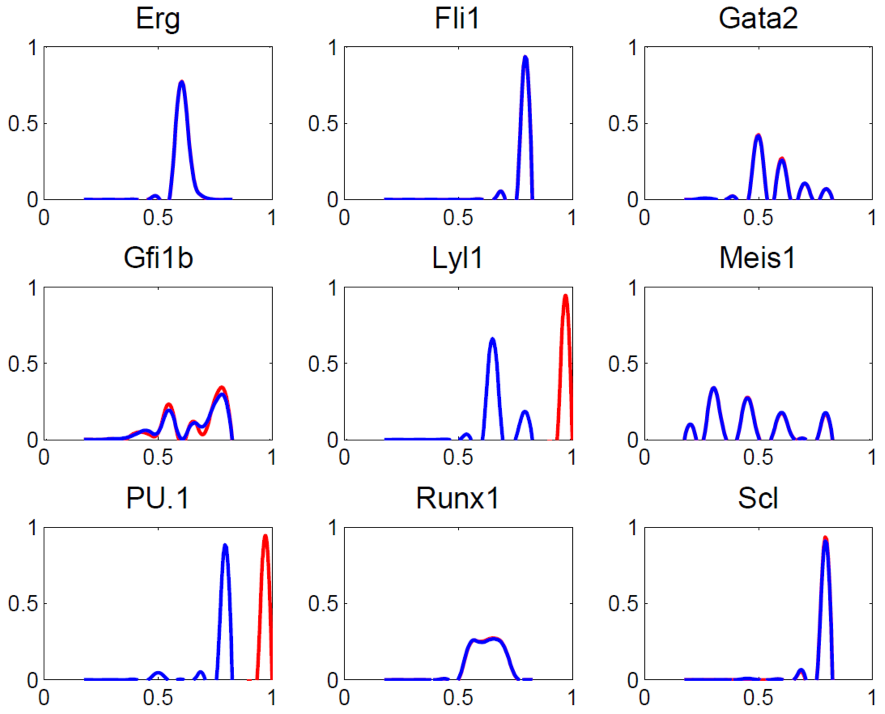
Erg
Fli1
Gata2
Gfi1b
Lyl1
Meis1
PU.1
Runx1
Scl

[Top](#)

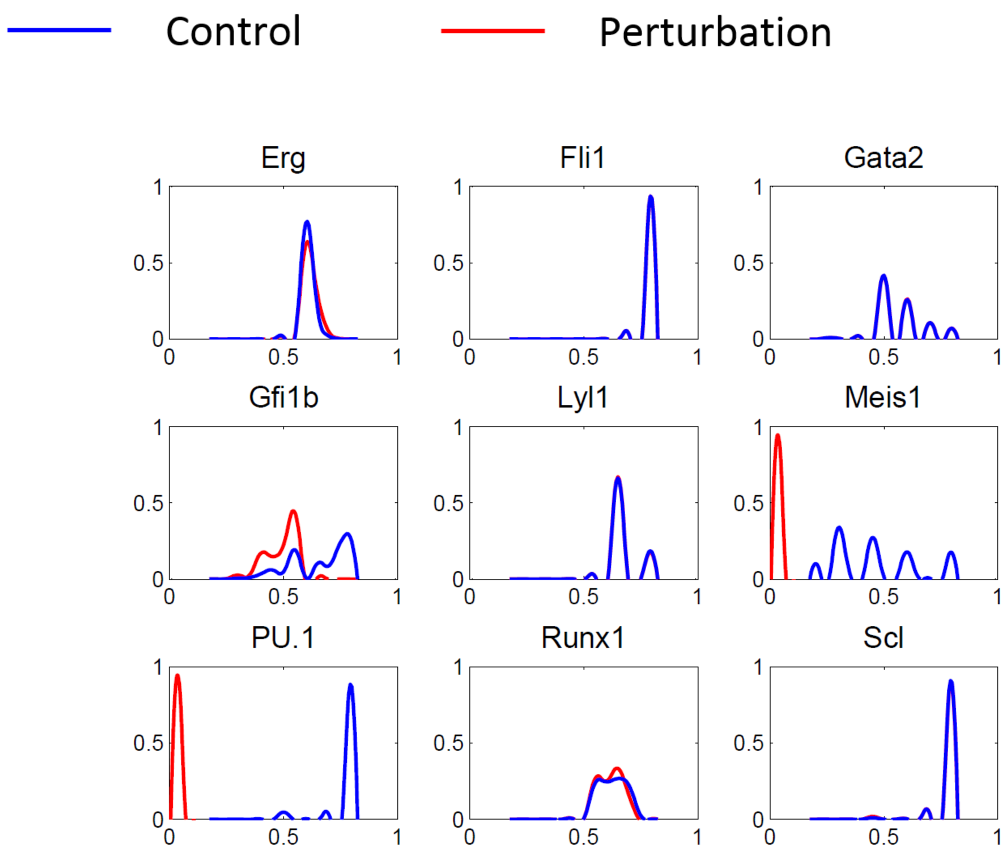
Control
Perturbation
Erg
Fli1
Gata2
Gfi1b
Lyl1
Meis1
PU.1
Runx1
Scl

Top

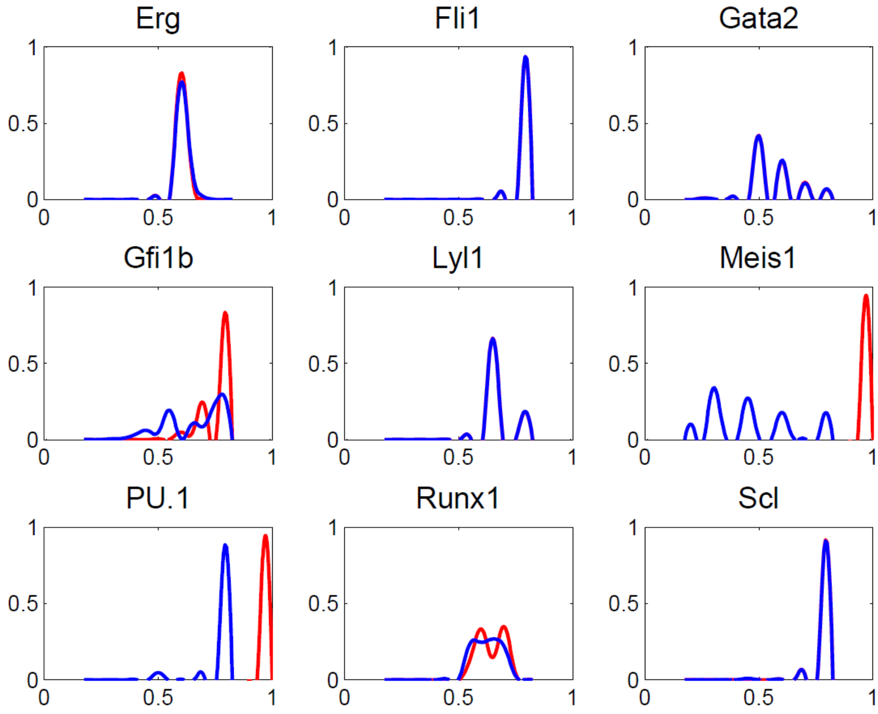
Erg
Fli1
Gata2
Gfi1b
Lyl1
Meis1
PU.1
Runx1
Scl

Top

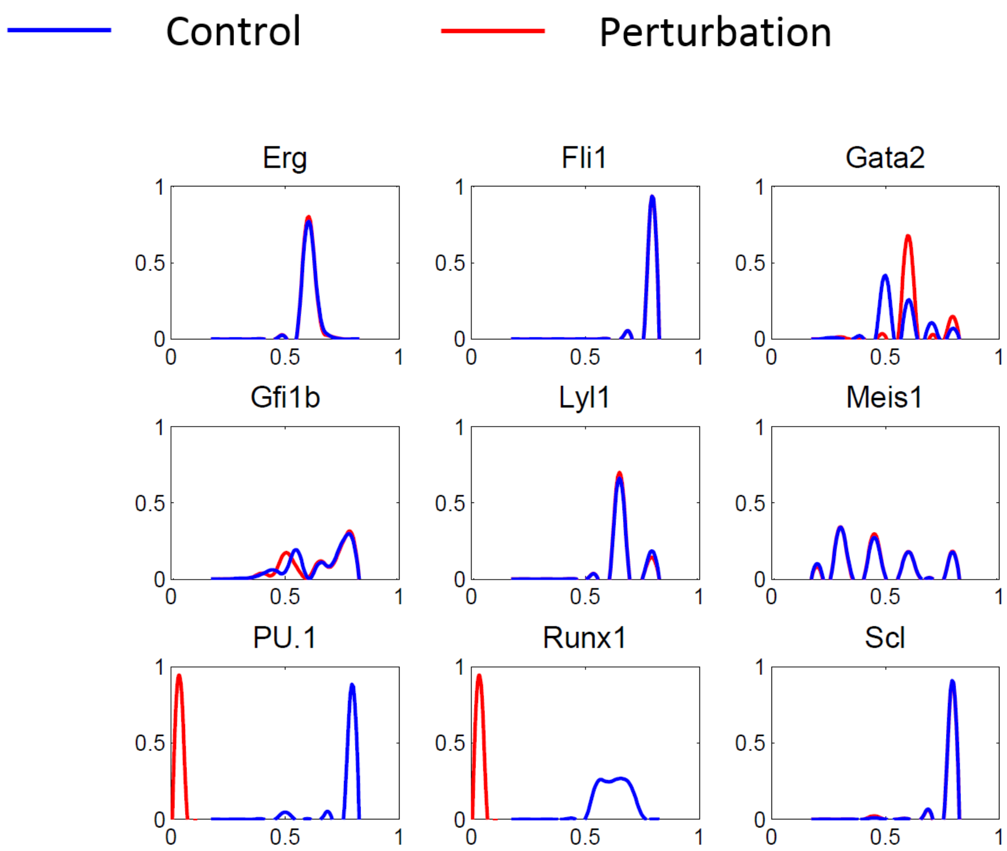
Control
Perturbation
Erg
Fli1
Gata2
Gfi1b
Lyl1
Meis1
PU.1
Runx1
Scl

Top

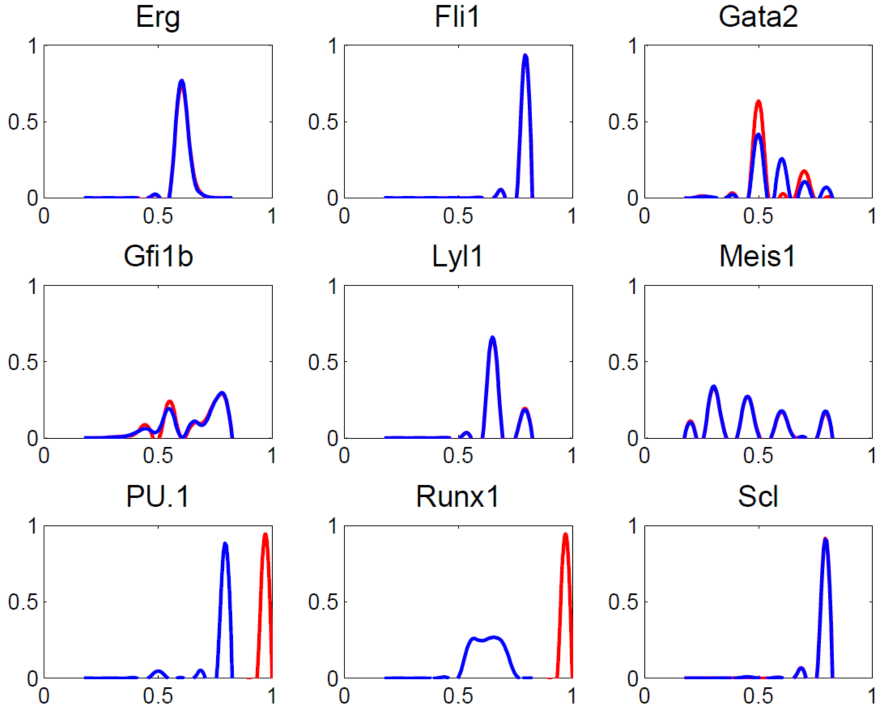
Erg
Fli1
Gata2
Gfi1b
Lyl1
Meis1
PU.1
Runx1
Scl

Top

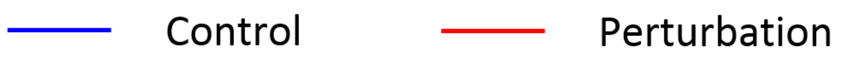
Control
Perturbation

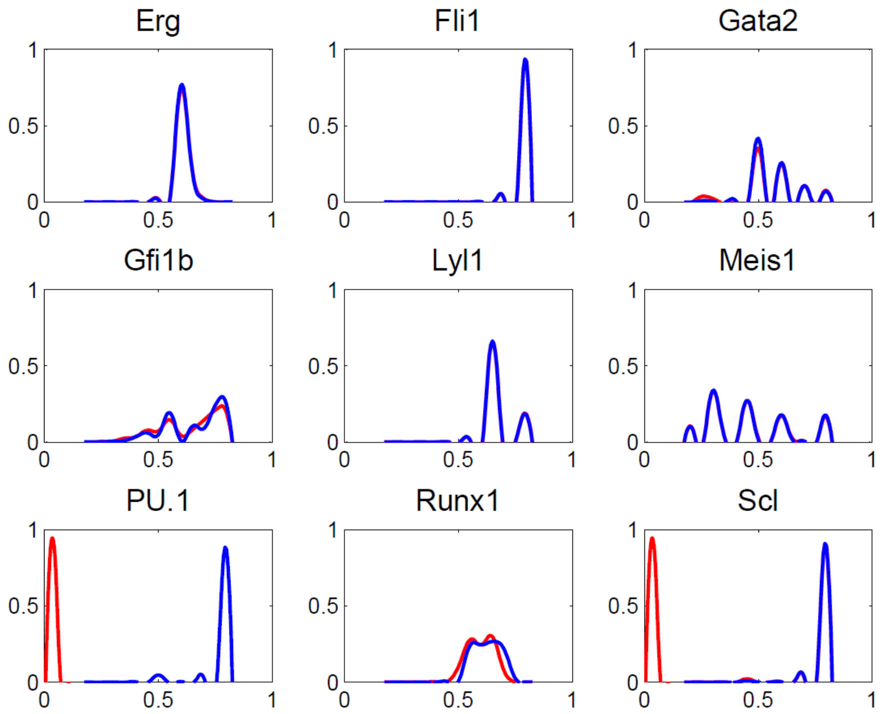
Erg
Fli1
Gata2
Gfi1b
Lyl1
Meis1
PU.1
Runx1
Scl

Top

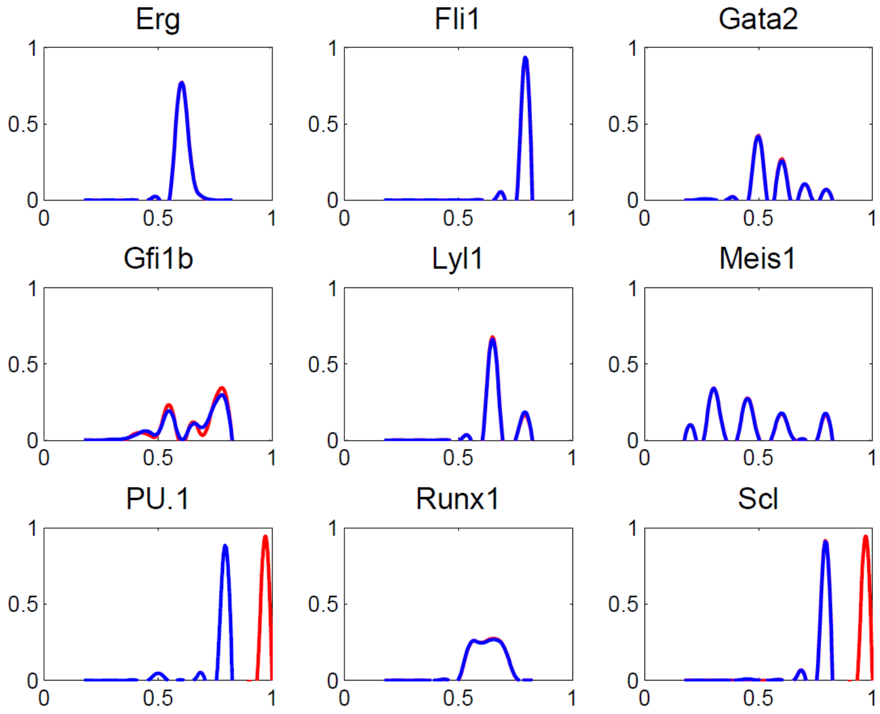
Erg
Fli1
Gata2
Gfi1b
Lyl1
Meis1
PU.1
Runx1
Scl

Top

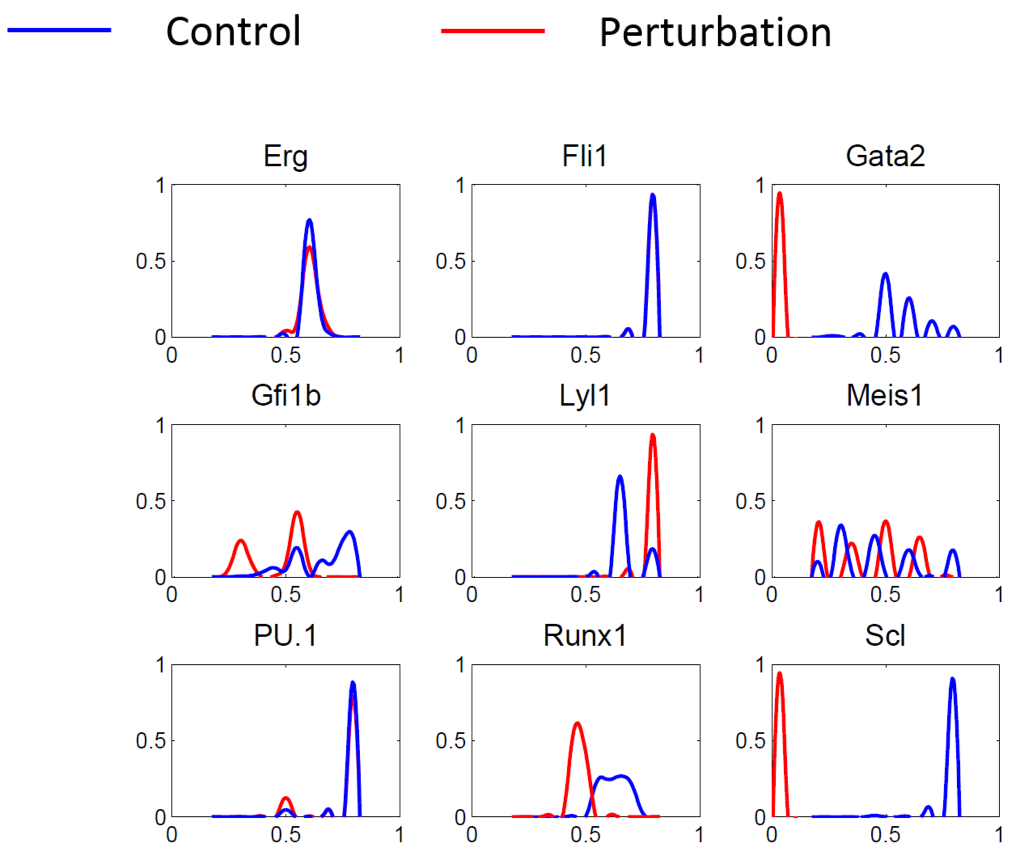
Control
Perturbation
Erg
Fli1
Gata2
Gfi1b
Lyl1
Meis1
PU.1
Runx1
Scl
0
0.5
1

Top

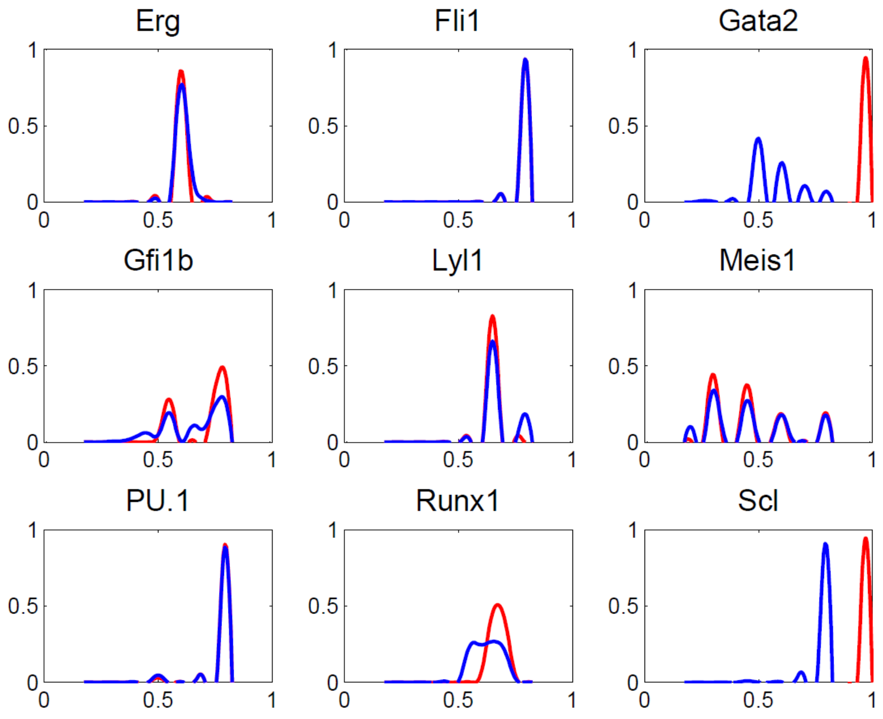
Erg
Fli1
Gata2
Gfi1b
Lyl1
Meis1
PU.1
Runx1
Scl
0
0.5
1

Top

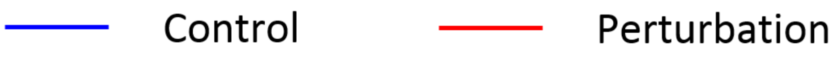
Control
Perturbation

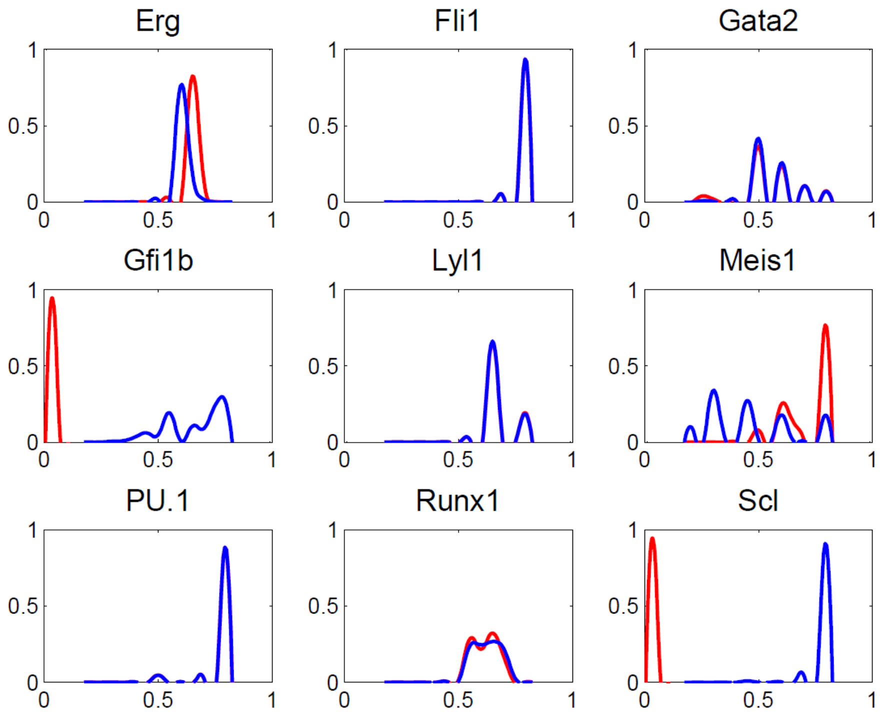
Erg
Fli1
Gata2
Gfi1b
Lyl1
Meis1
PU.1
Runx1
Scl

Top

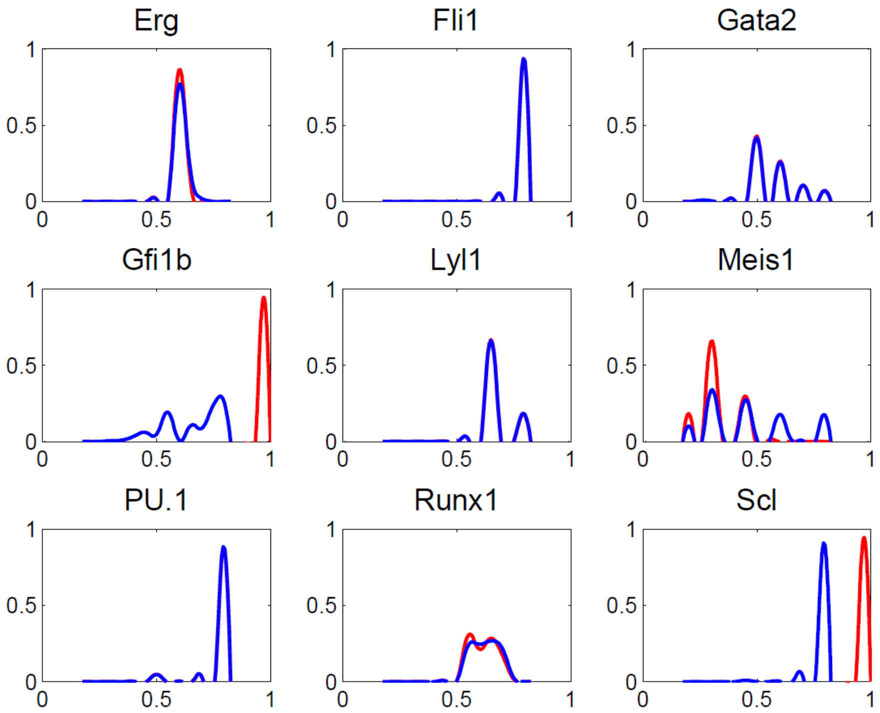
Erg
Fli1
Gata2
Gfi1b
Lyl1
Meis1
PU.1
Runx1
Scl

Top

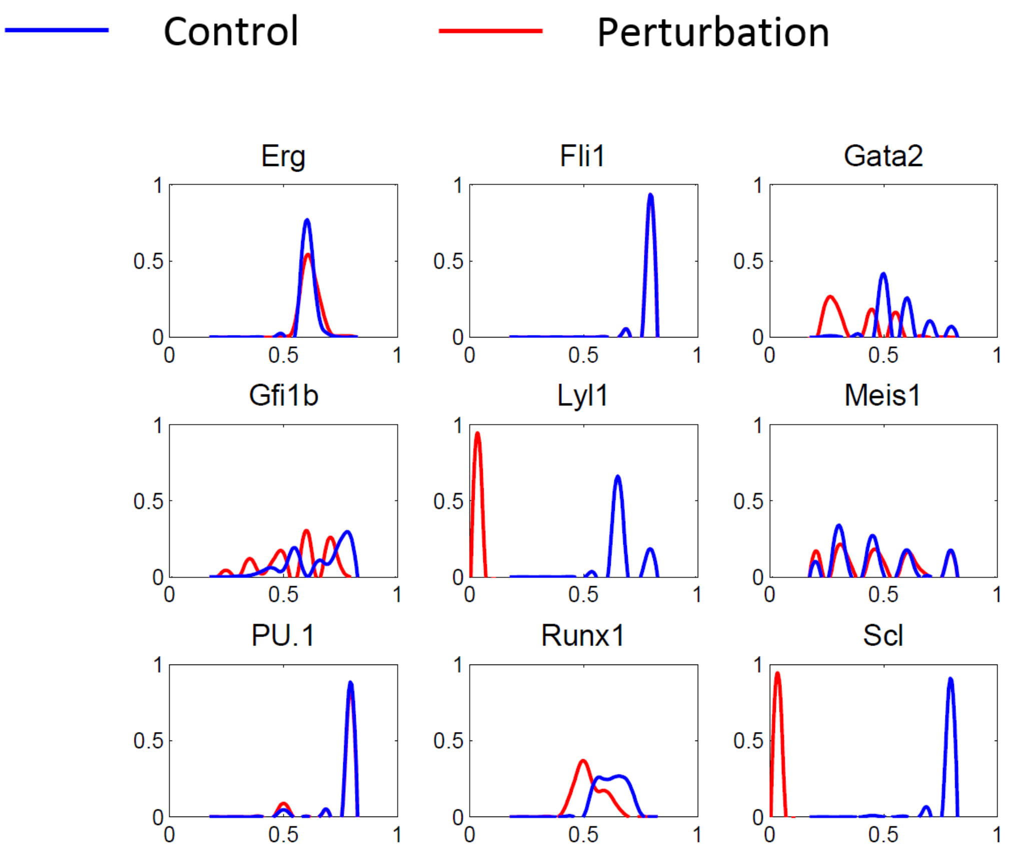
Control
Perturbation
Erg
Fli1
Gata2
Gfi1b
Lyl1
Meis1
PU.1
Runx1
Scl

Top

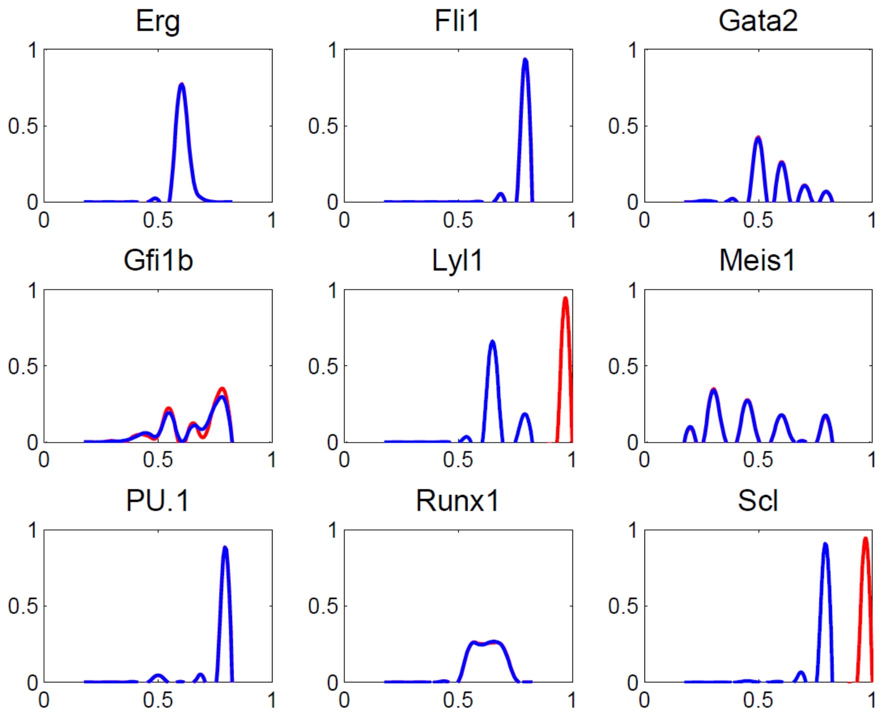
Erg
Fli1
Gata2
Gfi1b
Lyl1
Meis1
PU.1
Runx1
Scl

Top

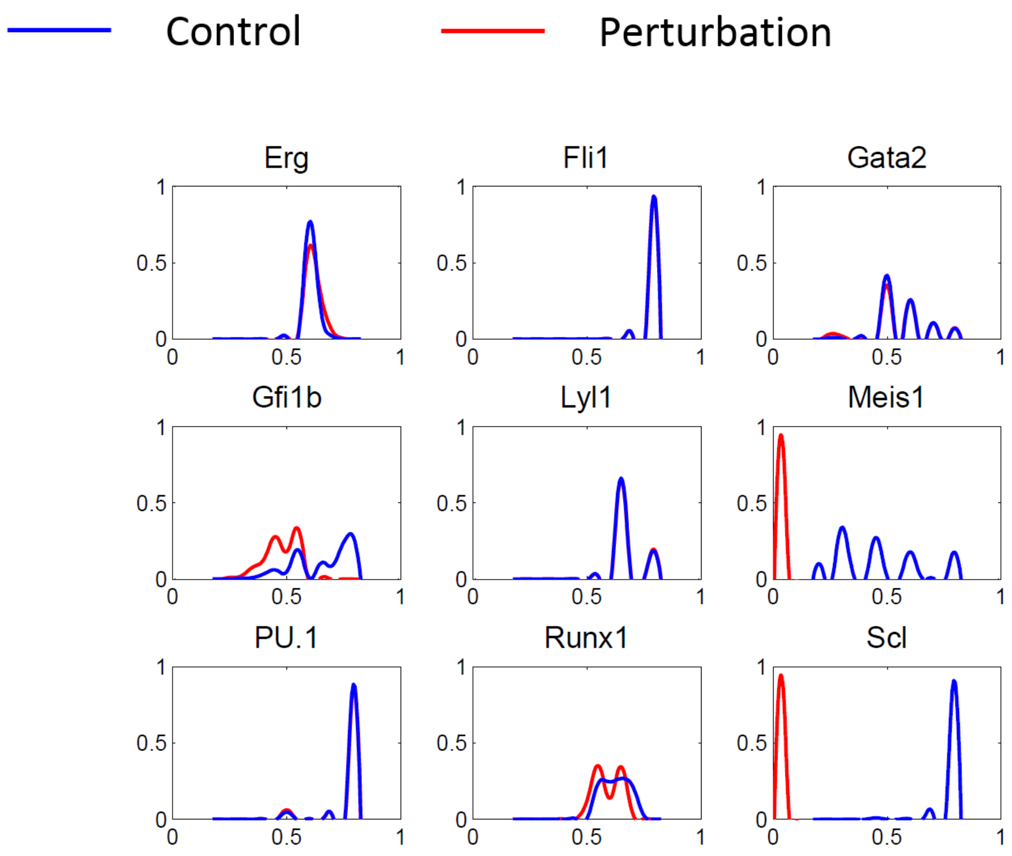
Control
Perturbation
Erg
Fli1
Gata2
Gfi1b
Lyl1
Meis1
PU.1
Runx1
Scl

Top

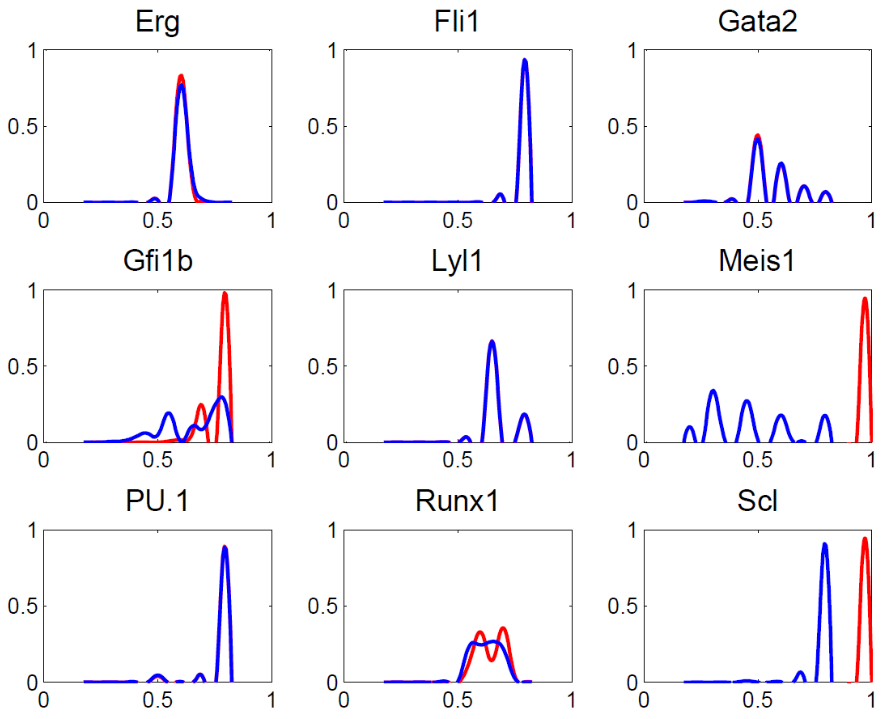
Erg
Fli1
Gata2
Gfi1b
Lyl1
Meis1
PU.1
Runx1
Scl

Top

Control Perturbation

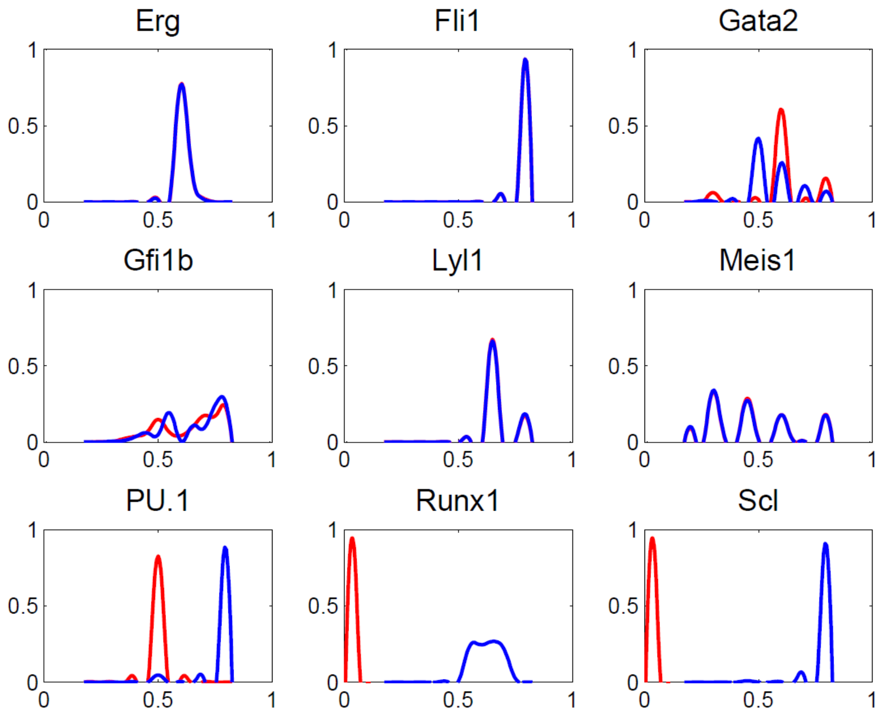

Top

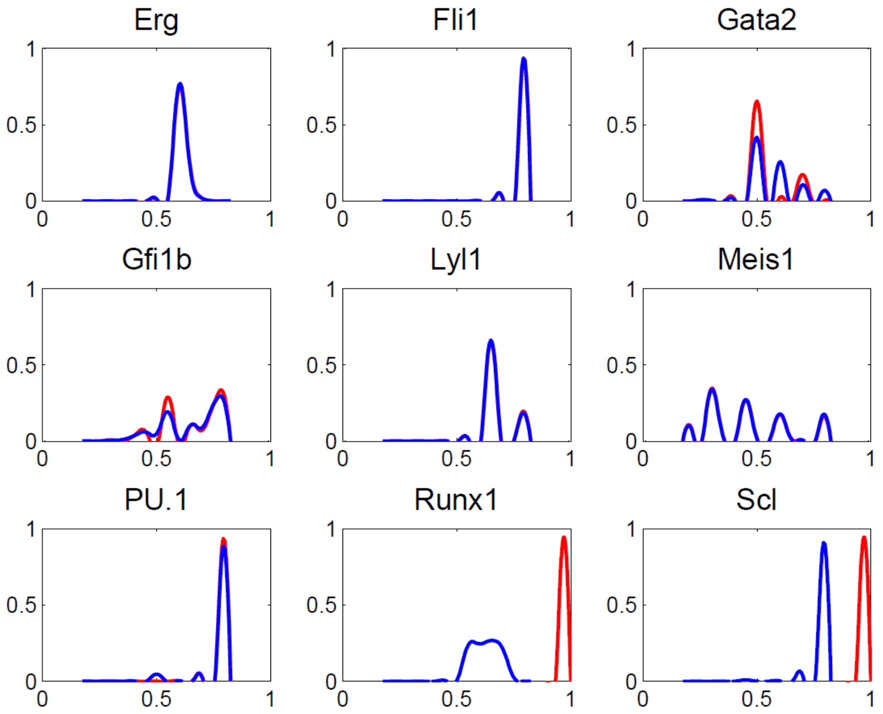

Top

Control   Perturbation

In each case:
one TF up and one TF down

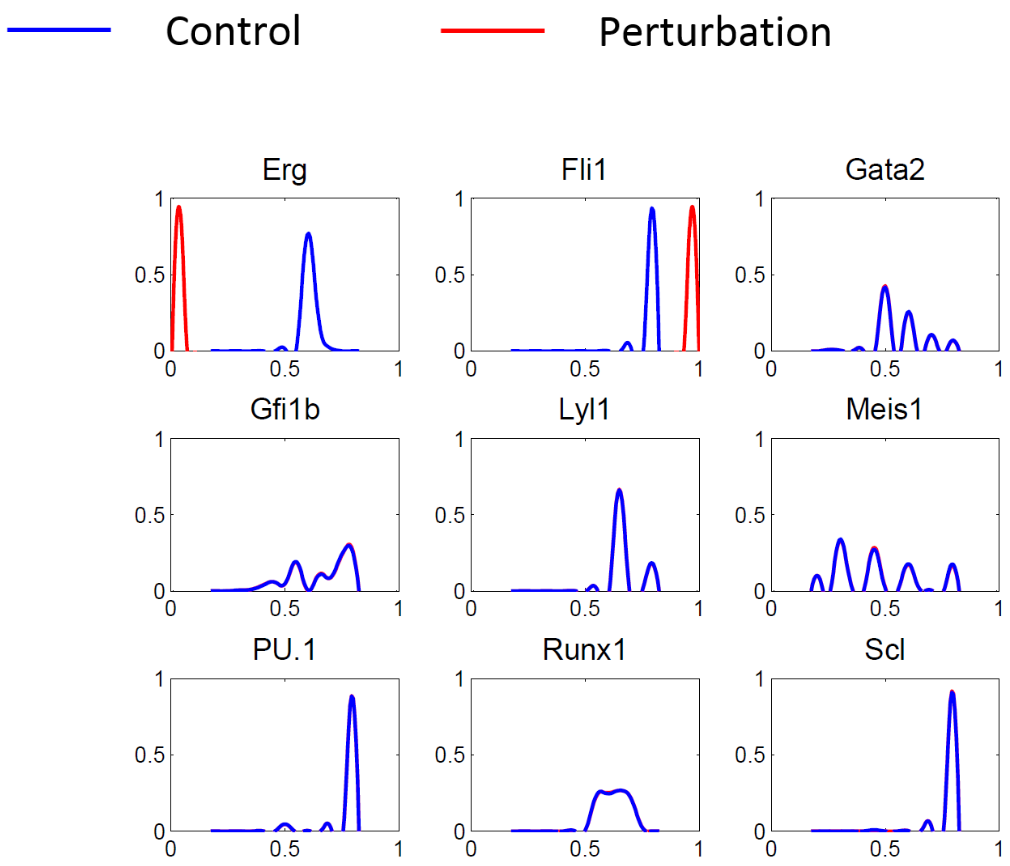
Control
Perturbation
Erg
Fli1
Gata2
Gfi1b
Lyl1
Meis1
PU.1
Runx1
Scl

Top

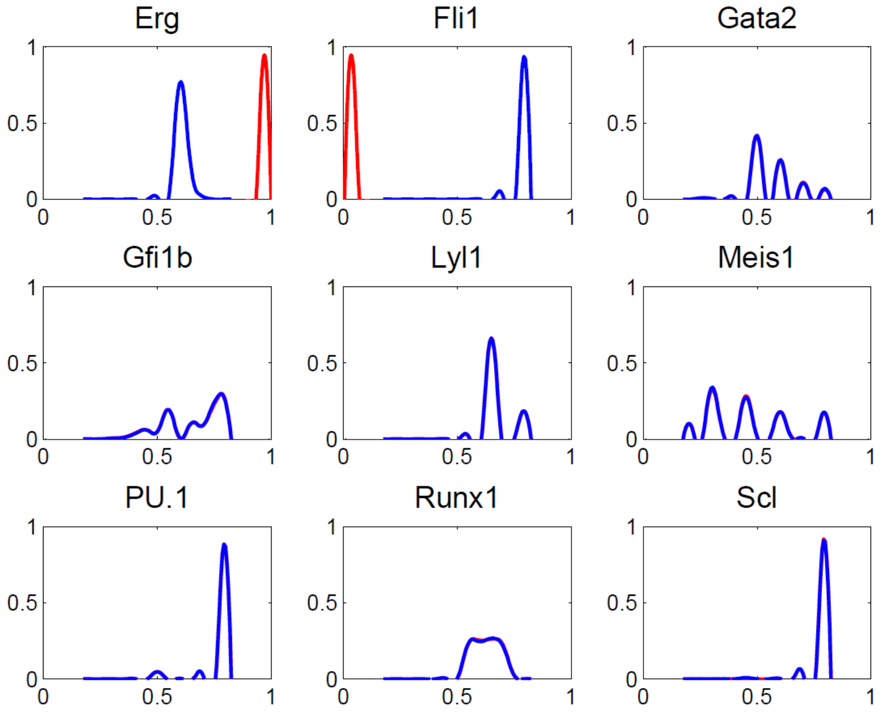
Erg
Fli1
Gata2
Gfi1b
Lyl1
Meis1
PU.1
Runx1
Scl

Top

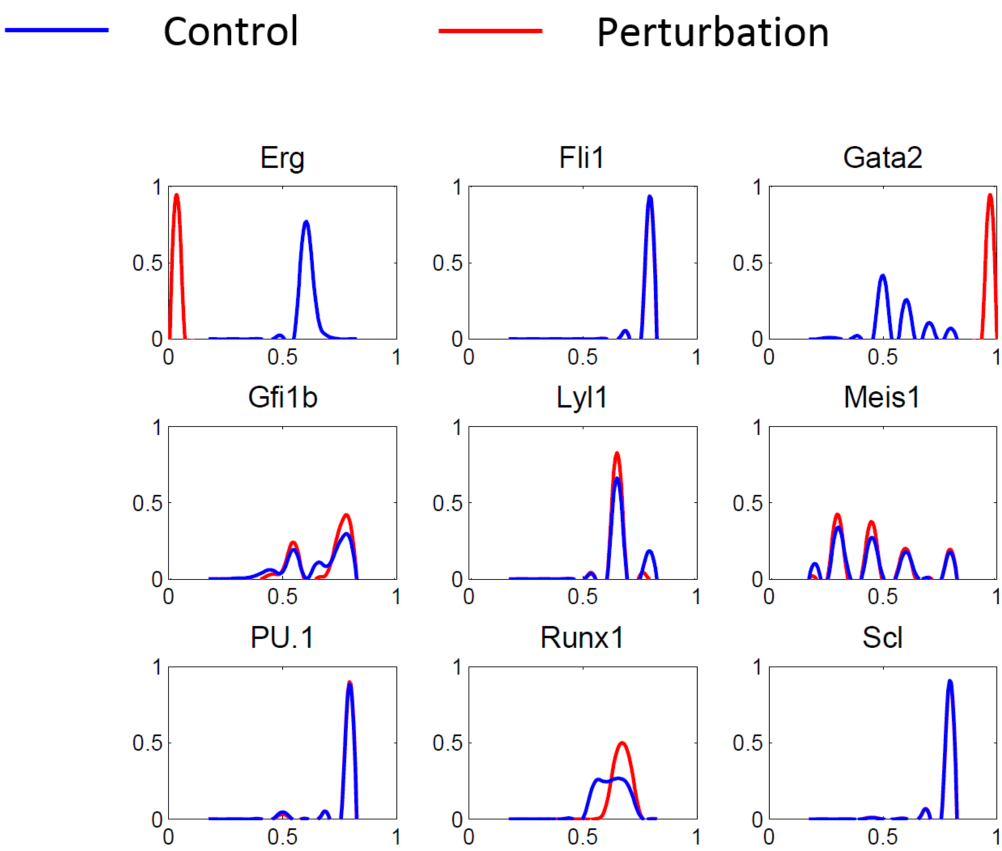
Control
Perturbation
Erg
Fli1
Gata2
Gfi1b
Lyl1
Meis1
PU.1
Runx1
Scl

Top

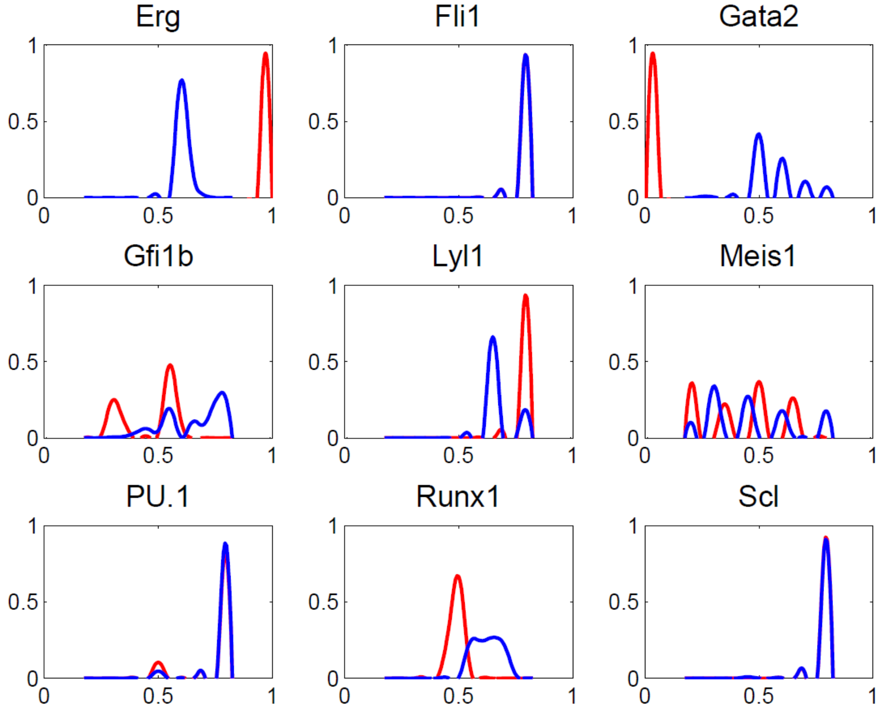
Erg
Fli1
Gata2
Gfi1b
Lyl1
Meis1
PU.1
Runx1
Scl

Top

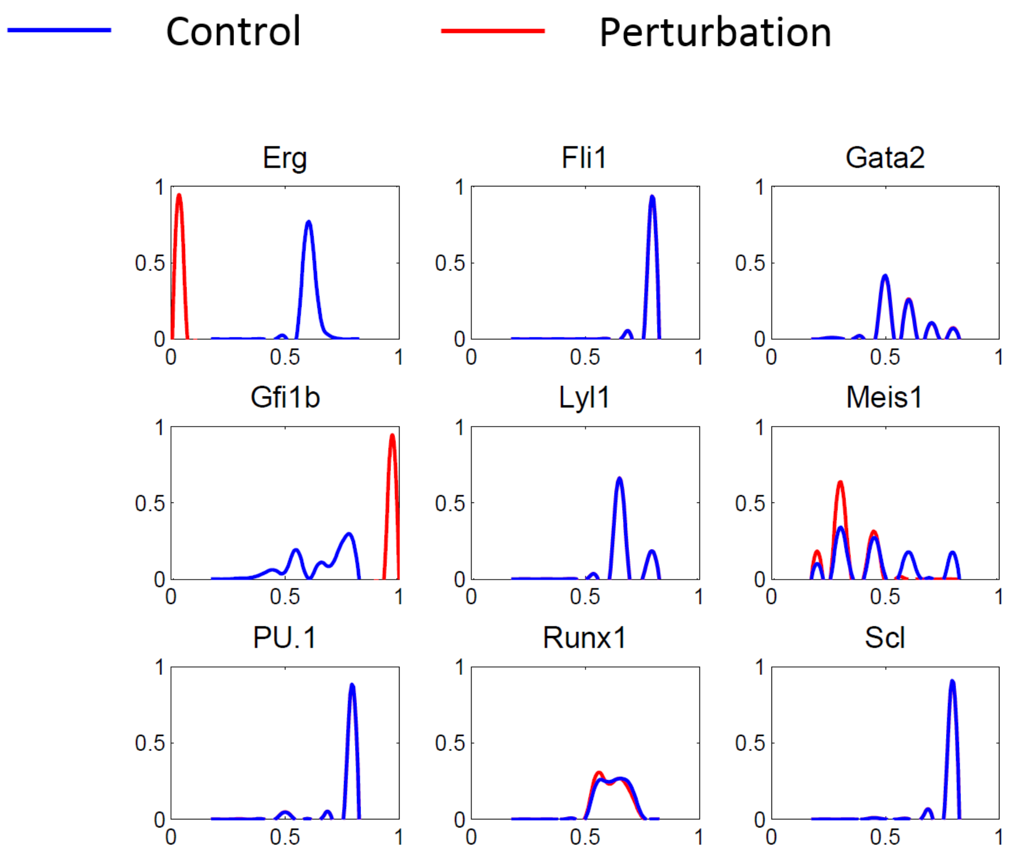
Control
Perturbation
Erg
Fli1
Gata2
Gfi1b
Lyl1
Meis1
PU.1
Runx1
Scl
0
0.5
1

Top

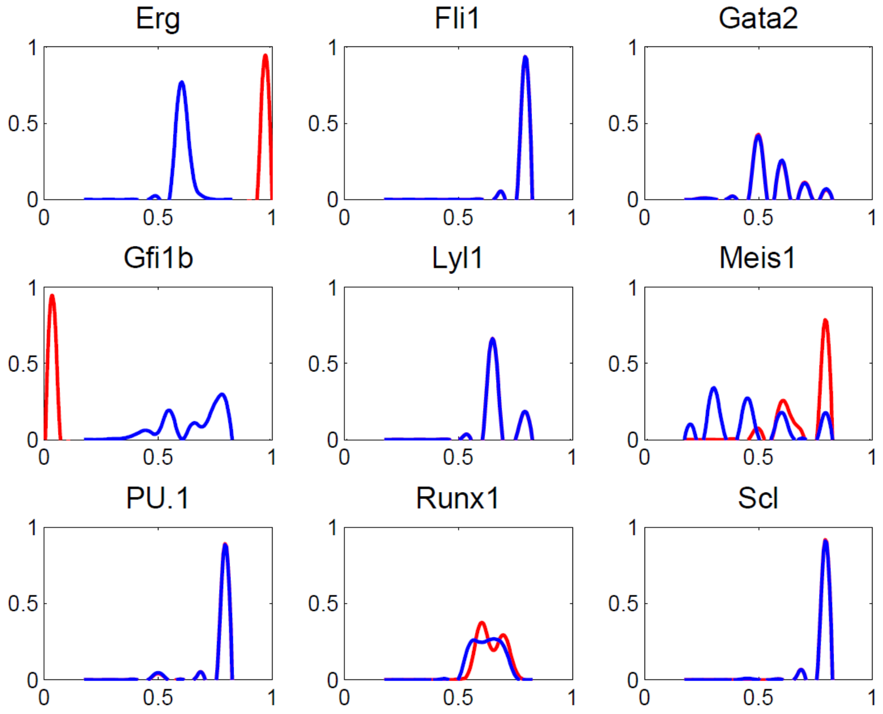
Erg
Fli1
Gata2
Gfi1b
Lyl1
Meis1
PU.1
Runx1
Scl
0
0.5
1

Top

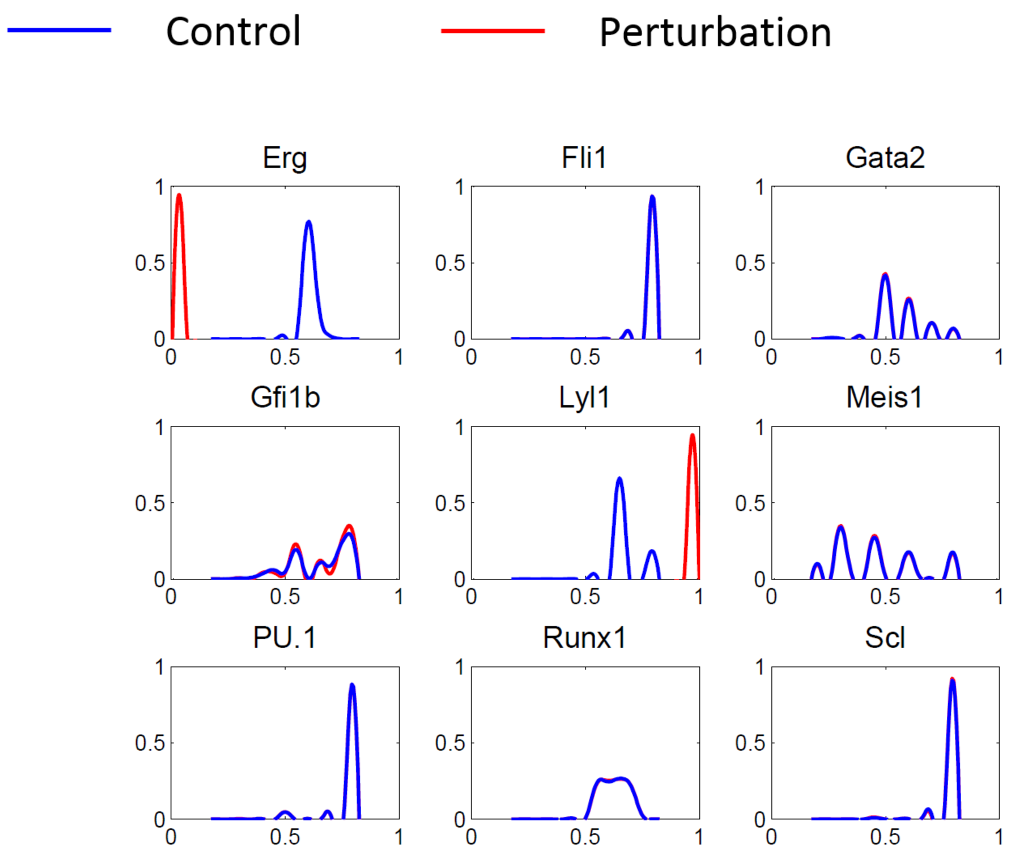
Control
Perturbation
Erg
Fli1
Gata2
Gfi1b
Lyl1
Meis1
PU.1
Runx1
Scl

Top

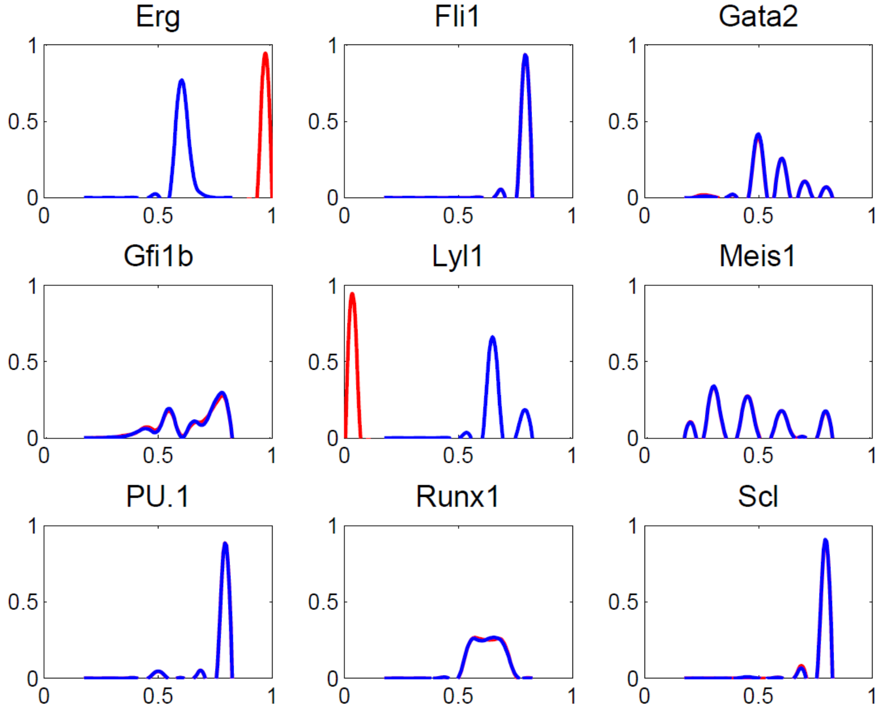
Erg
Fli1
Gata2
Gfi1b
Lyl1
Meis1
PU.1
Runx1
Scl

Top

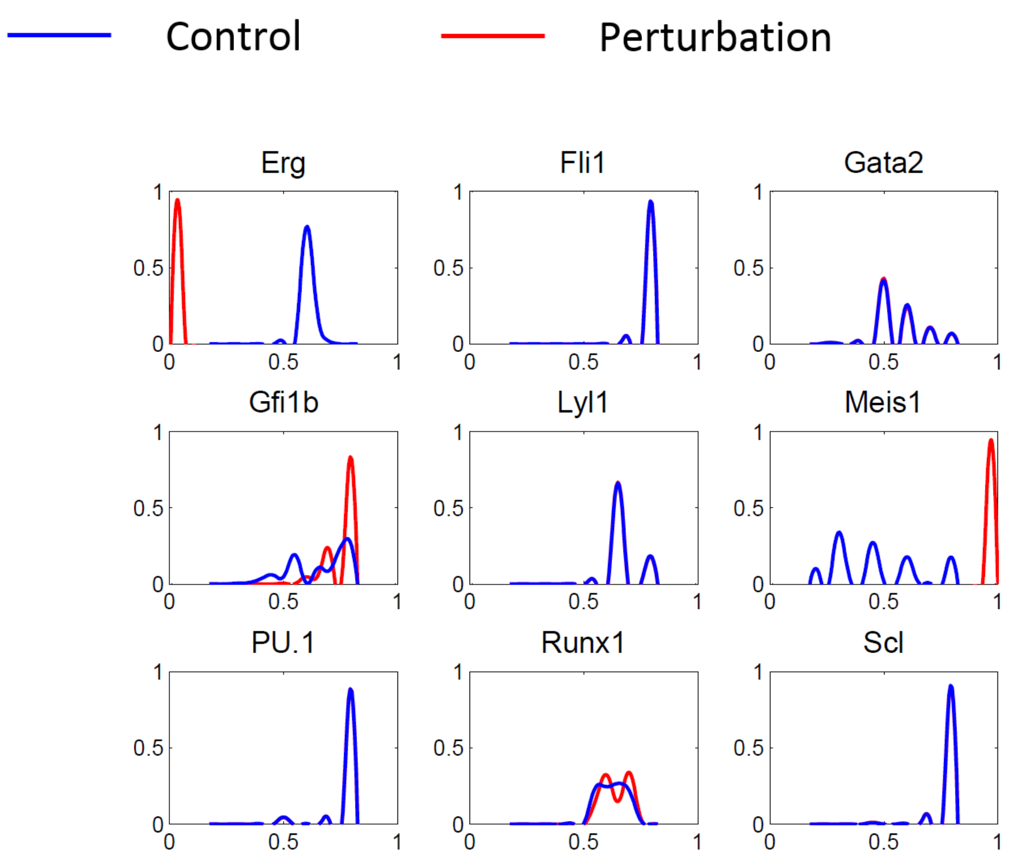
Control
Perturbation
Erg
Fli1
Gata2
Gfi1b
Lyl1
Meis1
PU.1
Runx1
Scl

Top

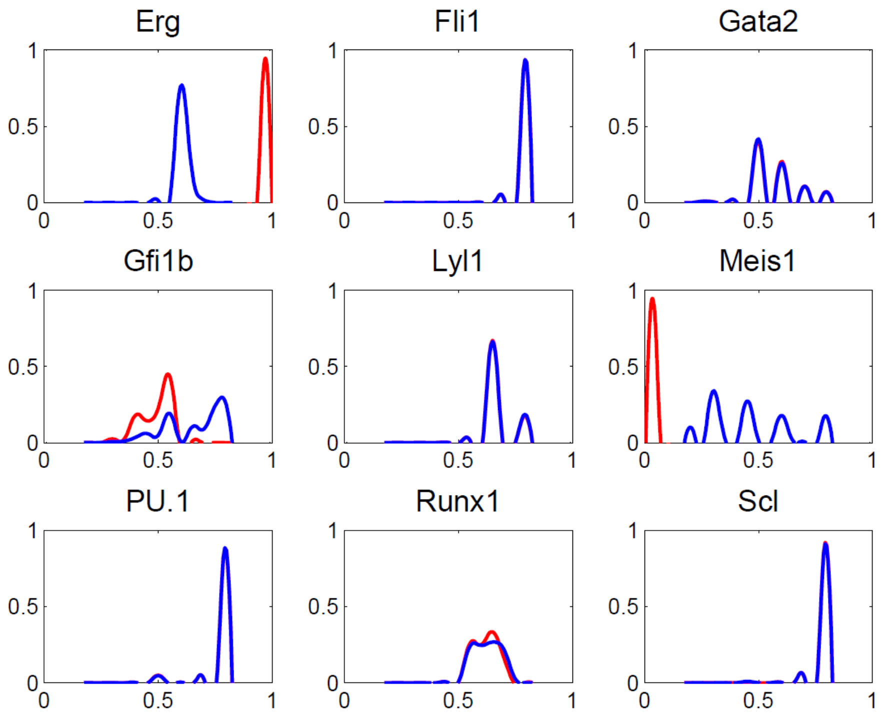
Erg
Fli1
Gata2
Gfi1b
Lyl1
Meis1
PU.1
Runx1
Scl

Top

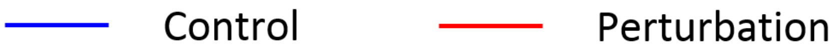
Control
Perturbation

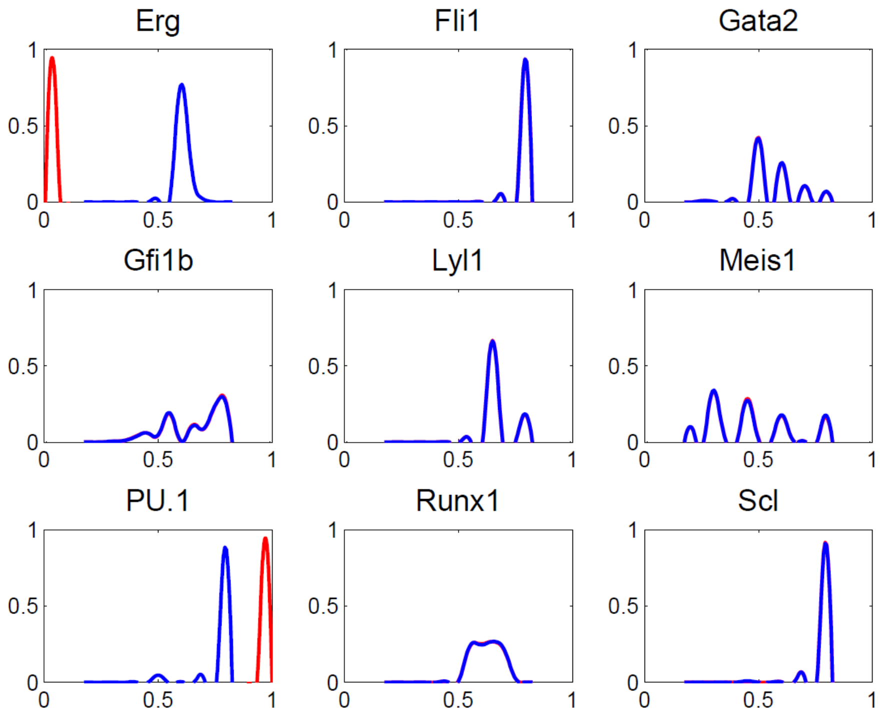
Erg
Fli1
Gata2
Gfi1b
Lyl1
Meis1
PU.1
Runx1
Scl

Top

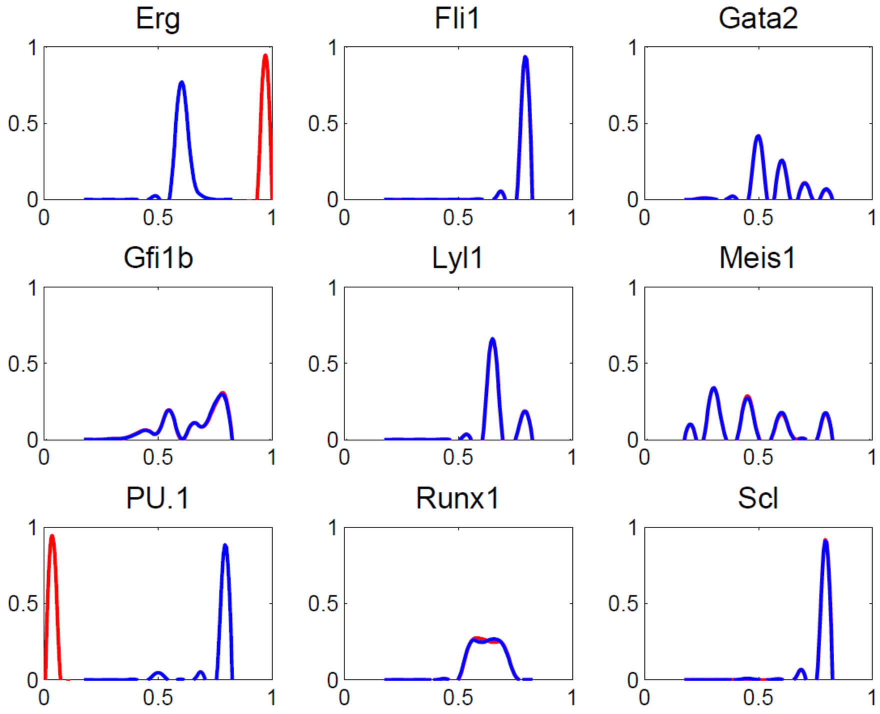
Erg
Fli1
Gata2
Gfi1b
Lyl1
Meis1
PU.1
Runx1
Scl

Top

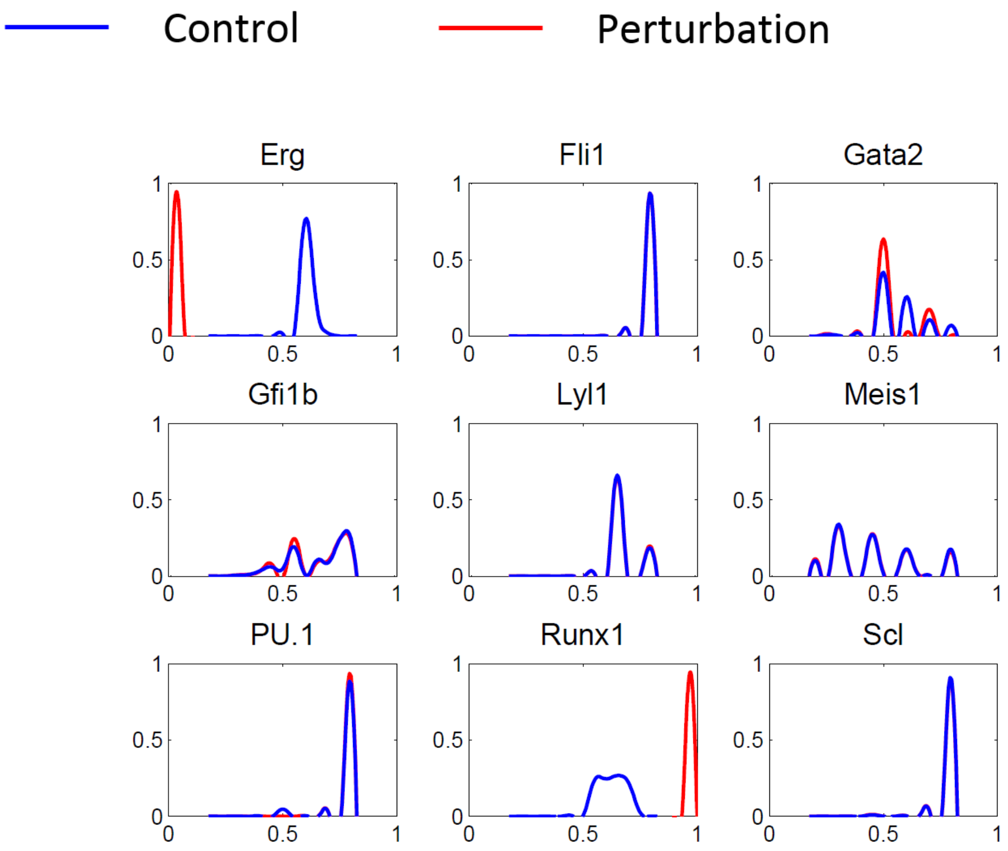
Control
Perturbation
Erg
Fli1
Gata2
Gfi1b
Lyl1
Meis1
PU.1
Runx1
Scl

Top

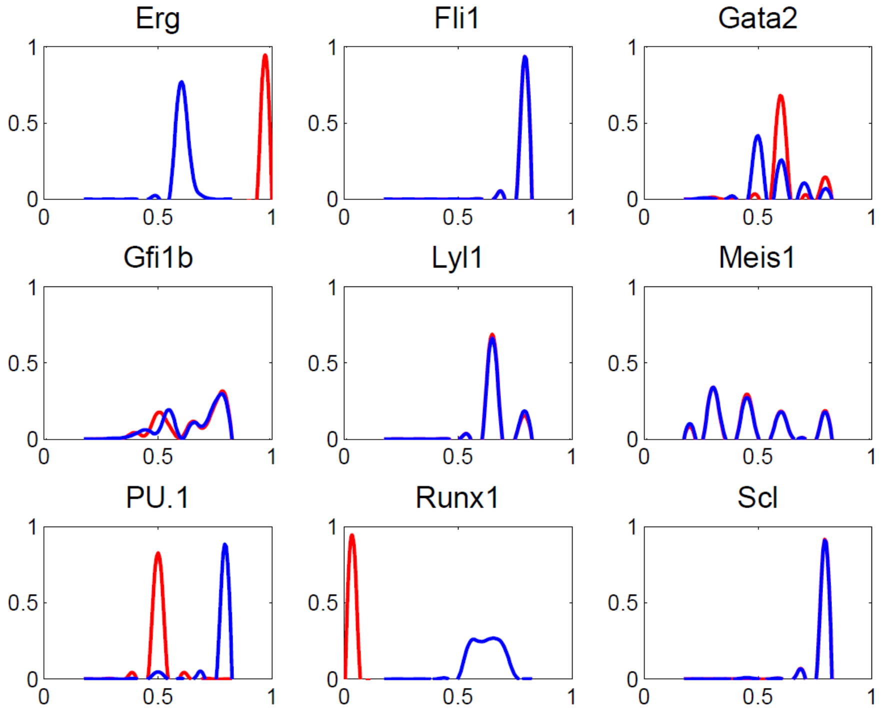
Erg
Fli1
Gata2
Gfi1b
Lyl1
Meis1
PU.1
Runx1
Scl

Top

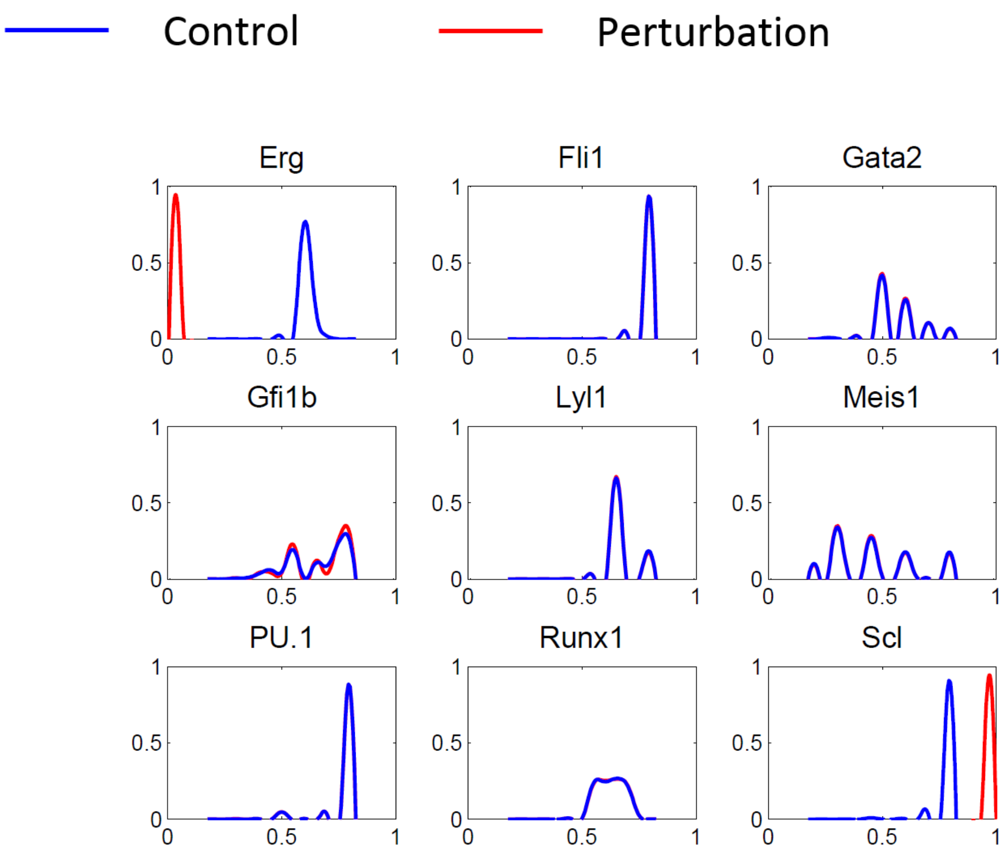
Control
Perturbation
Erg
Fli1
Gata2
Gfi1b
Lyl1
Meis1
PU.1
Runx1
Scl

Top

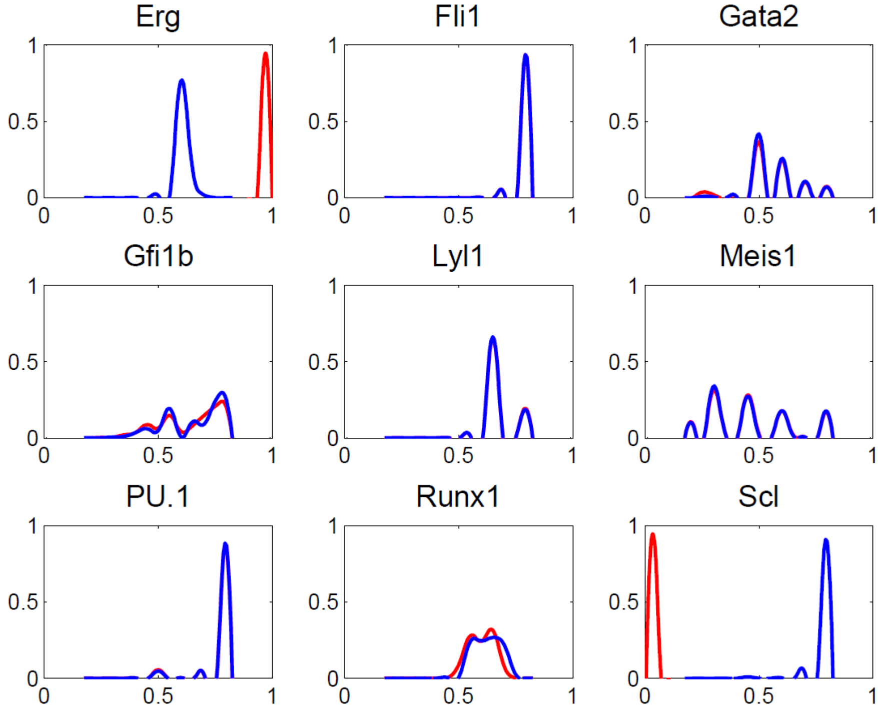
Erg
Fli1
Gata2
Gfi1b
Lyl1
Meis1
PU.1
Runx1
Scl

Top

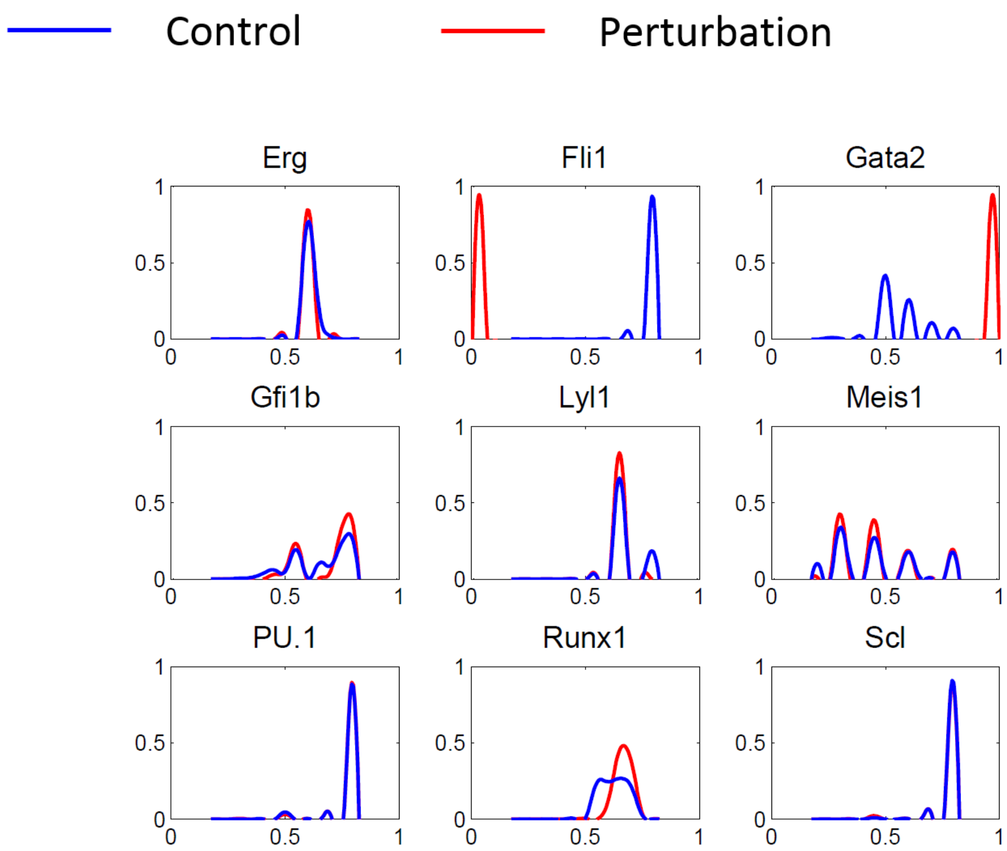
Control
Perturbation
Erg
Fli1
Gata2
Gfi1b
Lyl1
Meis1
PU.1
Runx1
Scl

Top

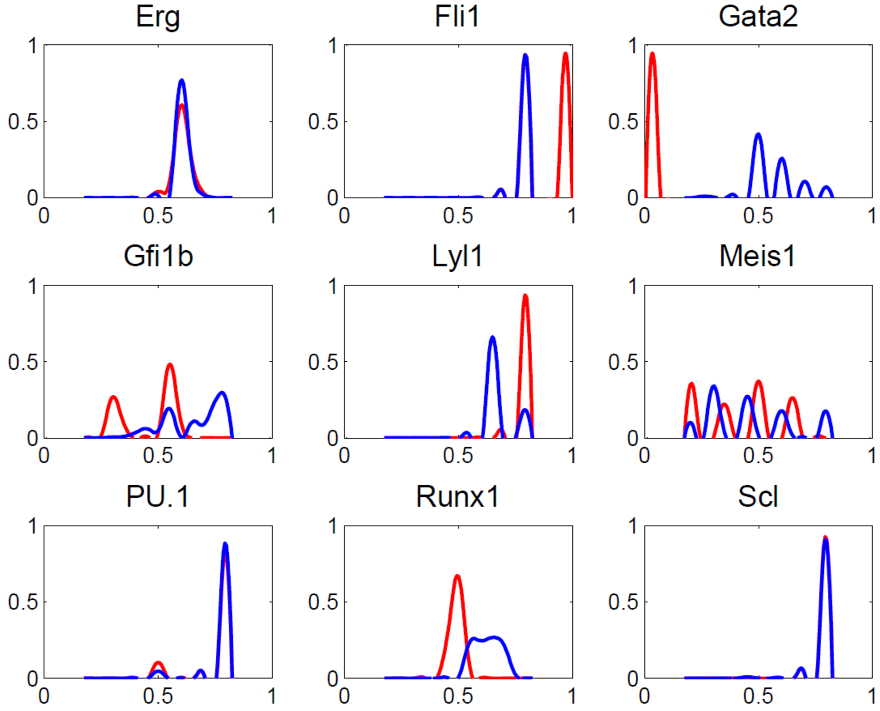
Erg
Fli1
Gata2
Gfi1b
Lyl1
Meis1
PU.1
Runx1
Scl

Top

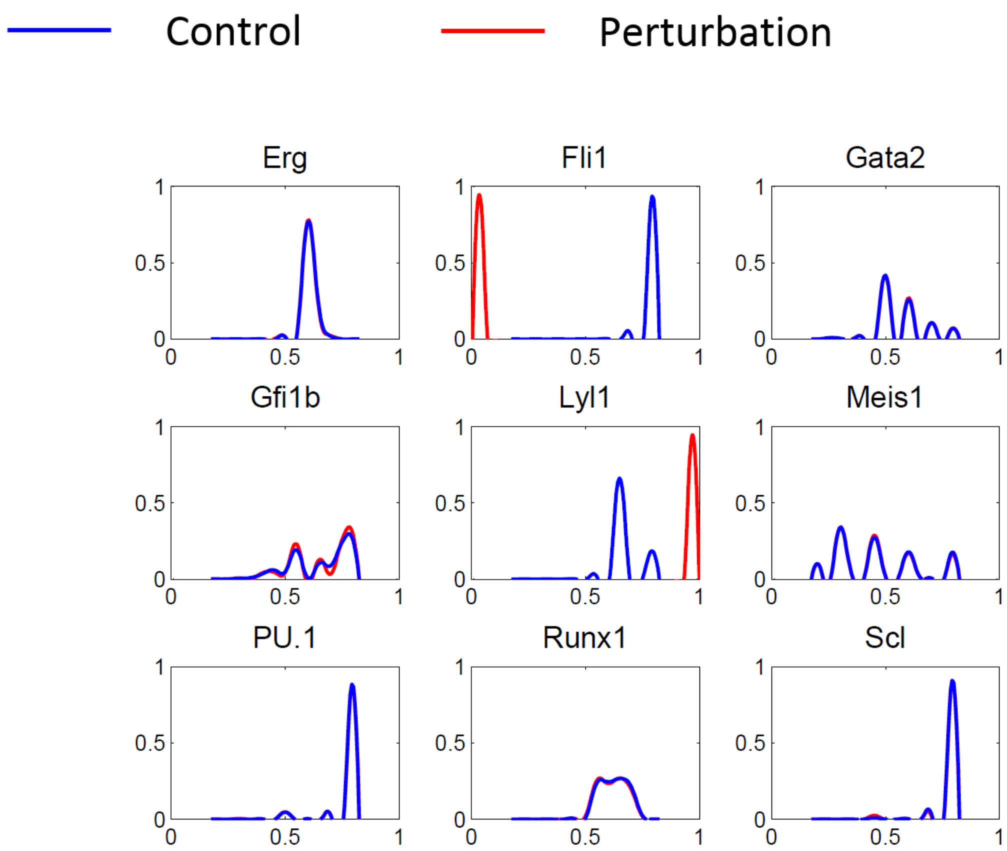
Control
Perturbation
Erg
Fli1
Gata2
Gfi1b
Lyl1
Meis1
PU.1
Runx1
Scl
0
0.5
1

Top

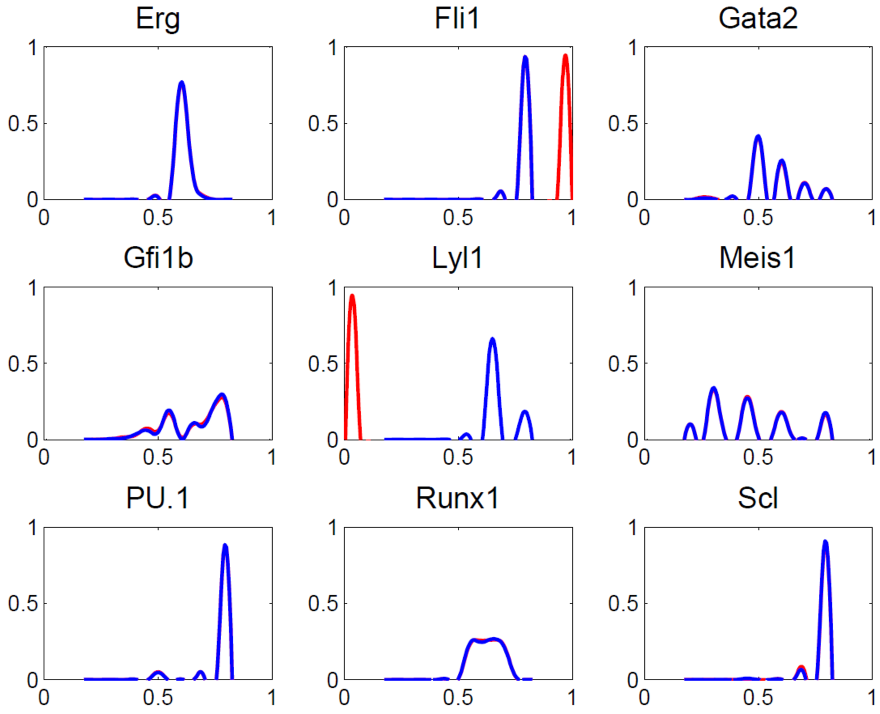
Erg
Fli1
Gata2
Gfi1b
Lyl1
Meis1
PU.1
Runx1
Scl
0
0.5
1

Top

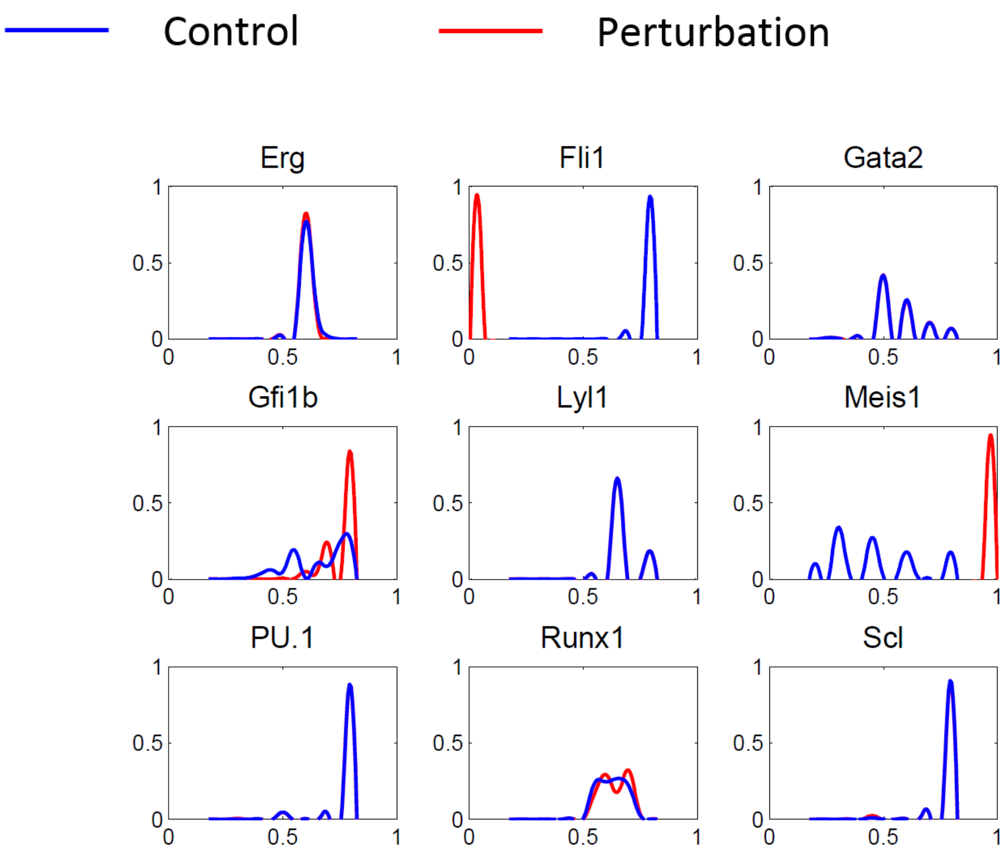

Control
Perturbation
Erg
Fli1
Gata2
Gfi1b
Lyl1
Meis1
PU.1
Runx1
Scl

Top

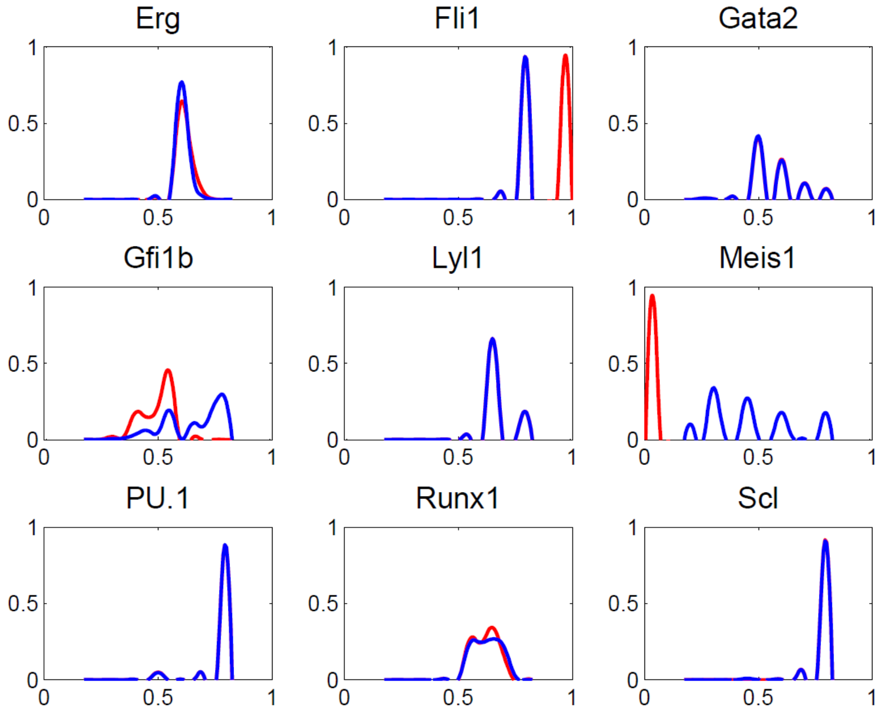

Erg
Fli1
Gata2
Gfi1b
Lyl1
Meis1
PU.1
Runx1
Scl

Top

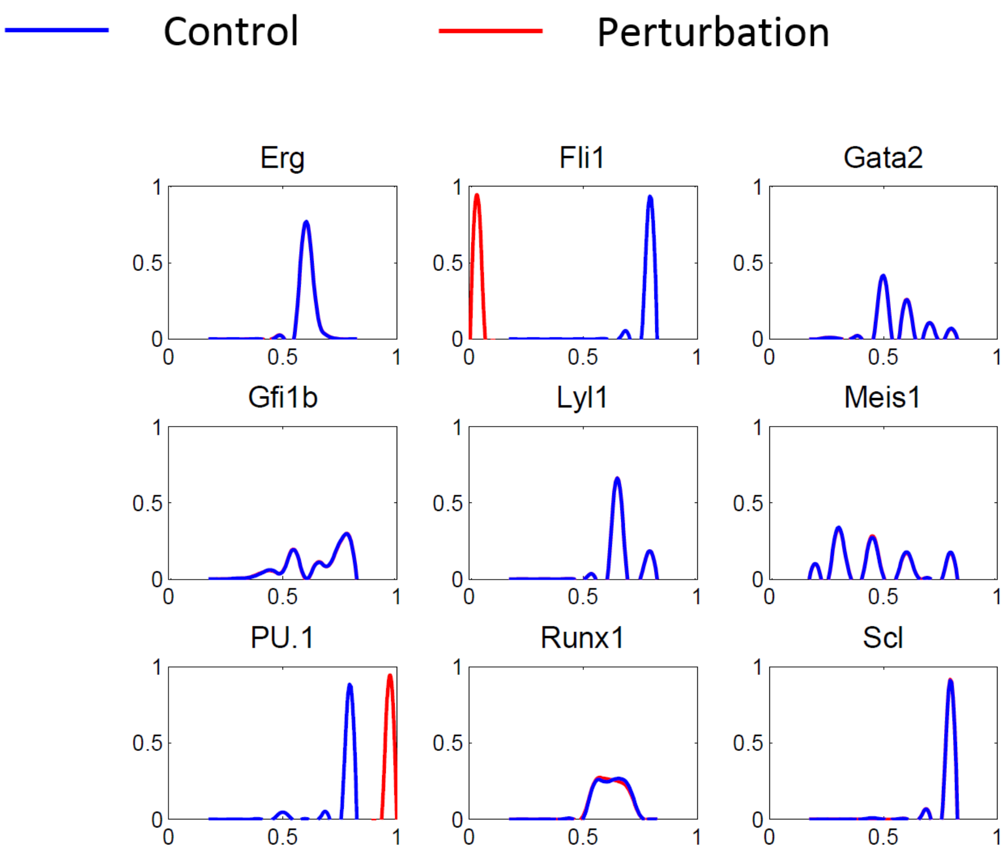
Control
Perturbation
Erg
Fli1
Gata2
Gfi1b
Lyl1
Meis1
PU.1
Runx1
Scl

Top

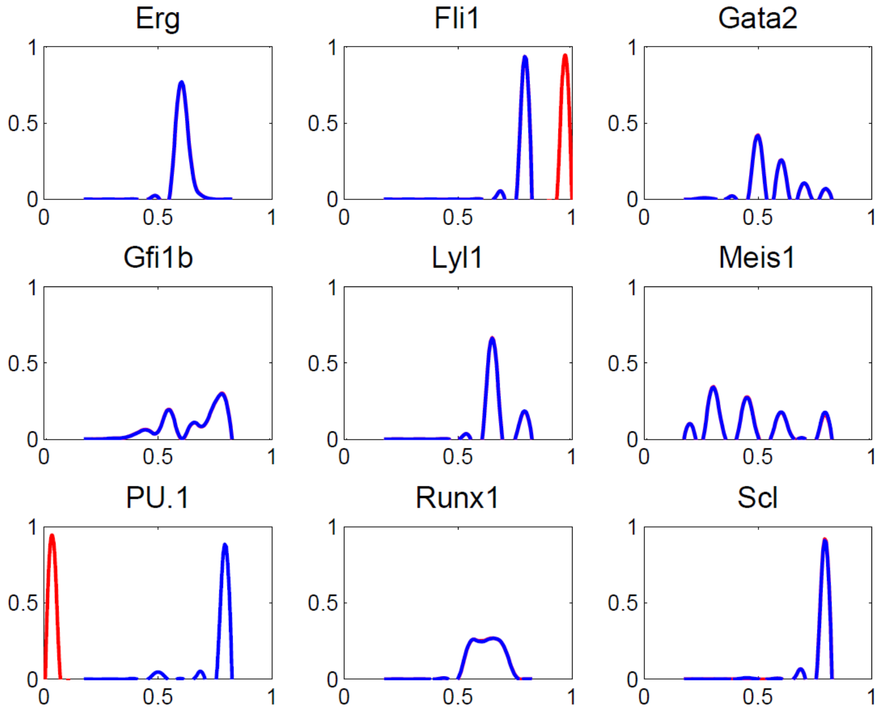
Erg
Fli1
Gata2
Gfi1b
Lyl1
Meis1
PU.1
Runx1
Scl

Top

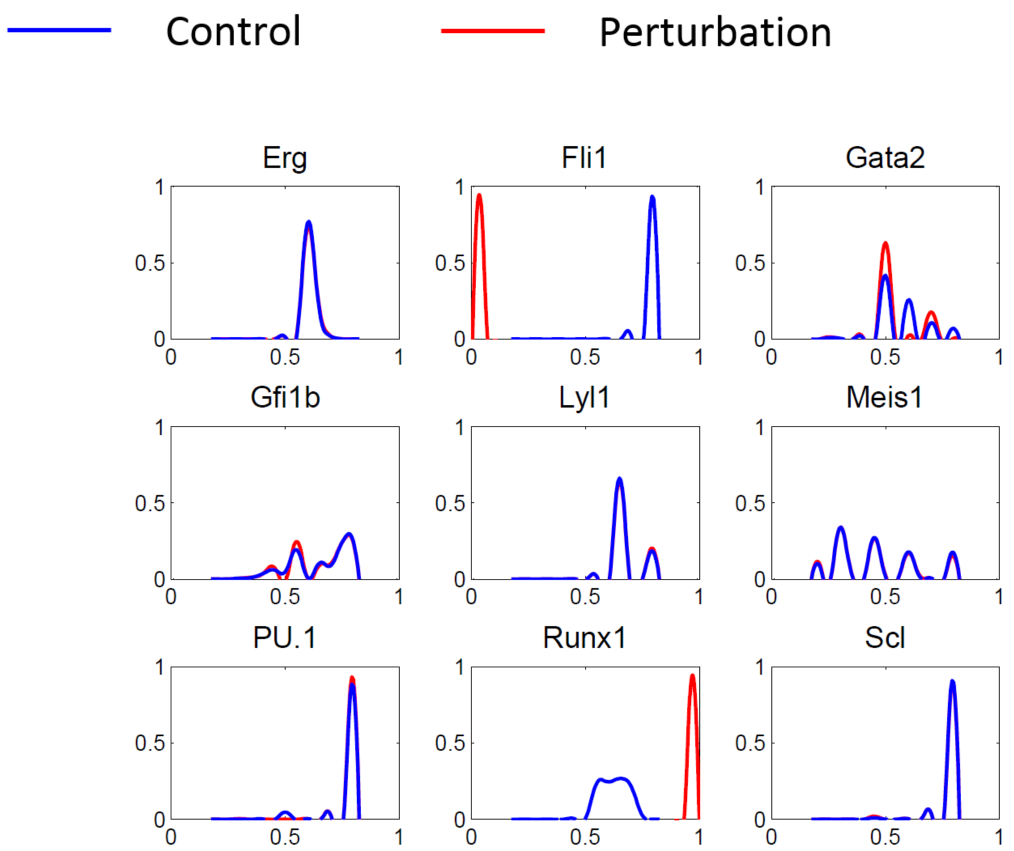
Control
Perturbation
Erg
Fli1
Gata2
Gfi1b
Lyl1
Meis1
PU.1
Runx1
Scl
0
0.5
1

Top

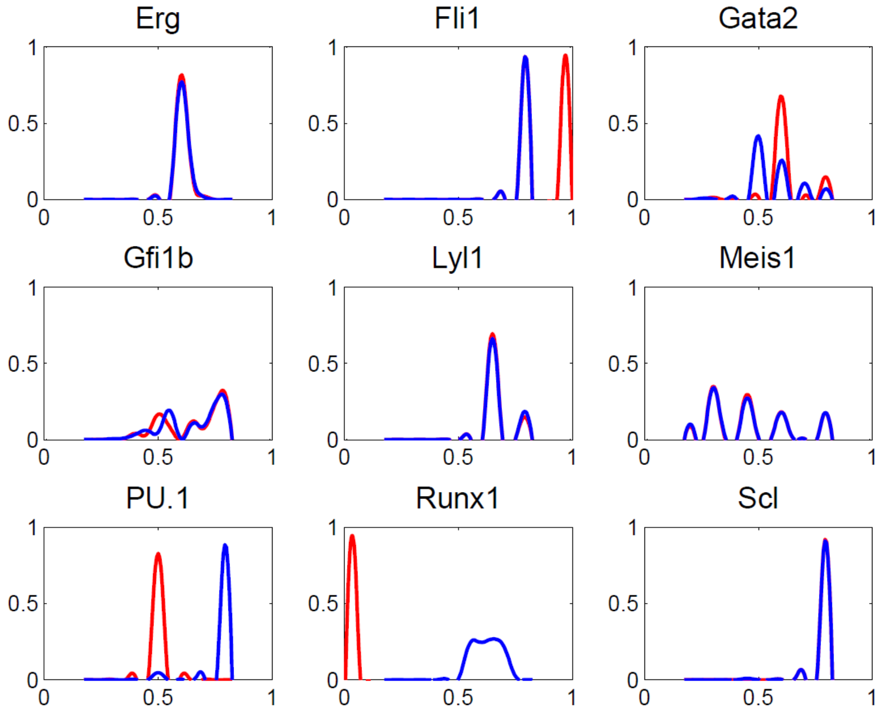
Erg
Fli1
Gata2
Gfi1b
Lyl1
Meis1
PU.1
Runx1
Scl
0
0.5
1

Top

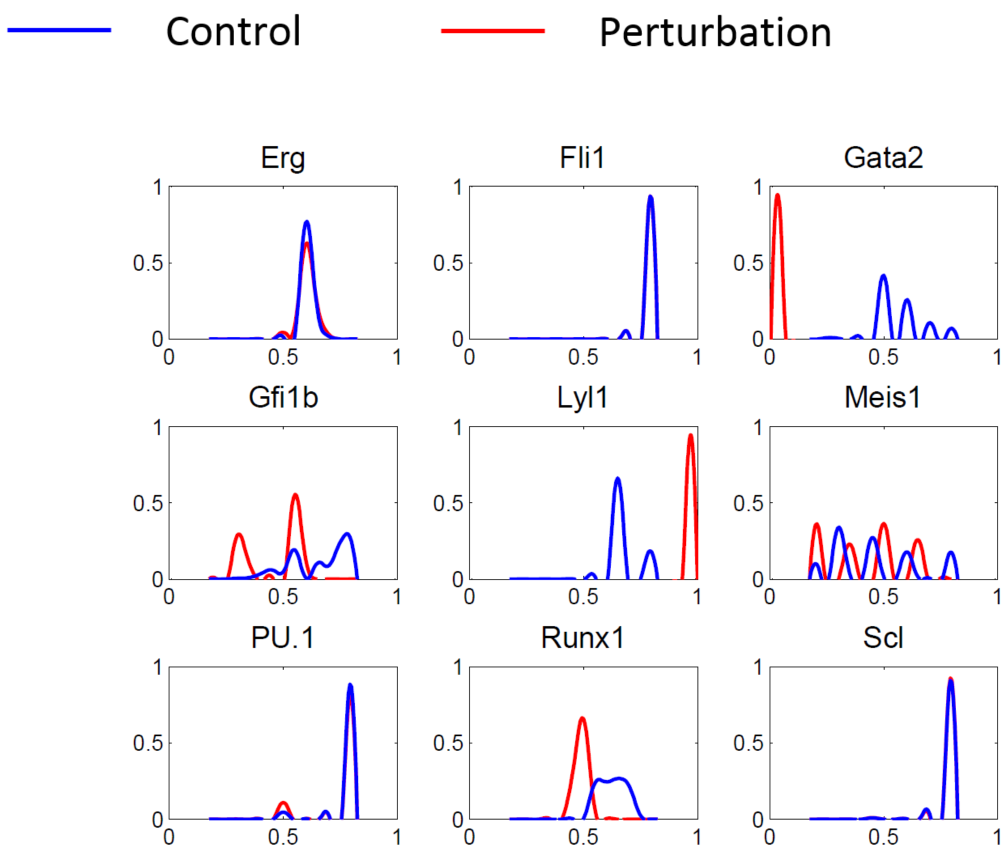
Control
Perturbation
Erg
Fli1
Gata2
Gfi1b
Lyl1
Meis1
PU.1
Runx1
Scl

Top

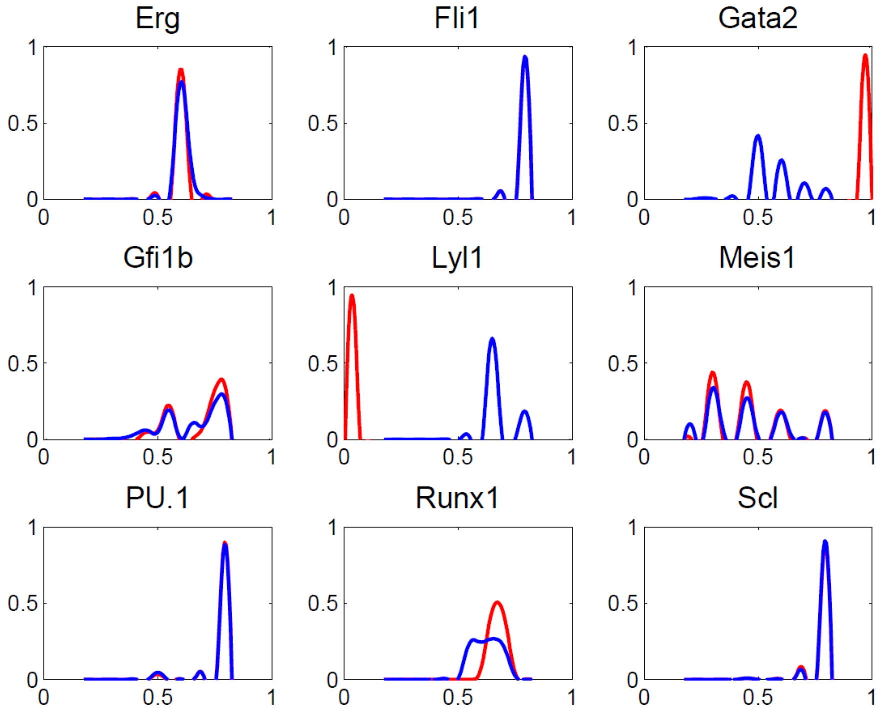
Erg
Fli1
Gata2
Gfi1b
Lyl1
Meis1
PU.1
Runx1
Scl

Top

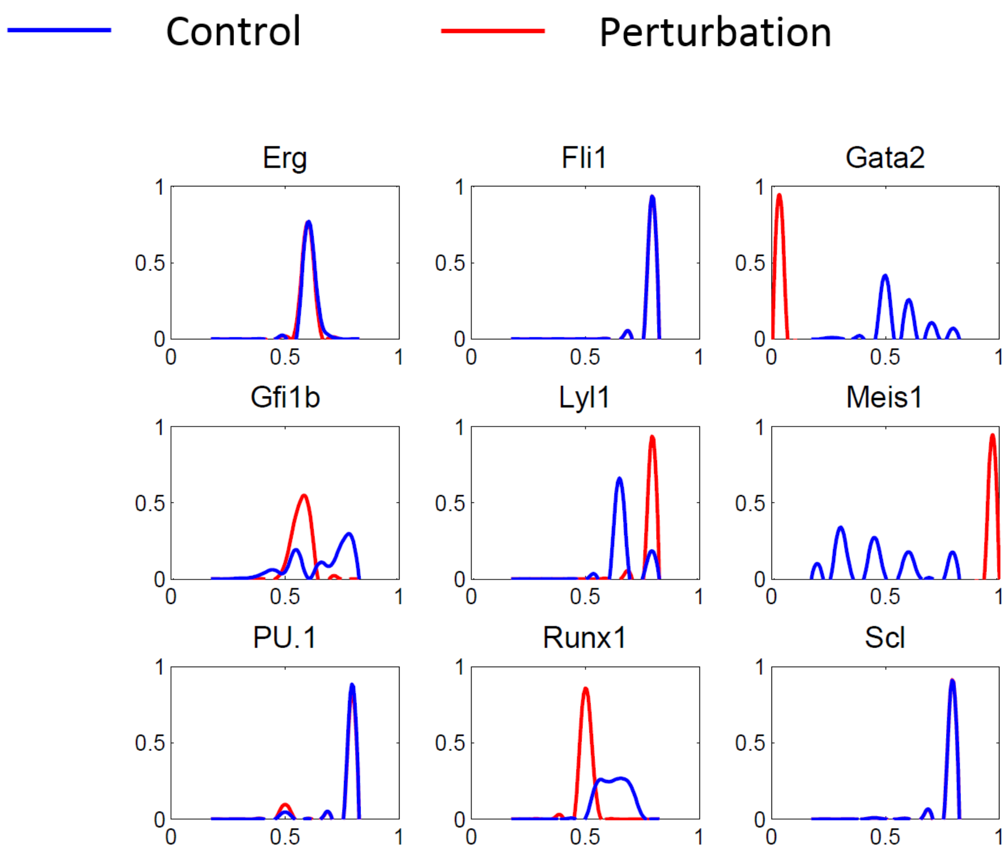
Control
Perturbation
Erg
Fli1
Gata2
Gfi1b
Lyl1
Meis1
PU.1
Runx1
Scl

Top

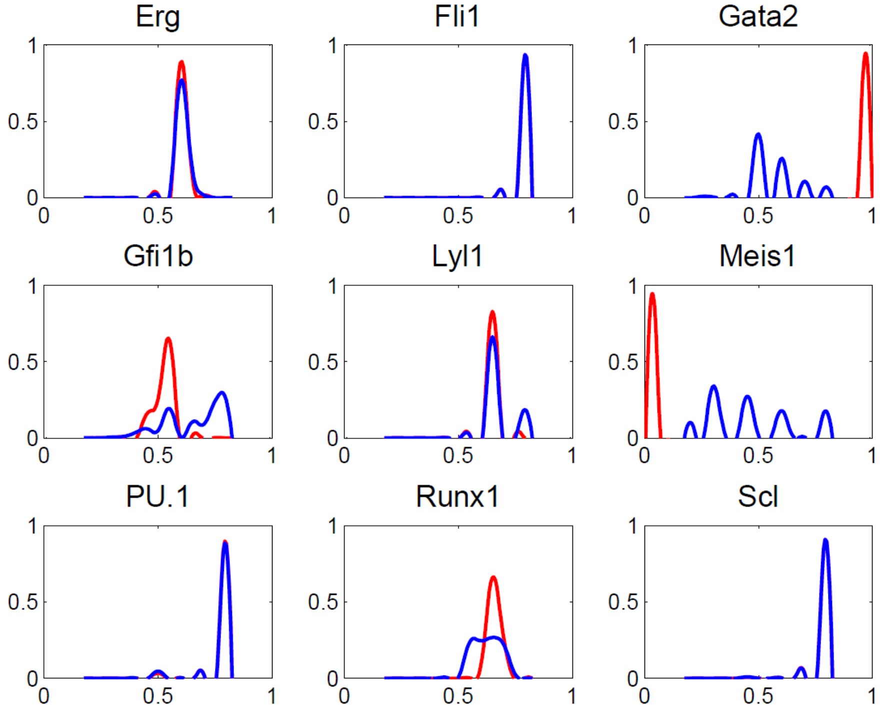
Erg
Fli1
Gata2
Gfi1b
Lyl1
Meis1
PU.1
Runx1
Scl

Top

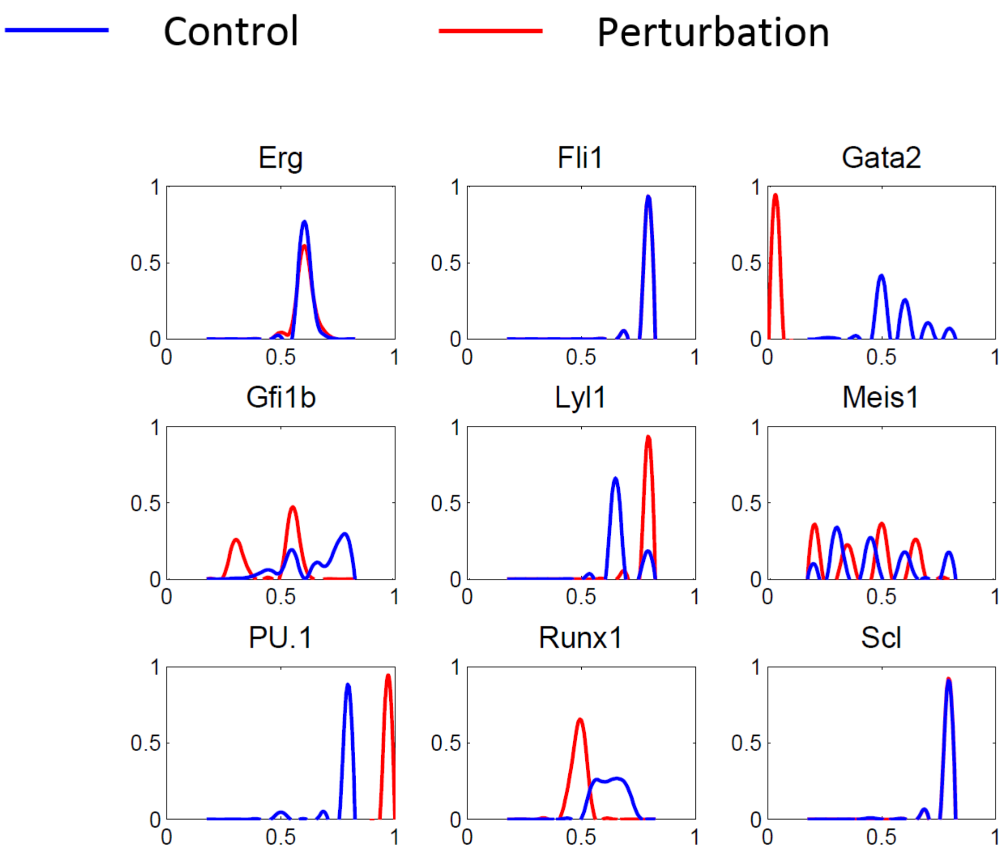
Control
Perturbation
Erg
Fli1
Gata2
Gfi1b
Lyl1
Meis1
PU.1
Runx1
Scl

Top

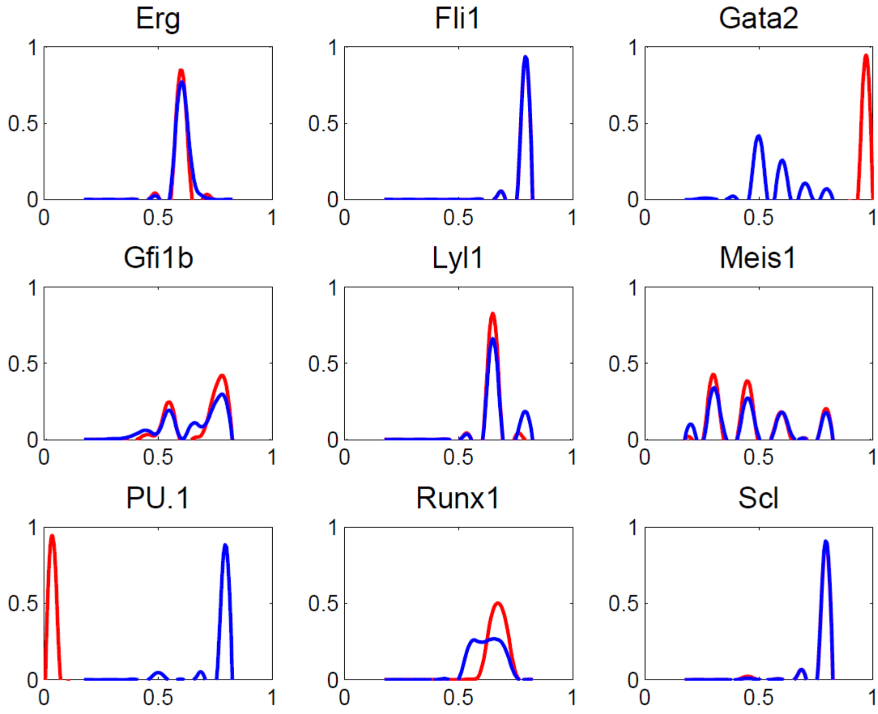
Erg
Fli1
Gata2
Gfi1b
Lyl1
Meis1
PU.1
Runx1
Scl

Top

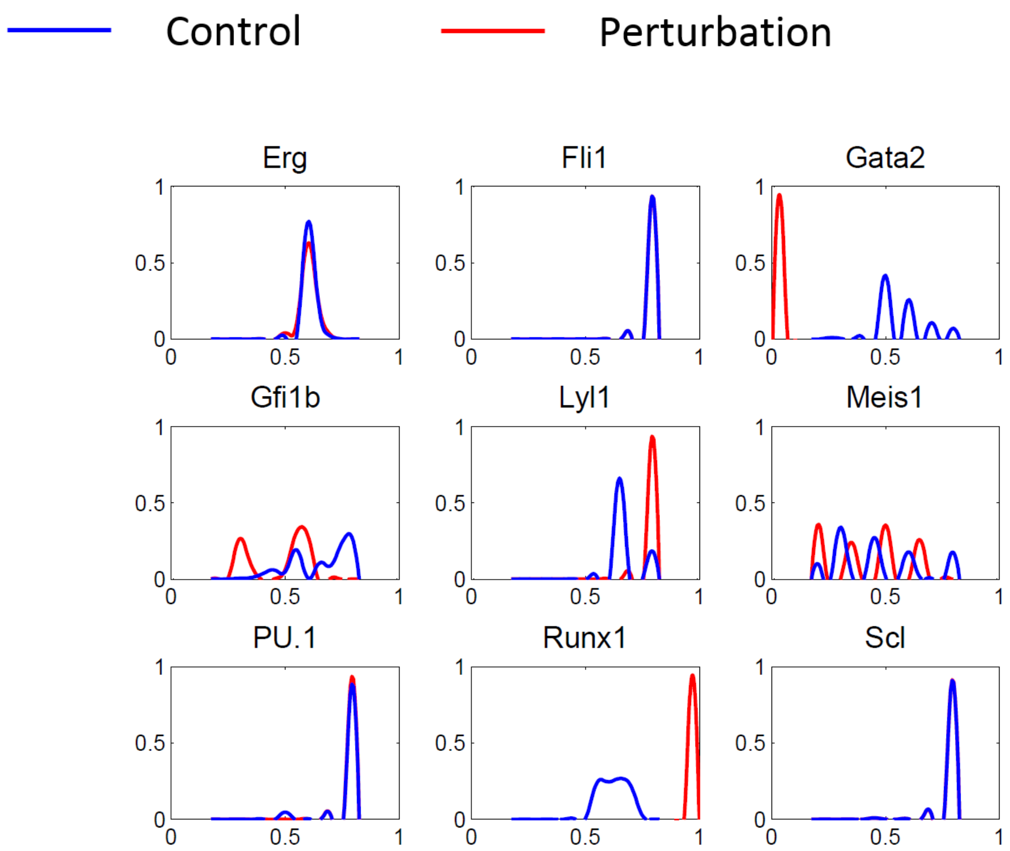
Control
Perturbation
Erg
Fli1
Gata2
Gfi1b
Lyl1
Meis1
PU.1
Runx1
Scl

Top

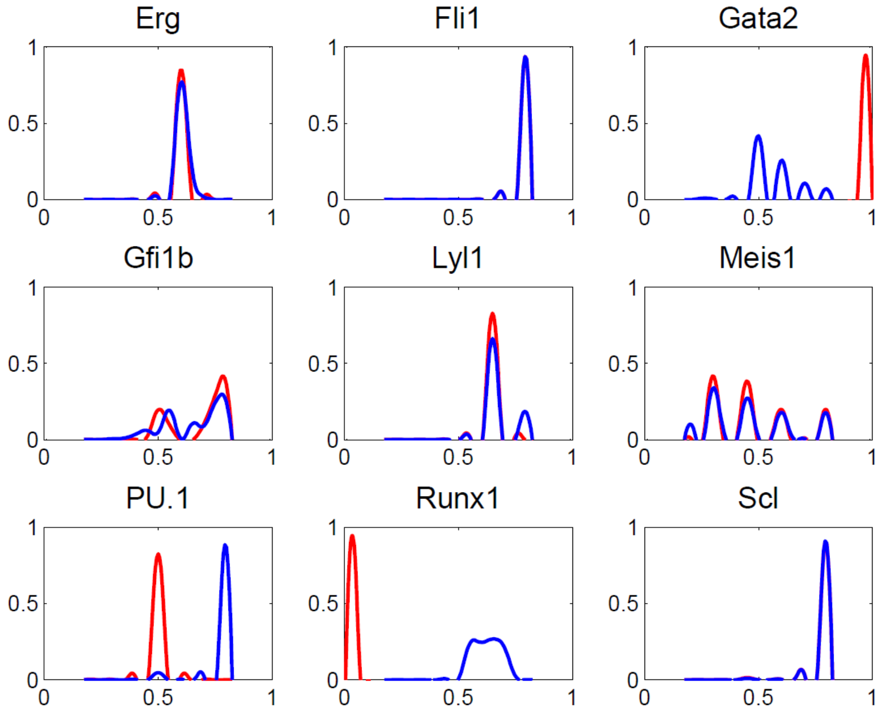
Erg
Fli1
Gata2
Gfi1b
Lyl1
Meis1
PU.1
Runx1
Scl

Top

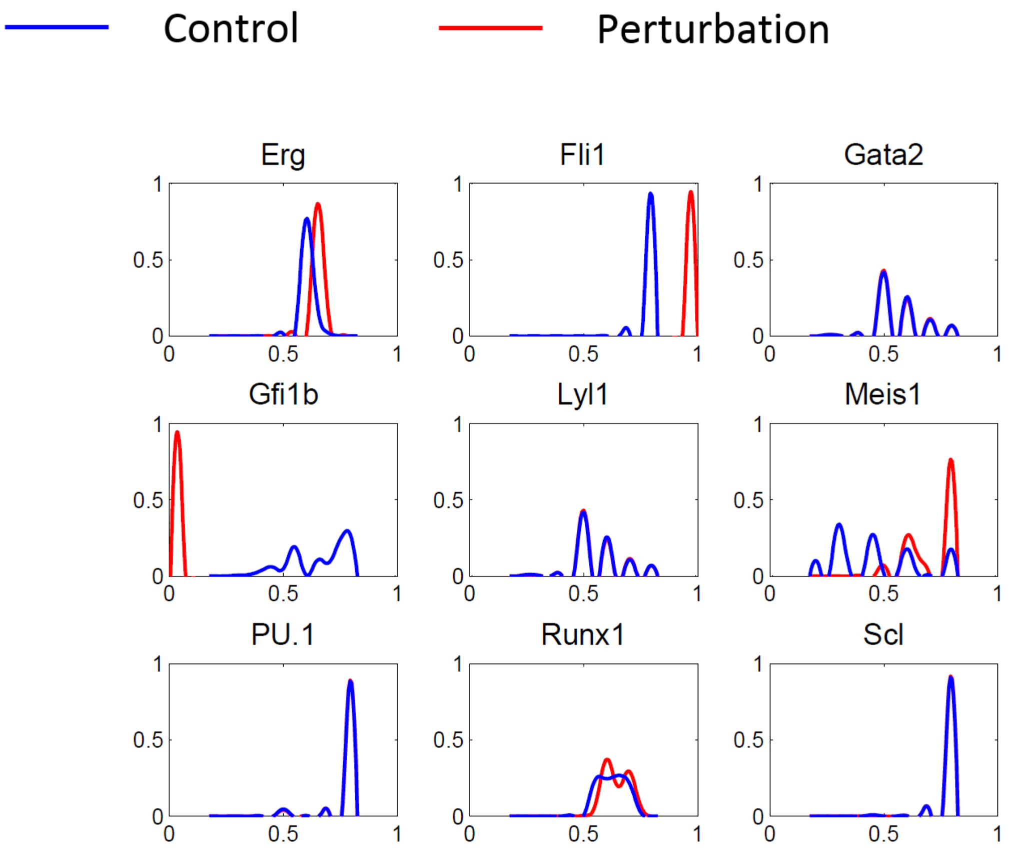
Control
Perturbation
Erg
Fli1
Gata2
Gfi1b
Lyl1
Meis1
PU.1
Runx1
Scl

Top

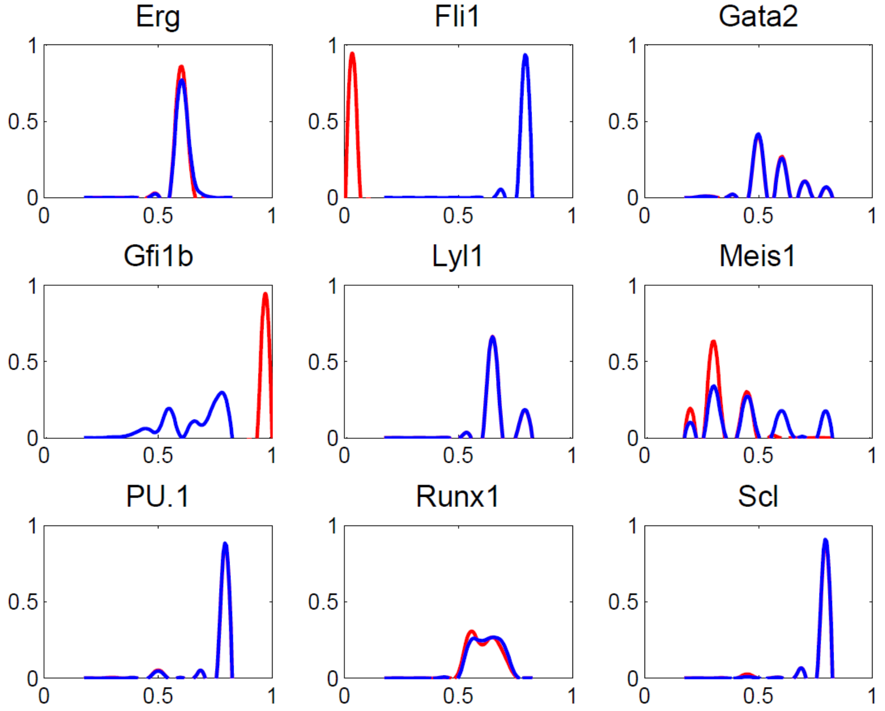
Erg
Fli1
Gata2
Gfi1b
Lyl1
Meis1
PU.1
Runx1
Scl

Top

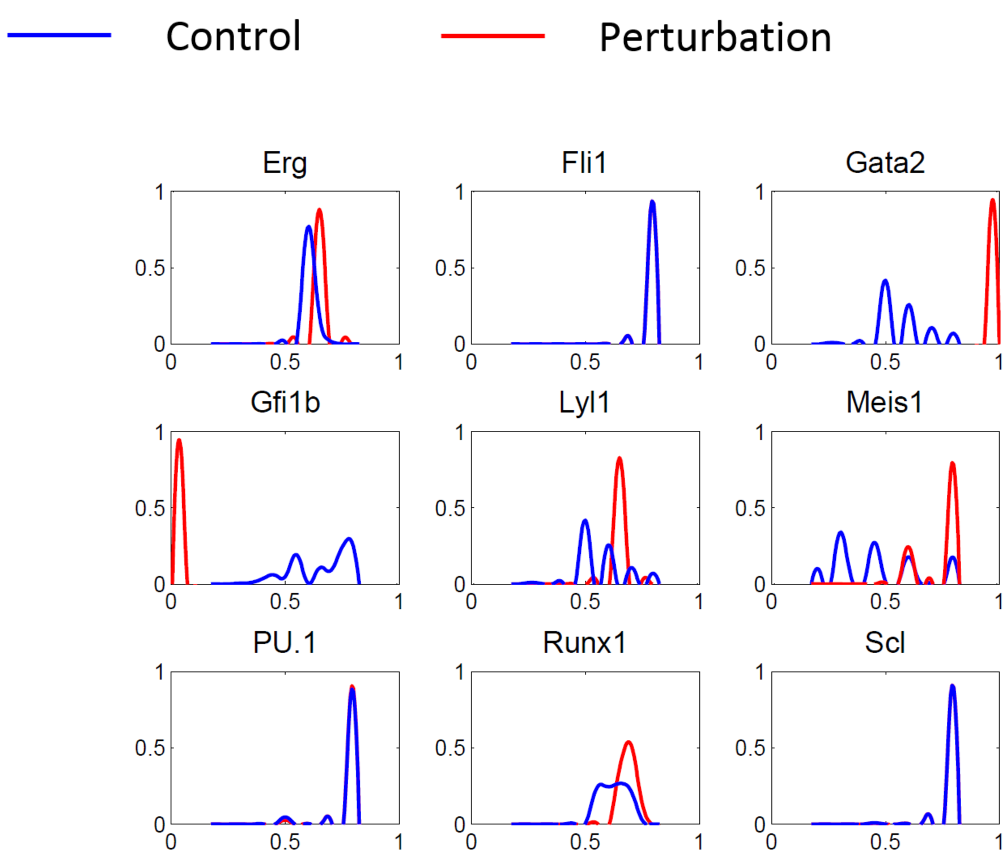
Control
Perturbation
Erg
Fli1
Gata2
Gfi1b
Lyl1
Meis1
PU.1
Runx1
Scl

Top

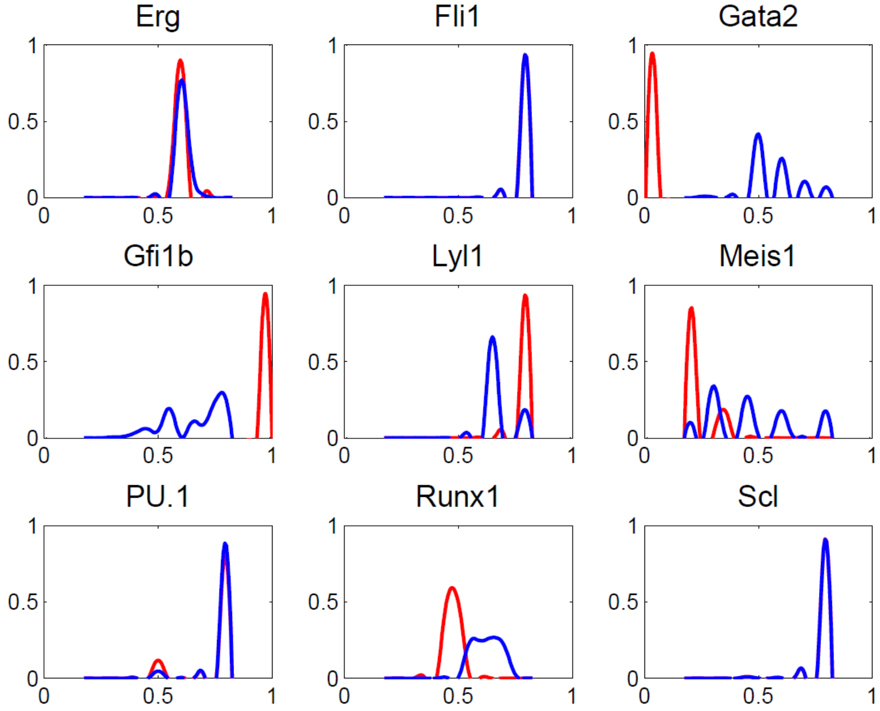
Erg
Fli1
Gata2
Gfi1b
Lyl1
Meis1
PU.1
Runx1
Scl

Top

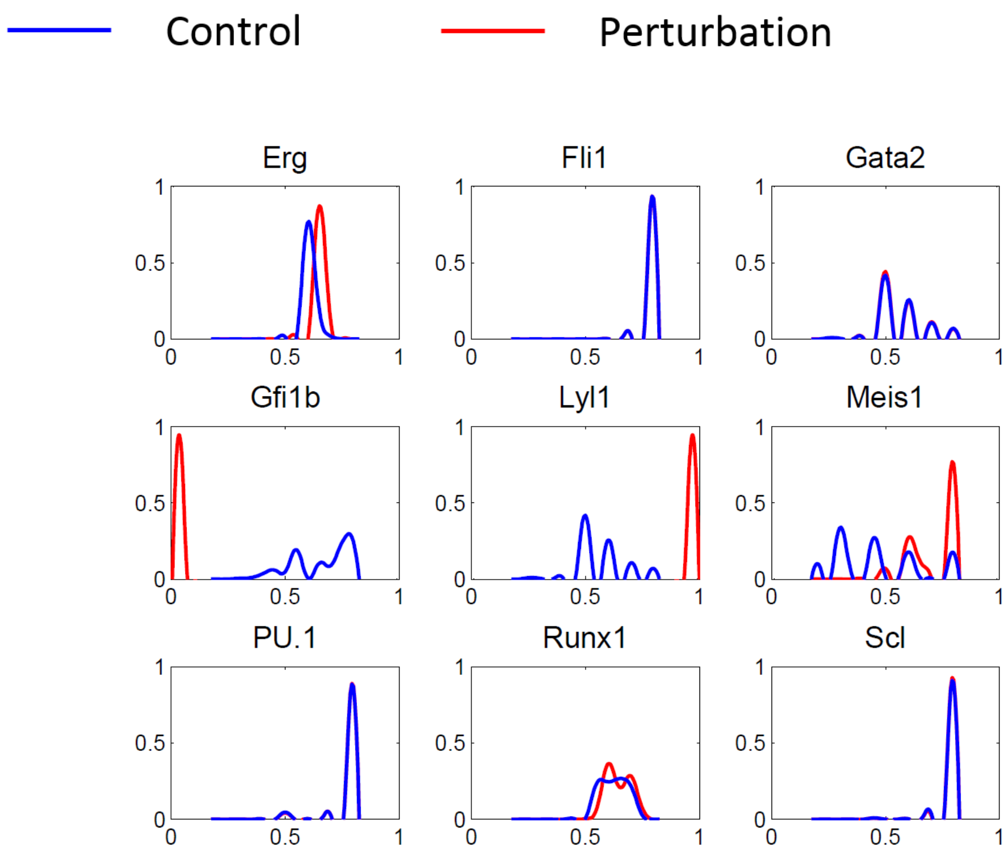
Control
Perturbation
Erg
Fli1
Gata2
Gfi1b
Lyl1
Meis1
PU.1
Runx1
Scl

Top

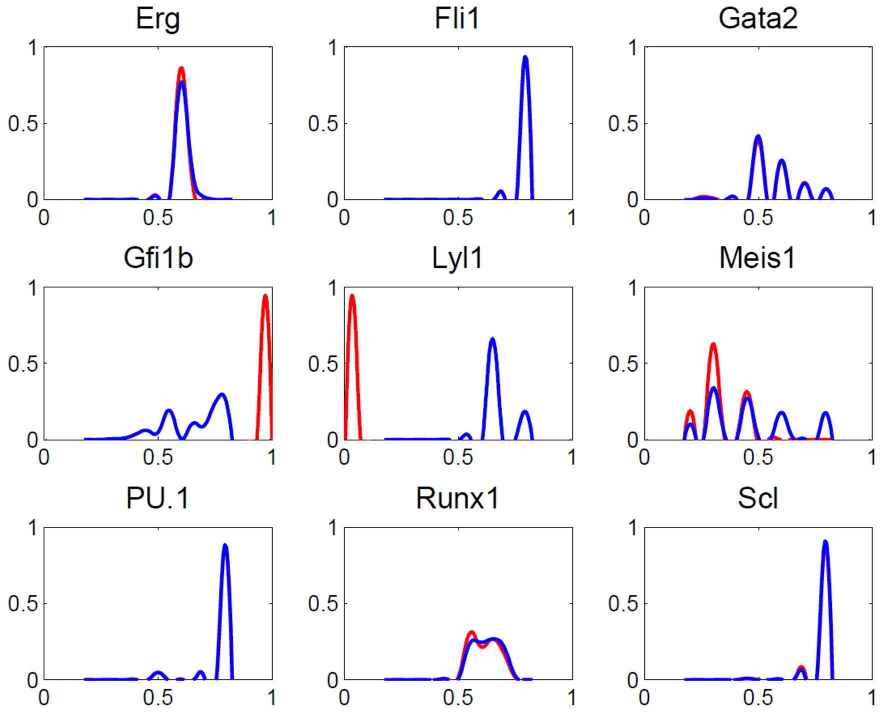
Erg
Fli1
Gata2
Gfi1b
Lyl1
Meis1
PU.1
Runx1
Scl

Top

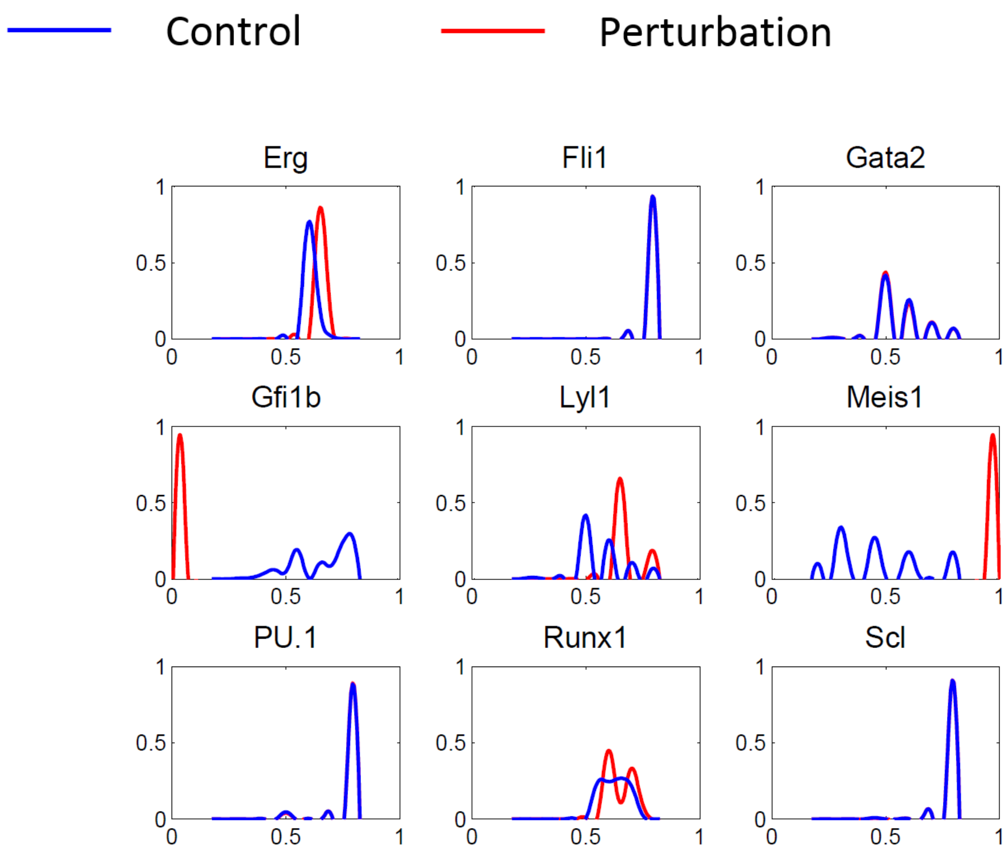
Control
Perturbation
Erg
Fli1
Gata2
Gfi1b
Lyl1
Meis1
PU.1
Runx1
Scl

Top

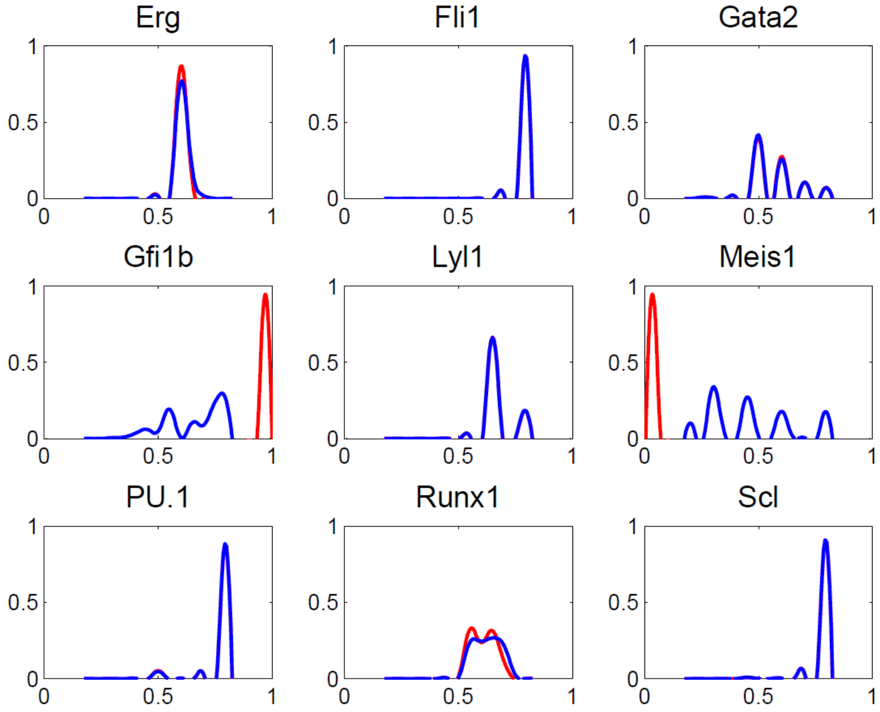
Erg
Fli1
Gata2
Gfi1b
Lyl1
Meis1
PU.1
Runx1
Scl

Top

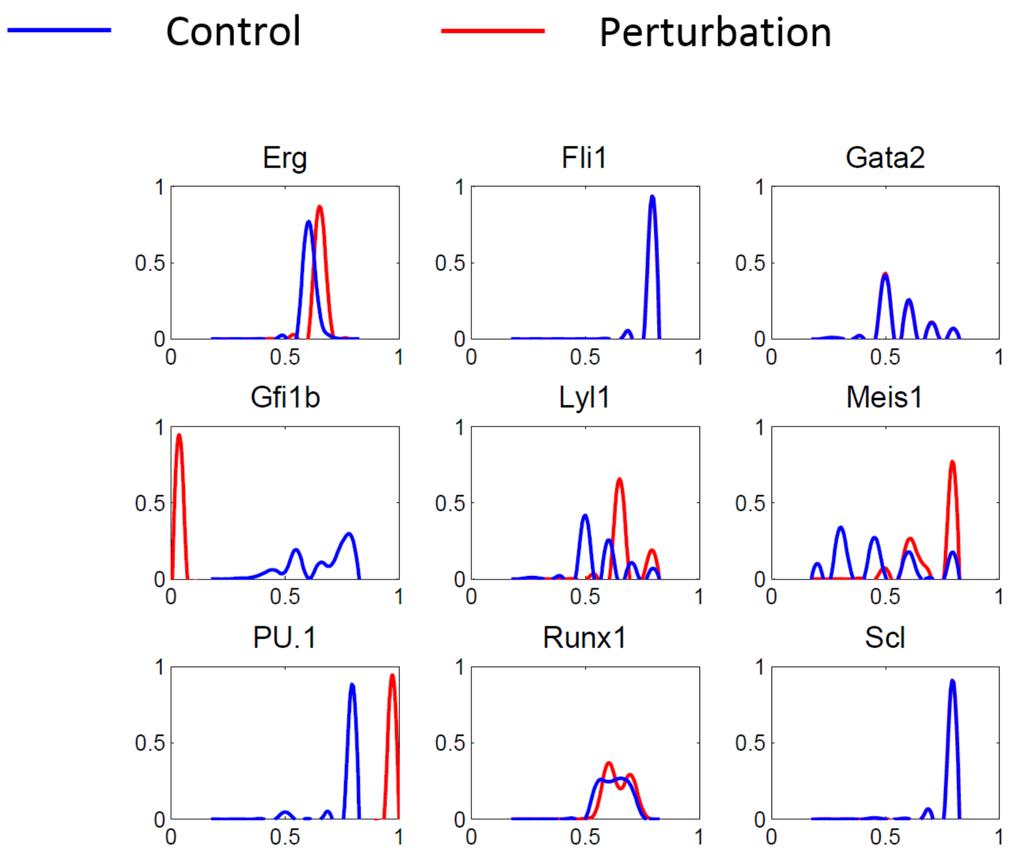
Control
Perturbation
Erg
Fli1
Gata2
Gfi1b
Lyl1
Meis1
PU.1
Runx1
Scl

Top

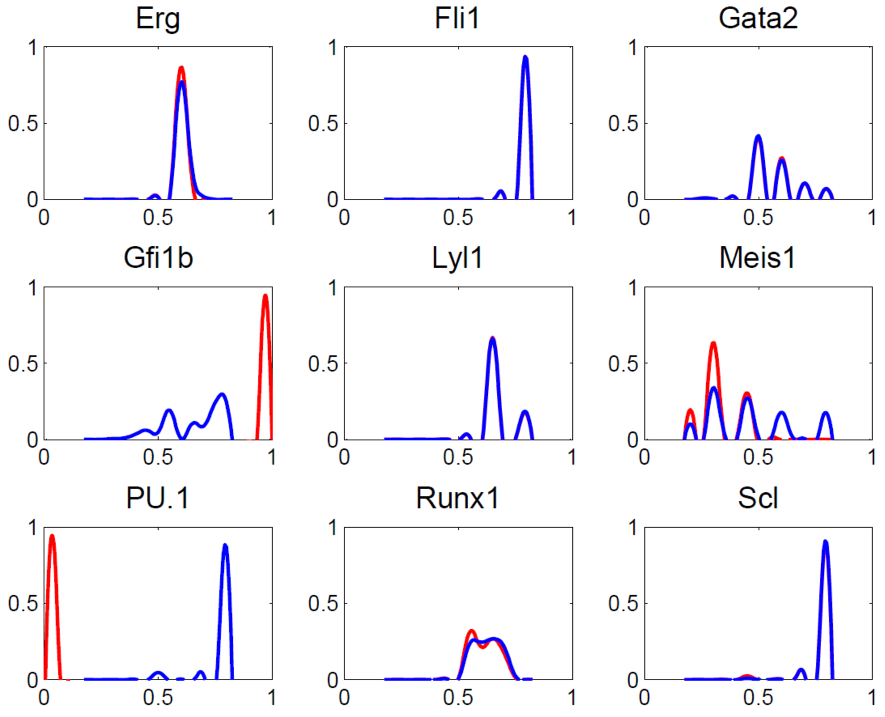
Erg
Fli1
Gata2
Gfi1b
Lyl1
Meis1
PU.1
Runx1
Scl

Top

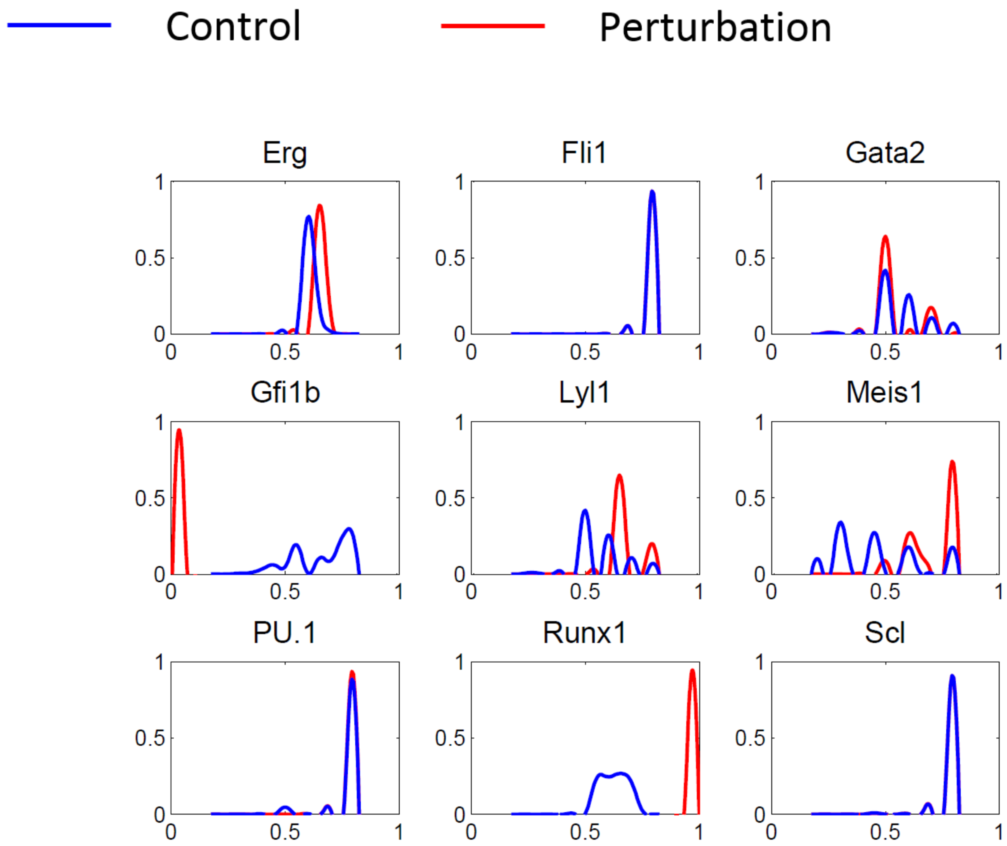
Control
Perturbation
Erg
Fli1
Gata2
Gfi1b
Lyl1
Meis1
PU.1
Runx1
Scl
0
0.5
1

Top

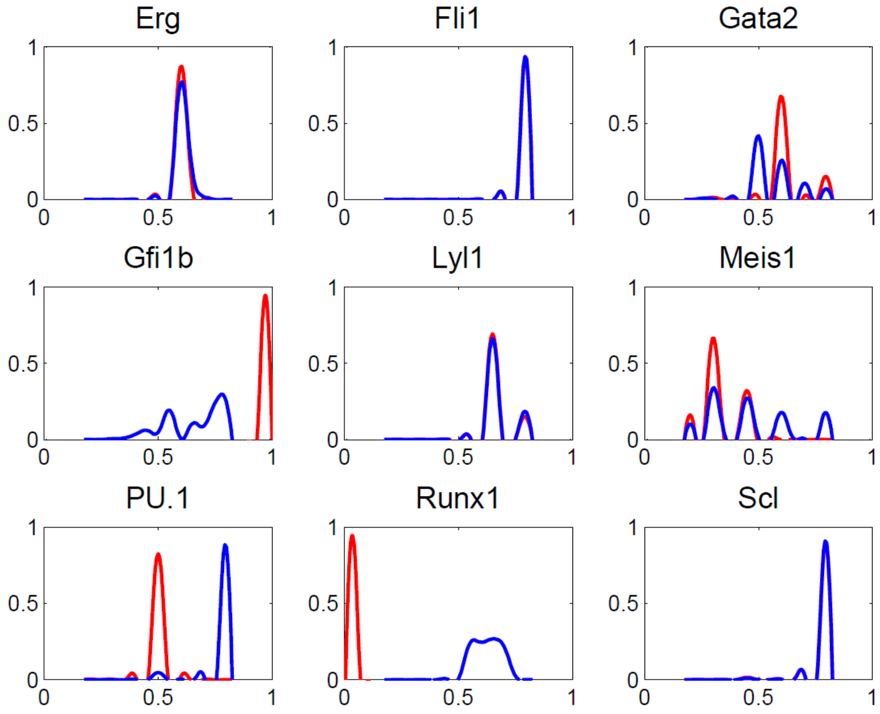
Erg
Fli1
Gata2
Gfi1b
Lyl1
Meis1
PU.1
Runx1
Scl
0
0.5
1

Top

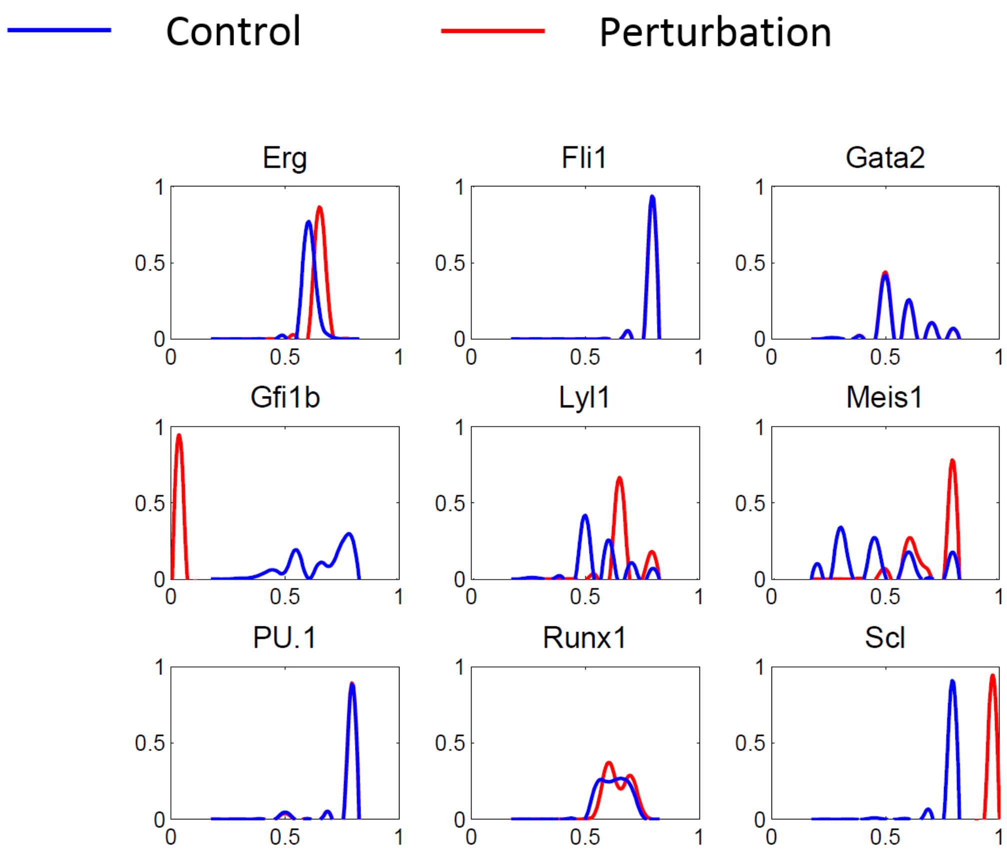
Control
Perturbation
Erg
Fli1
Gata2
Gfi1b
Lyl1
Meis1
PU.1
Runx1
Scl

Top

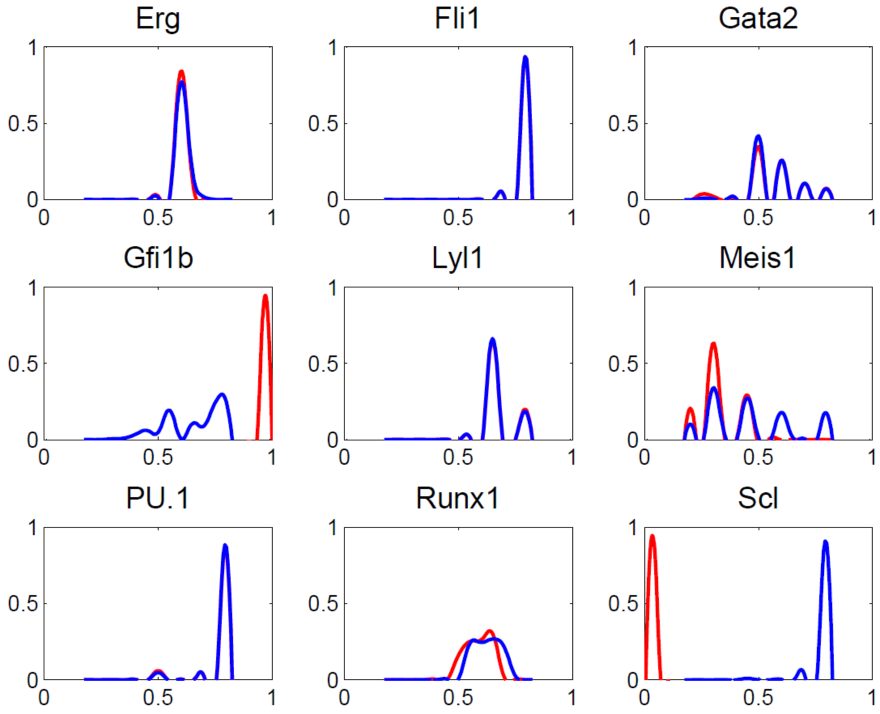
Erg
Fli1
Gata2
Gfi1b
Lyl1
Meis1
PU.1
Runx1
Scl

Top

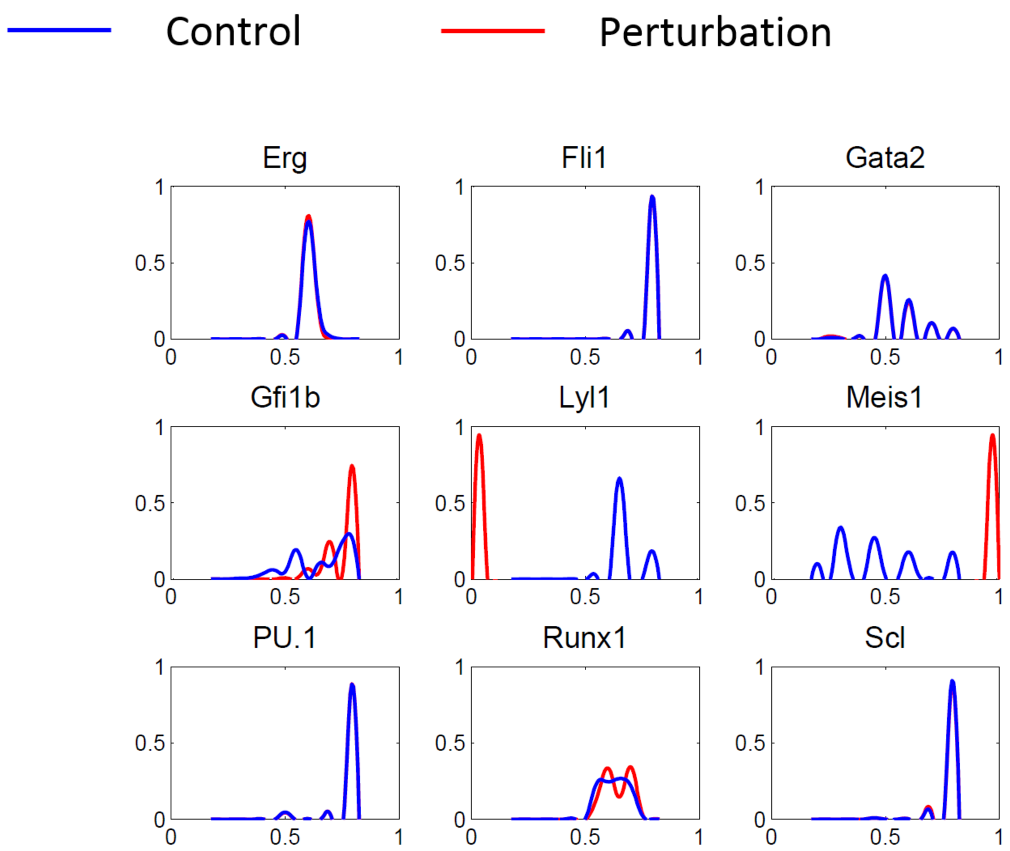
Control
Perturbation
Erg
Fli1
Gata2
Gfi1b
Lyl1
Meis1
PU.1
Runx1
Scl

Top

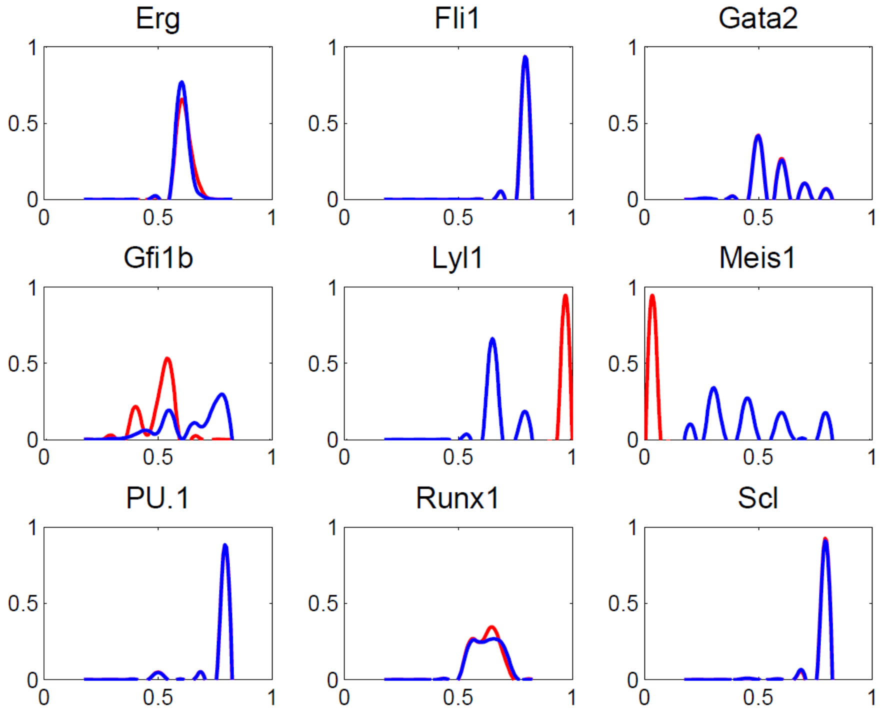
Erg
Fli1
Gata2
Gfi1b
Lyl1
Meis1
PU.1
Runx1
Scl

Top

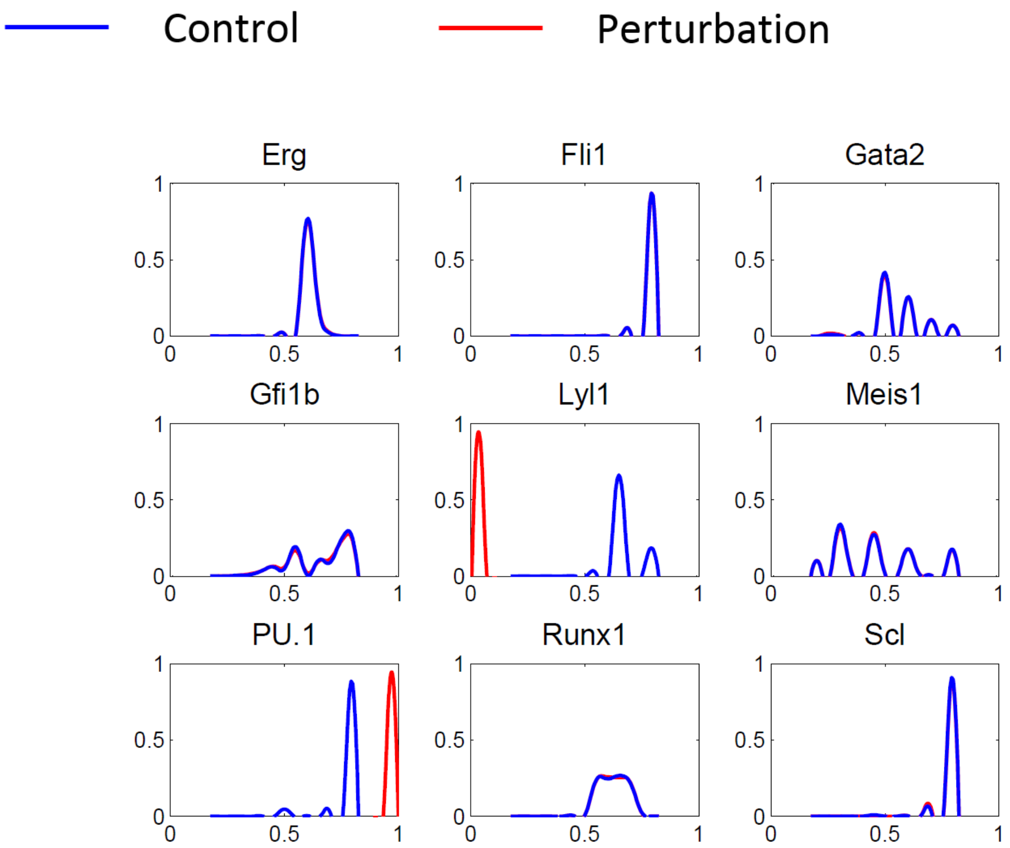
Control
Perturbation
Erg
Fli1
Gata2
Gfi1b
Lyl1
Meis1
PU.1
Runx1
Scl

Top

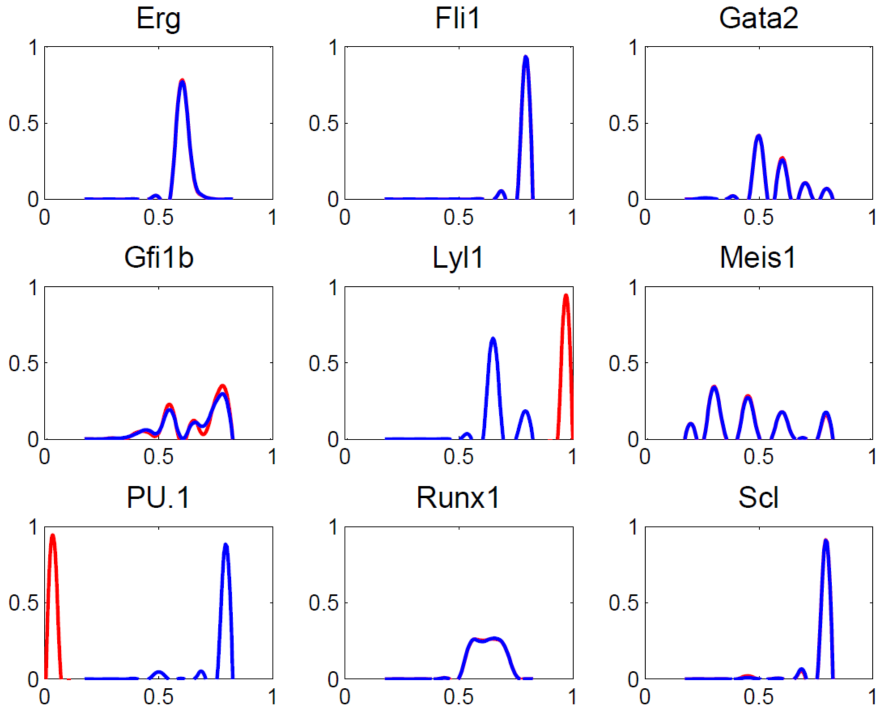
Erg
Fli1
Gata2
Gfi1b
Lyl1
Meis1
PU.1
Runx1
Scl

Top

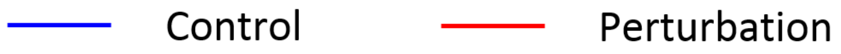
Control
Perturbation

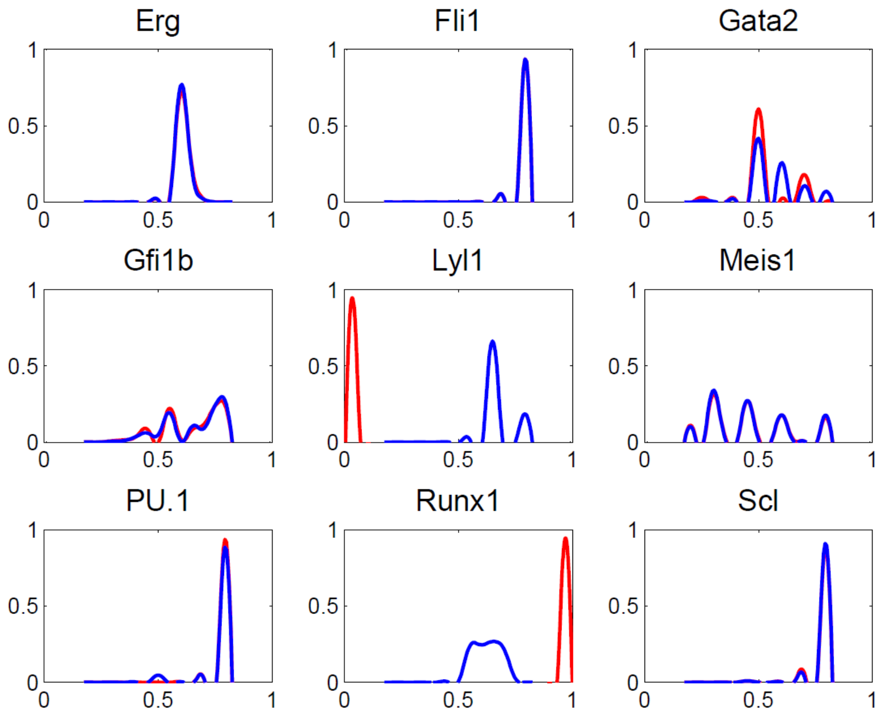
Erg
Fli1
Gata2
Gfi1b
Lyl1
Meis1
PU.1
Runx1
Scl

Top

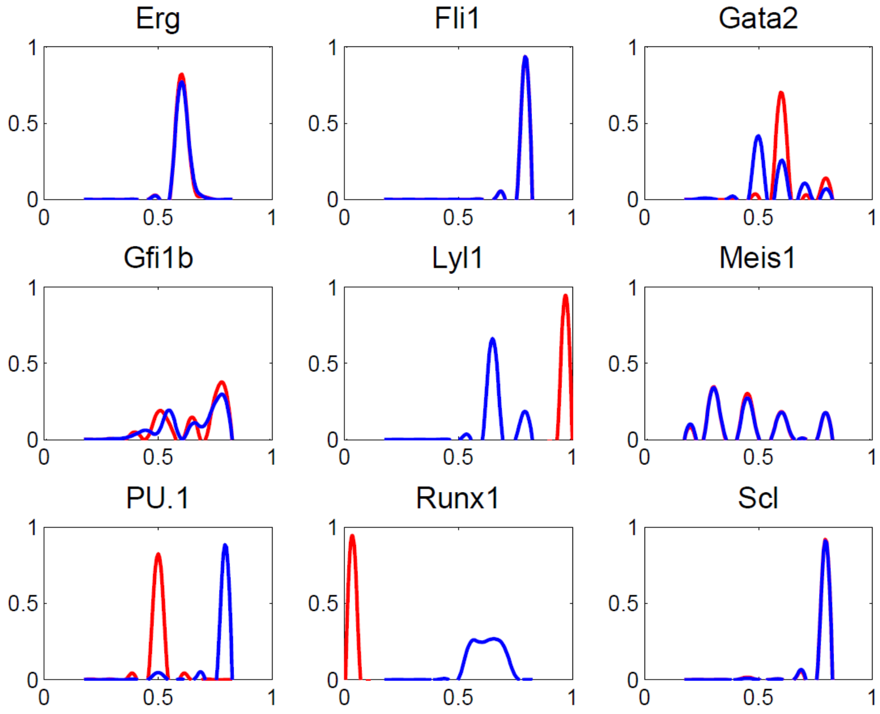
Erg
Fli1
Gata2
Gfi1b
Lyl1
Meis1
PU.1
Runx1
Scl

Top

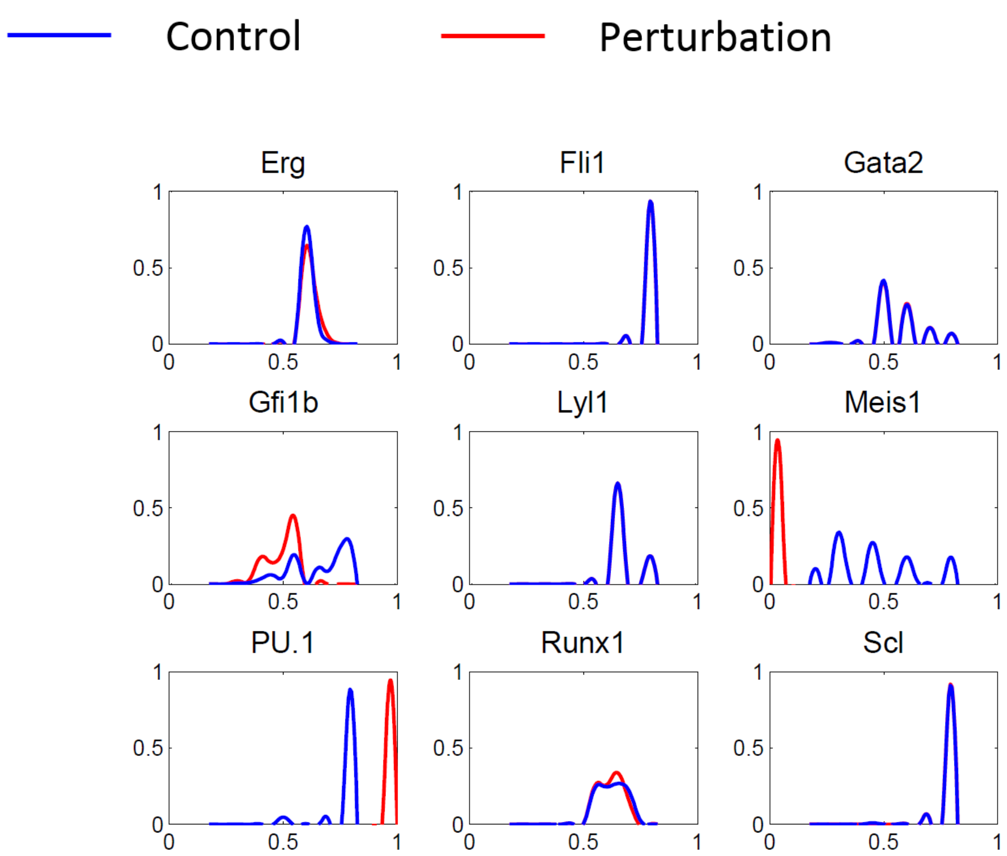
Control
Perturbation
Erg
Fli1
Gata2
Gfi1b
Lyl1
Meis1
PU.1
Runx1
Scl

Top

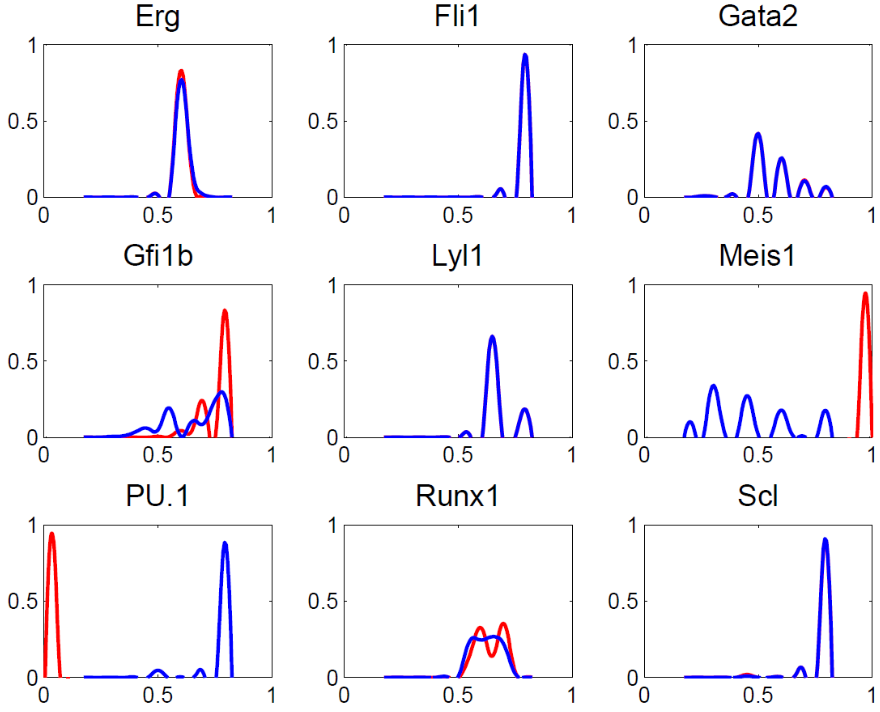
Erg
Fli1
Gata2
Gfi1b
Lyl1
Meis1
PU.1
Runx1
Scl

Top

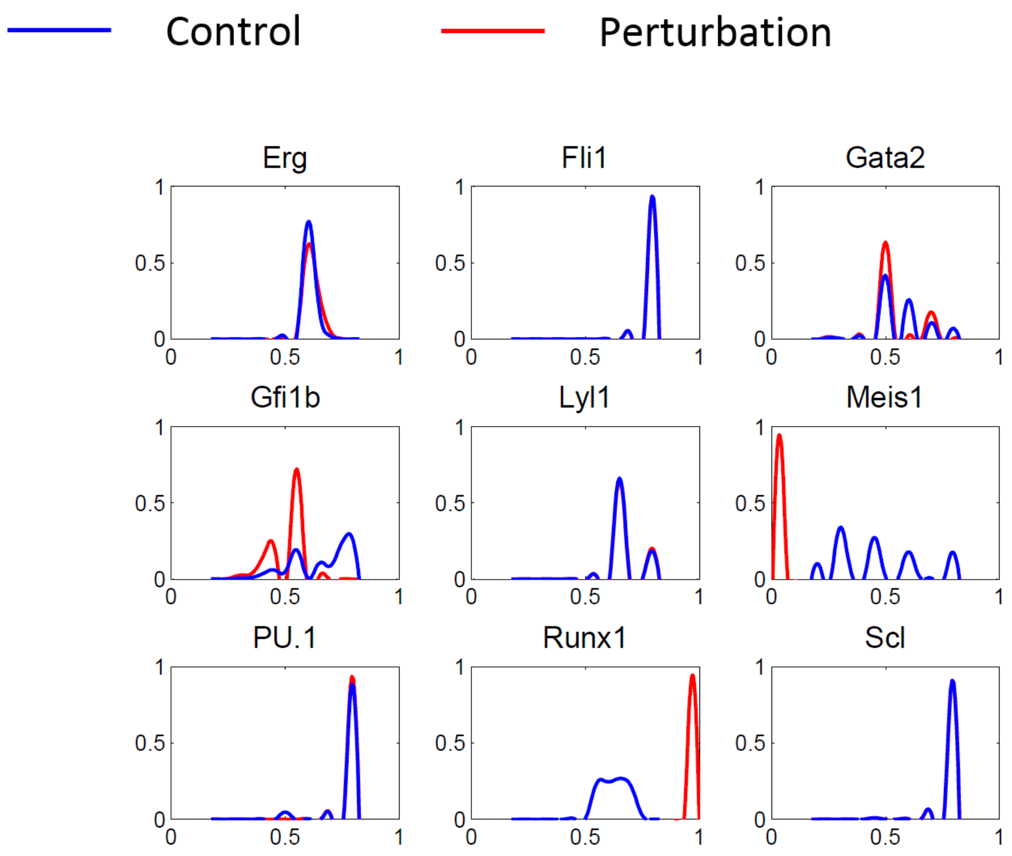
Control
Perturbation
Erg
Fli1
Gata2
Gfi1b
Lyl1
Meis1
PU.1
Runx1
Scl
0
0.5
1

Top

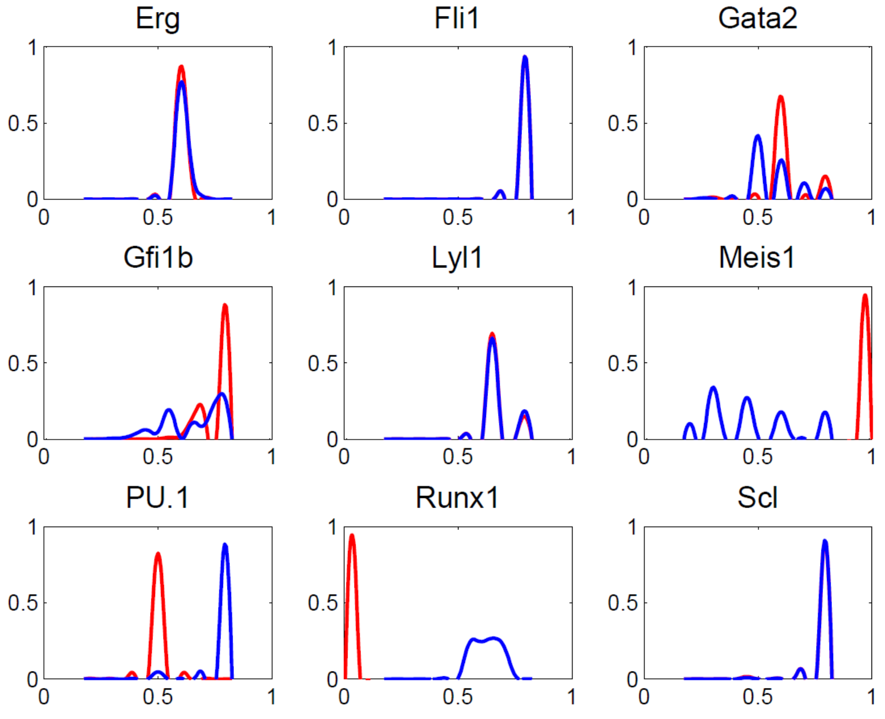
Erg
Fli1
Gata2
Gfi1b
Lyl1
Meis1
PU.1
Runx1
Scl
0
0.5
1

Top

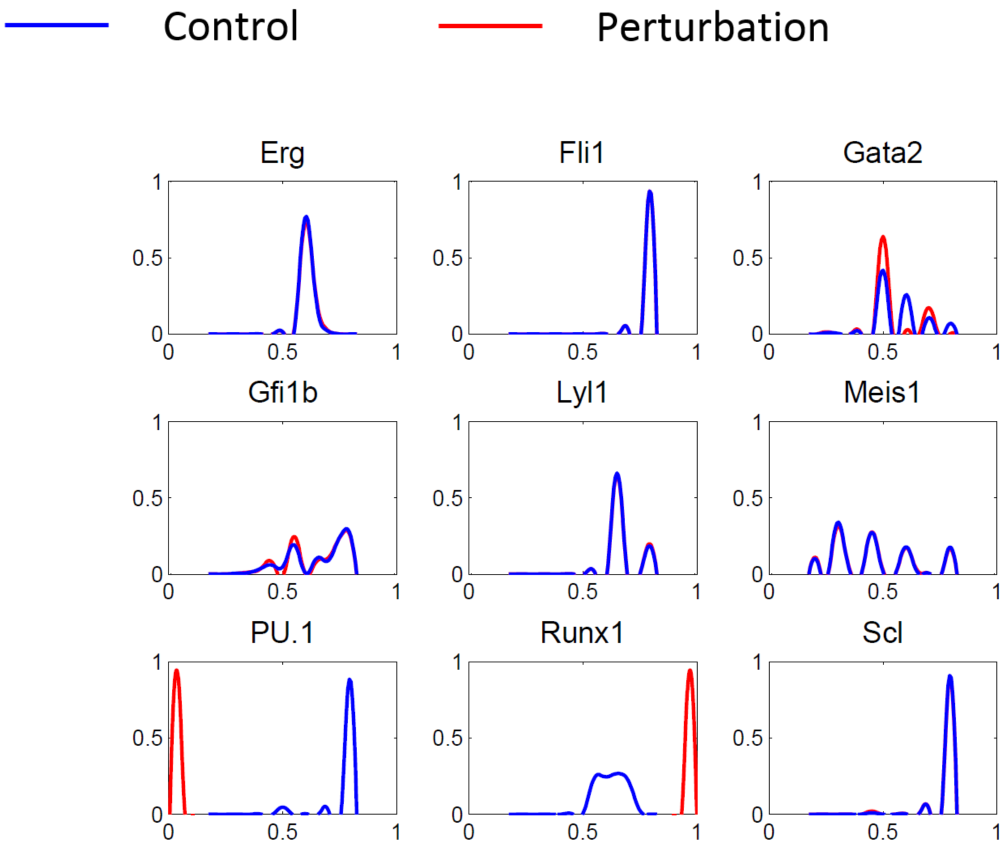
Control
Perturbation
Erg
Fli1
Gata2
Gfi1b
Lyl1
Meis1
PU.1
Runx1
Scl
0
0.5
1

Top

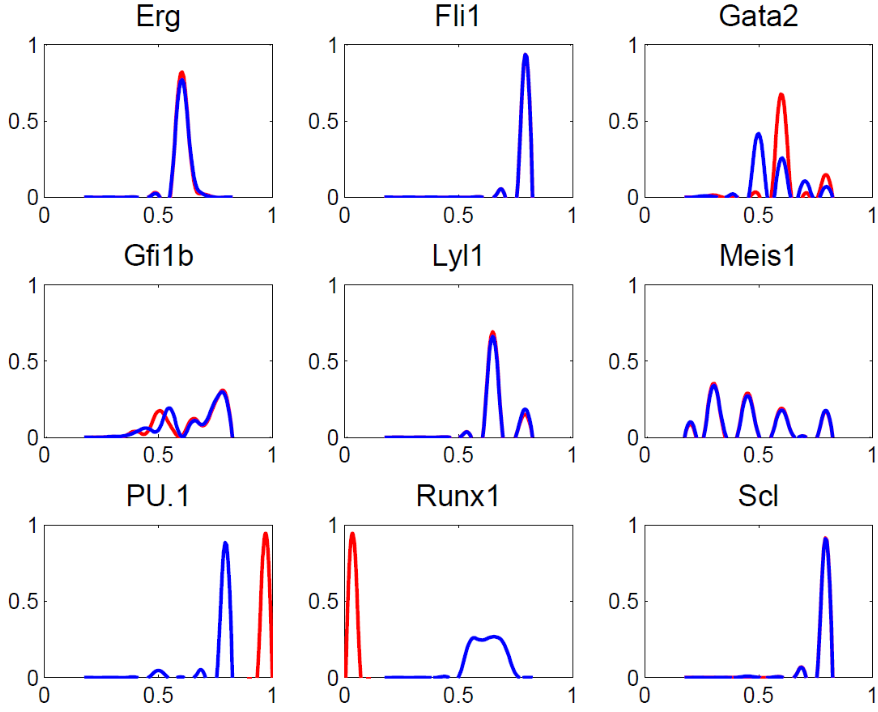
Erg
Fli1
Gata2
Gfi1b
Lyl1
Meis1
PU.1
Runx1
Scl
0
0.5
1

Top

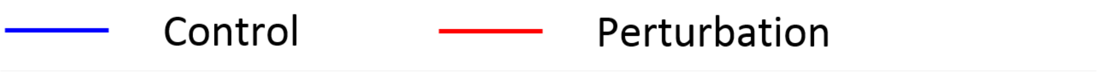
Control
Perturbation

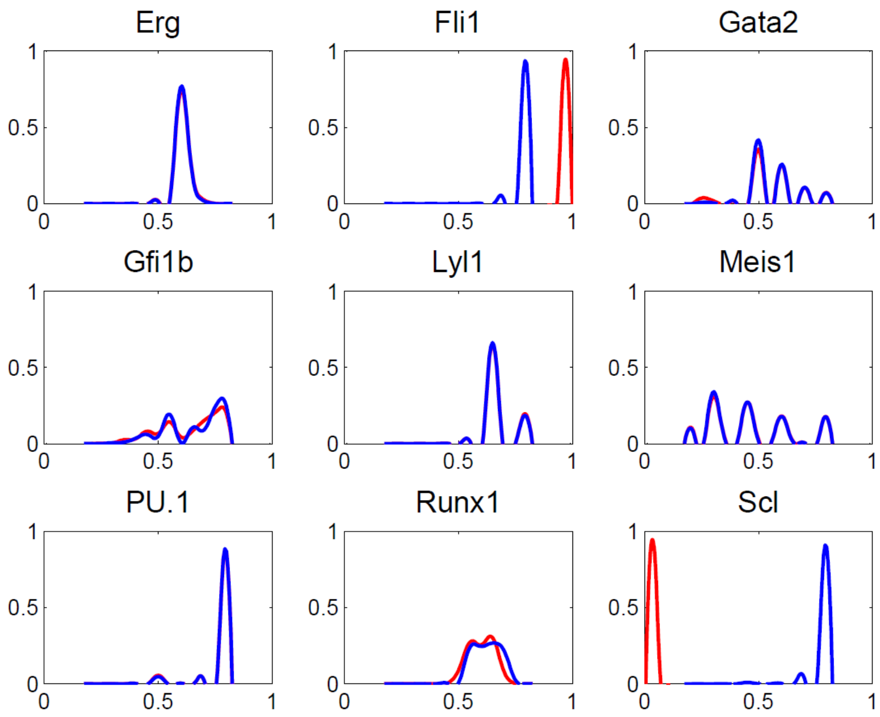
Erg
Fli1
Gata2
Gfi1b
Lyl1
Meis1
PU.1
Runx1
Scl

Top

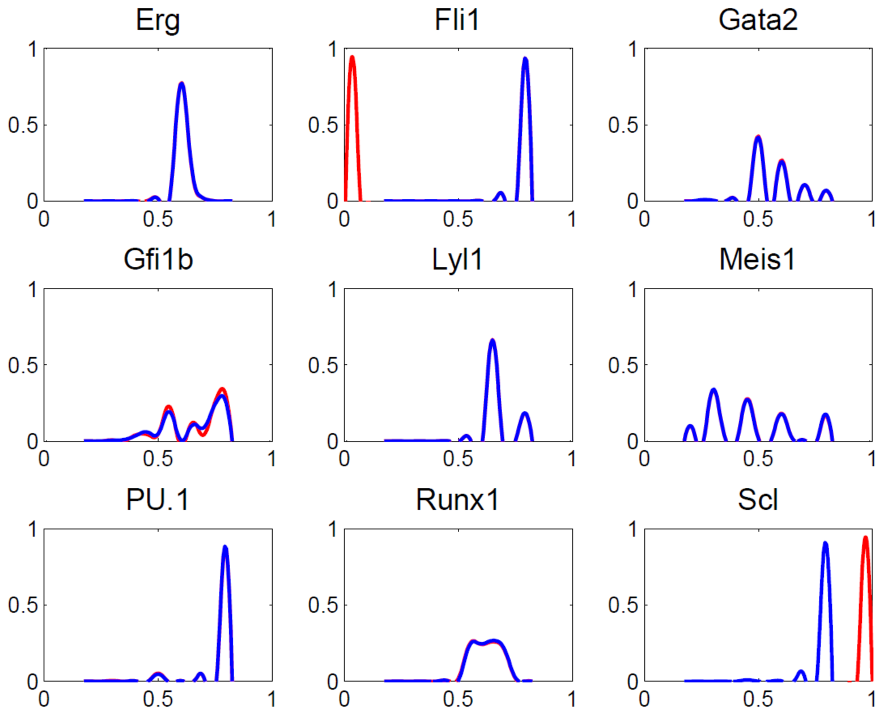
Erg
Fli1
Gata2
Gfi1b
Lyl1
Meis1
PU.1
Runx1
Scl

Top

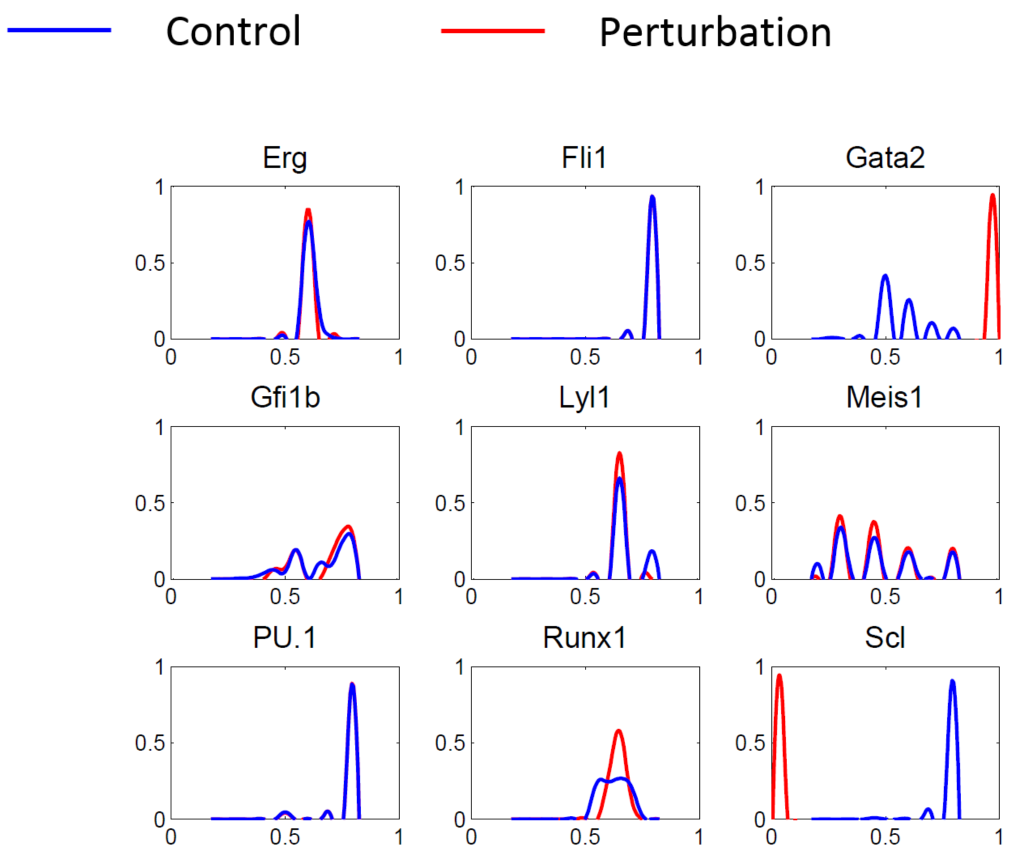

Control
Perturbation
Erg
Fli1
Gata2
Gfi1b
Lyl1
Meis1
PU.1
Runx1
Scl
0
0.5
1

Top

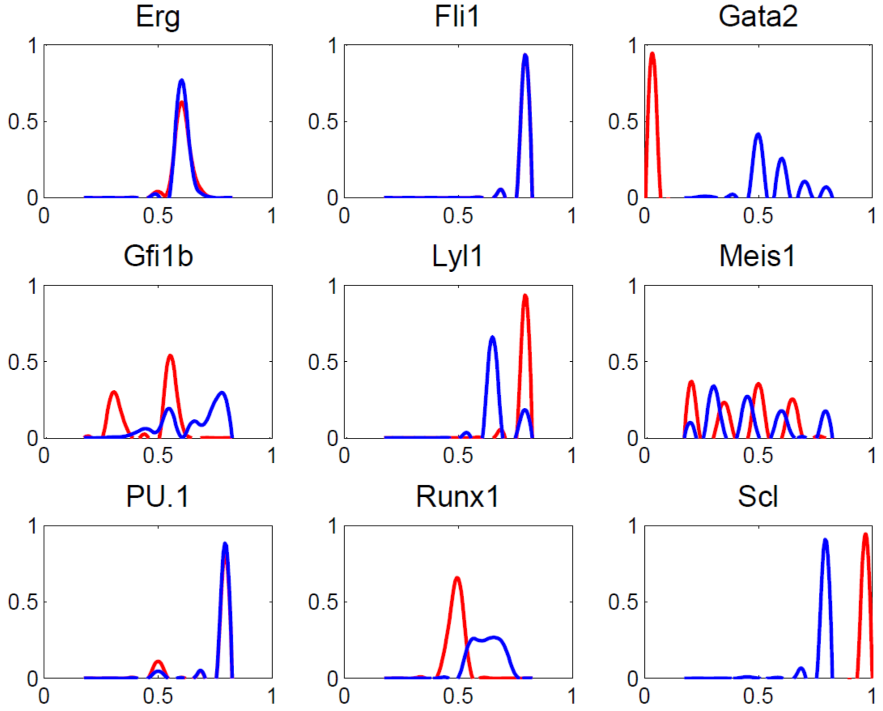

Erg
Fli1
Gata2
Gfi1b
Lyl1
Meis1
PU.1
Runx1
Scl
0
0.5
1

Top

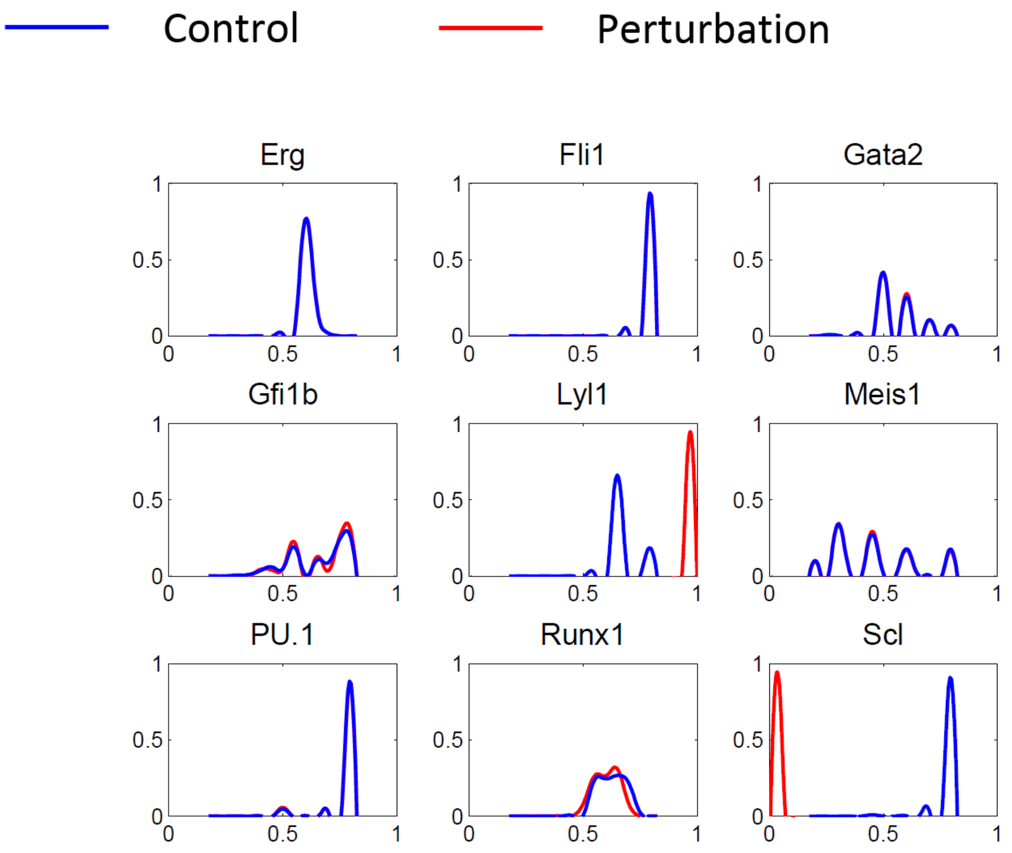
Control
Perturbation
Erg
Fli1
Gata2
Gfi1b
Lyl1
Meis1
PU.1
Runx1
Scl

Top

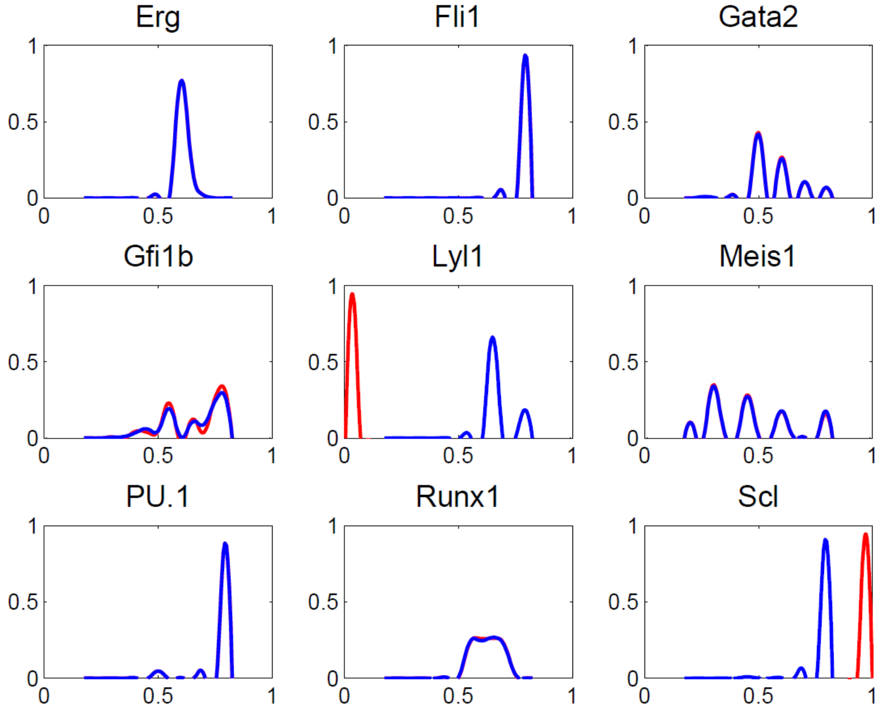
Erg
Fli1
Gata2
Gfi1b
Lyl1
Meis1
PU.1
Runx1
Scl

Top

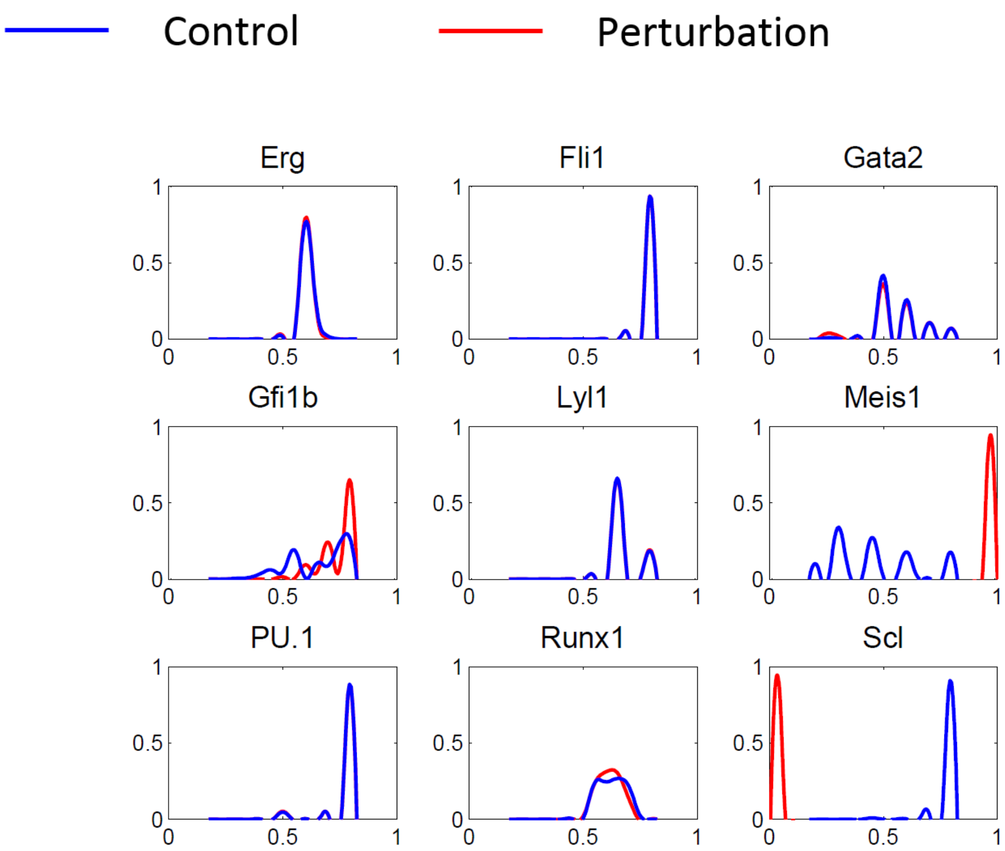
Control
Perturbation
Erg
Fli1
Gata2
Gfi1b
Lyl1
Meis1
PU.1
Runx1
Scl

Top

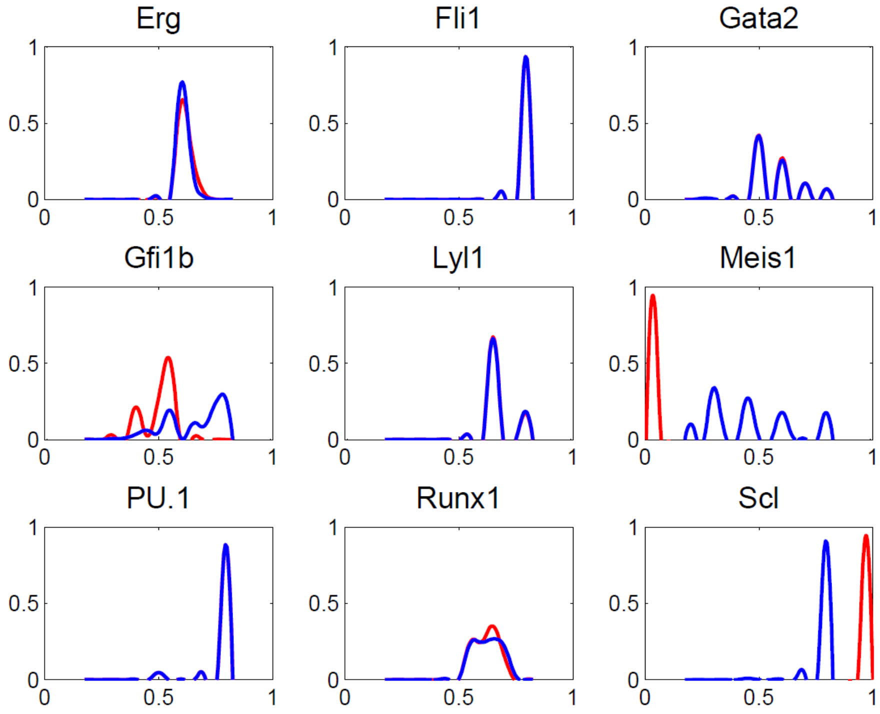
Erg
Fli1
Gata2
Gfi1b
Lyl1
Meis1
PU.1
Runx1
Scl

Top

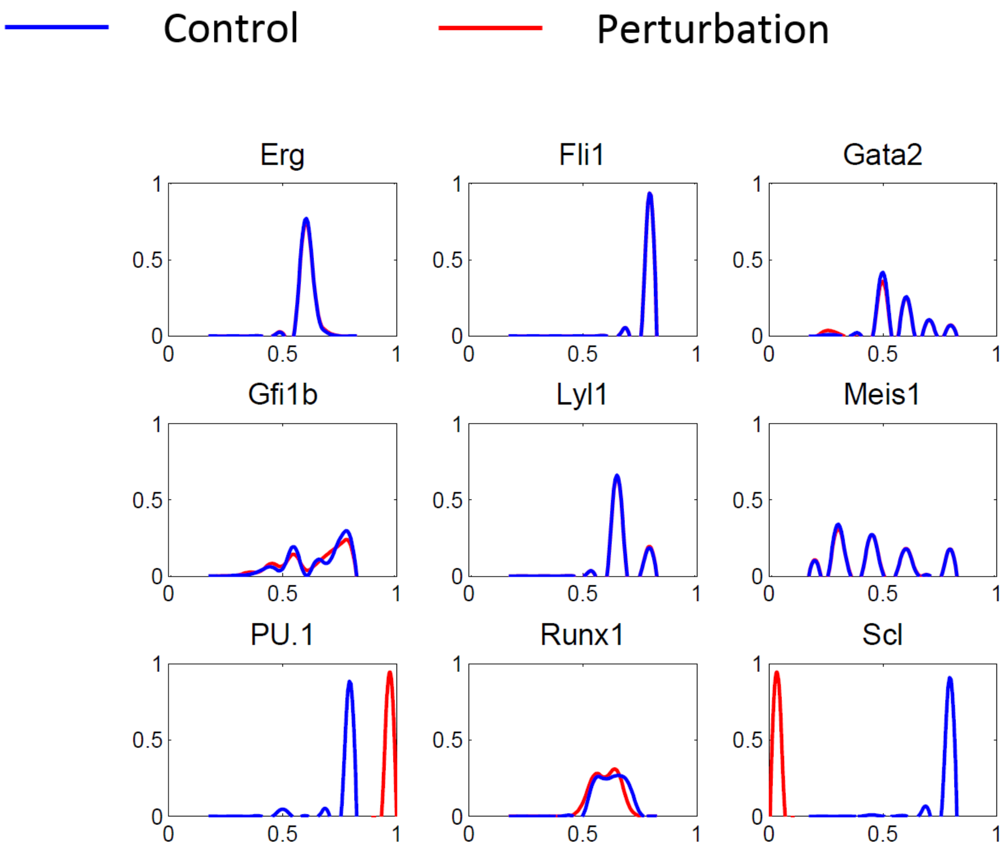
Control
Perturbation
Erg
Fli1
Gata2
Gfi1b
Lyl1
Meis1
PU.1
Runx1
Scl

Top

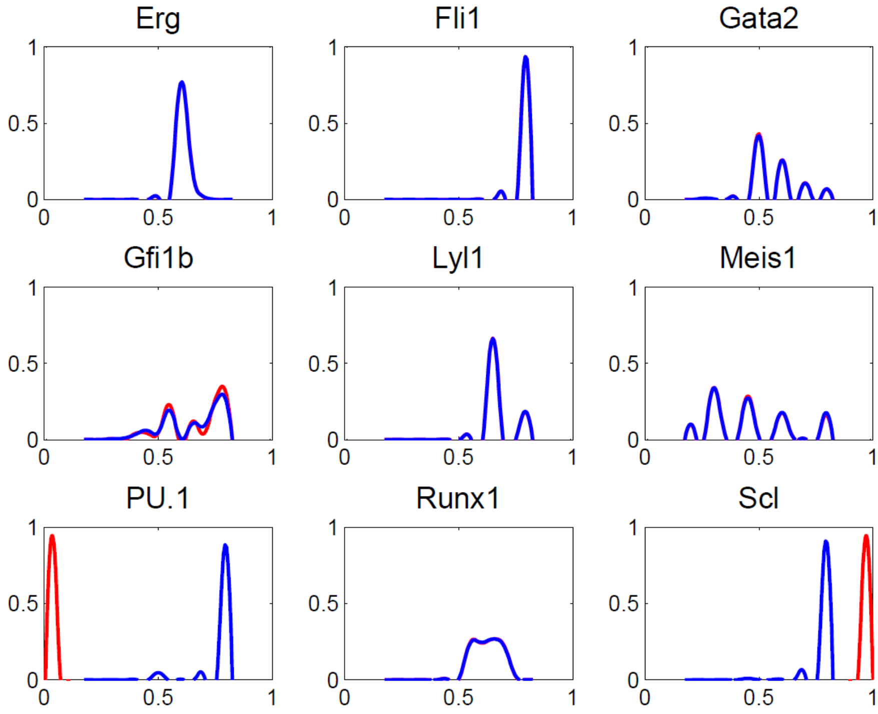
Erg
Fli1
Gata2
Gfi1b
Lyl1
Meis1
PU.1
Runx1
Scl

Top

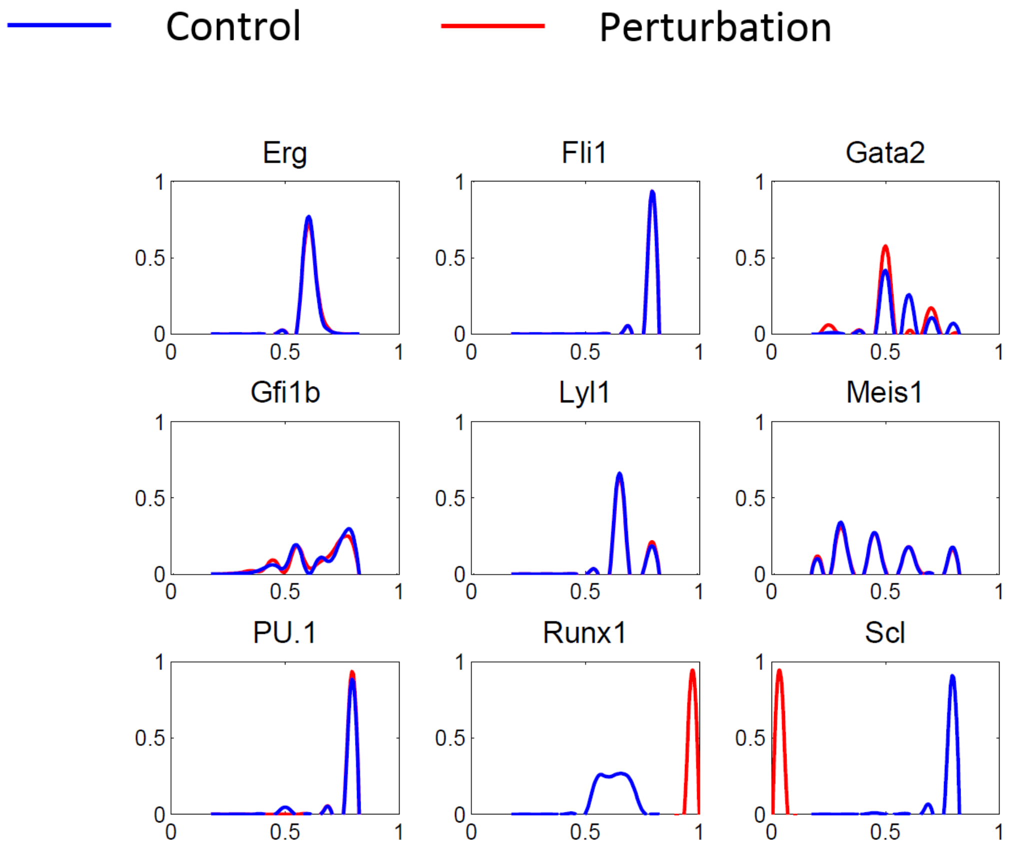
Control
Perturbation
Erg
Fli1
Gata2
Gfi1b
Lyl1
Meis1
PU.1
Runx1
Scl

Top

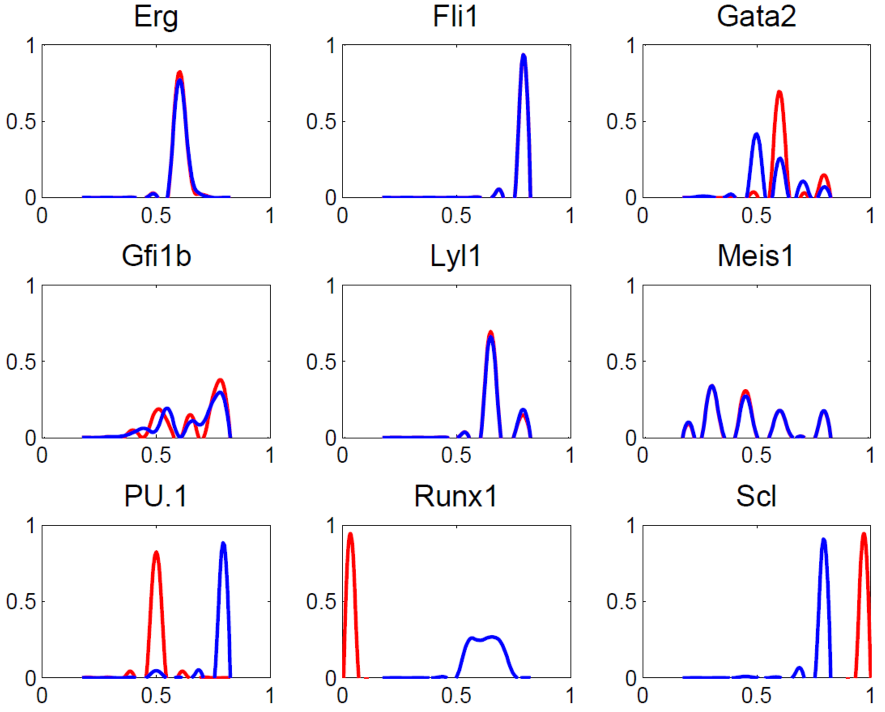
Erg
Fli1
Gata2
Gfi1b
Lyl1
Meis1
PU.1
Runx1
Scl

Top
